# Diabetes treatment for persons with severe mental illness: A registry-based cohort study to explore medication treatment differences for persons with type 2 diabetes with and without severe mental illness

**DOI:** 10.1371/journal.pone.0287017

**Published:** 2023-06-13

**Authors:** Catrine Bakkedal, Frederik Persson, Margit Kriegbaum, John Sahl Andersen, Mia Klinten Grant, Grimur Høgnason Mohr, Bent Struer Lind, Christen Lykkegaard Andersen, Mikkel Bring Christensen, Volkert Siersma, Maarten Pieter Rozing

**Affiliations:** 1 The Research Unit for General Practice and Section of General Practice, Department of Public Health, University of Copenhagen, Copenhagen, Denmark; 2 Complications Research, Steno Diabetes Center Copenhagen, Herlev, Denmark; 3 Centre for Neuropsychiatric Schizophrenia Research, CNSR Mental Health Centre Glostrup, University of Copenhagen, Copenhagen, Denmark; 4 Department of Clinical Biochemistry, Copenhagen University Hospital, Hvidovre, Denmark; 5 Department of Hematology, Copenhagen University Hospital, Rigshospitalet, Copenhagen, Denmark; 6 Department of Clinical Pharmacology, Bispebjerg and Frederiksberg Hospital, Copenhagen, Denmark; 7 Copenhagen Center for Translational Research, Bispebjerg and Frederiksberg Hospital, Copenhagen, Denmark; 8 Department of Clinical Medicine, University of Copenhagen, Copenhagen, Denmark; 9 Department O Rigshospitalet, Psychiatric Center of Copenhagen, Copenhagen, Denmark; Gachon University Gil Medical Center, REPUBLIC OF KOREA

## Abstract

It has been argued that persons with severe mental illness (SMI) receive poorer treatment for somatic comorbidities. This study assesses the treatment rates of glucose-lowering and cardiovascular medications among persons with incident type 2 diabetes (T2D) and SMI compared to persons with T2D without SMI. We identified persons ≥30 years old with incident diabetes (HbA_1c_ ≥ 48 mmol/mol and/or glucose ≥ 11.0 mmol/L) from 2001 through 2015 in the Copenhagen Primary Care Laboratory (CopLab) Database. The SMI group included persons with psychotic, affective, or personality disorders within five years preceding the T2D diagnosis. Using a Poisson regression model, we calculated the adjusted rate ratios (aRR) for the redemption of various glucose-lowering and cardiovascular medications up to ten years after T2D diagnosis. We identified 1,316 persons with T2D and SMI and 41,538 persons with T2D but no SMI. Despite similar glycemic control at diagnosis, persons with SMI redeemed a glucose-lowering medication more often than persons without SMI in the period 0.5–2 years after the T2D diagnosis; for example, the aRR was 1.05 (95% CI 1.00–1.11) in the period 1.5–2 years after the T2D diagnosis. This difference was mainly driven by metformin. In contrast, persons with SMI were less often treated with cardiovascular medications during the first 3 years after T2D diagnosis, e.g., in the period 1.5–2 years after T2D diagnosis, the aRR was 0.96 (95% CI 0.92–0.99). For people with SMI in addition to T2D, metformin is more likely to be used in the initial years after T2D diagnosis, while our results suggest potential room for improvement regarding the use of cardiovascular medications.

## Introduction

Persons with severe mental illness (SMI), such as schizophrenia, bipolar disorder, major depression, or personality disorders, have a 12–20-year shorter life expectancy than the general population [1–3]. Cardiovascular disease is a major contributor to this shortened life expectancy [4–6] and T2D is a well-established risk factor for cardiovascular disease [7]. Notably, 10–-20% of persons with SMI also have type 2 diabetes (T2D), corresponding to a 2–-3-fold increased prevalence compared to the general population [5, 8–10].

Managing T2D involves lifestyle changes, clinical and biochemical monitoring, and most often medications for glycemic control. These activities rely on self-management and might be negatively impacted by SMI [10]. Some previous studies indicate that people with SMI are less likely to receive good T2D treatment [11–17], which might contribute to shorter life expectancy if treatment is inferior in the long term.

The treatment principles for T2D in persons with SMI are the same as in the general population, considering individual characteristics and preferences [5, 18]. The T2D treatments have changed markedly over the last decades with the introduction of new medications and discoveries from research [19]. The importance of treating co-existing risk factors, such as hypertension, dyslipidemia, obesity, and smoking has become increasingly recognized [20].

In Denmark, people with SMI receive treatment for their mental illness in hospital psychiatry, while their somatic chronic care is primarily treated in general practice [21]. More than 80% of people with T2D are taken care of in general practice [22, 23] while only those with severe dysregulation or complications are referred to diabetes specialists, which makes it relevant to explore the treatment in the primary care setting. No studies with a long follow-up have assessed the medication rates for incident T2D in persons with SMI. Therefore, we assessed the use of glucose-lowering and cardiovascular medications in the T2D treatment for persons with and without SMI in general practice using Danish registries.

## Materials and methods

This article is written in accordance with the "Strengthening the Reporting of Observational Studies in Epidemiology (STROBE)" statements [24].

### Study population

A cohort study was established by linking different Danish health registries. Data linkage was done using the unique Civil Registration Number assigned to all Danish residents [25]. All citizens in Denmark have free and direct access to general practitioners, who can refer patients to biochemical testing without individual payment. In Denmark, general practitioners make up 20% of the physician workforce [26] and have a central role in the Danish Health care system, including acting as gatekeepers to more specialised patient care in addition to handling many chronic disorders, including T2D.

The study population consisted of persons with a registered glucose or glycated haemoglobin (HbA_1c_) measurement from the Copenhagen Primary Care Laboratory (CopLab) Database. This database includes test results from the Copenhagen General Practitioners’ Laboratory, which served all primary healthcare practices in the municipality of Copenhagen and the former country of Copenhagen from 2000 to 2015 [27]. The background population of this laboratory covers 1.3 million inhabitants [27] and was accredited by International Organization for Standardization standards ISO17025 and ISO15189. The database does not include glucose point-of-care test results made in general practice.

Persons were included from the day of diabetes diagnosis (index date) defined as the first occurrence of plasma or serum glucose ≥11 mmol/l or HbA_1c_ ≥ 48 mmol/mol (6.5%) in the CopLab Database. We excluded persons living outside the area served by the Copenhagen General Practitioners’ Laboratory. Persons below the age of 30 were excluded to increase the chance of identifying persons with T2D only. Persons with an index date before 1 January 2001 and those with a redeemed prescription of “Drugs used in diabetes” (ATC A10) within two years before the index date were excluded, leaving a cohort of persons with newly diagnosed T2D. Lastly, persons with dementia were excluded (ICD10 F00-F03). The cohort was followed until ten years after the index date, death, emigration out of Denmark, or the end of registrations from the National Prescription Registry (31 Dec 2018) [28], whichever came first.

### Definition of severe mental illness

SMI was identified through diagnostic codes registered in the Danish National Patient Registry [29] and The Danish Psychiatric Central Research Register [30]. The persons were categorized in the SMI group if they had at least one hospital contact with a primary diagnosis of the following ICD-10 codes: Schizophrenia spectrum disorders (F20-F29), bipolar disorder (F30-31), unipolar depression (F32-33), other affective disorders (F34-39) or personality disorders (F60-69) in the five years preceding T2D diagnosis. If a person had several diagnoses in the five years preceding the index date, we used a hierarchy to categorize the person in one group only (schizophrenia spectrum disorders > bipolar disorders > unipolar depression > other affective disorders > personality disorders). In the non-SMI group were people who did not have a primary SMI diagnosis from a hospital admission or visit in the five years before the index date, as listed above.

### Definition of medication use

We retrieved data about medication from the Danish National Prescription Registry, which includes information on all redeemed prescriptions from Danish pharmacies [28]. The variables used are the Anatomical Therapeutic Chemical (ATC) codes and the date of the transaction.

The present study has two overall outcomes as defined by their ATC codes: “Drugs used in diabetes” (A10) and “Cardiovascular medications” (B01AC, C01, C03, C07, C08, C09, C10).

Within the category “Drugs used in diabetes,” we investigated the ATC subgroups “Insulins and analogues” (A10A), “Biguanides” (A10BA) which only includes metformin in the study period, “Sulfonylureas” (A10BB), “Dipeptidyl peptidase 4-inhibitors” (DPP4i) (A10BH), “Glucagon-like peptide 1-receptor agonists” (GLP1-RA) (A10BJ), “Sodium-glucose cotransporter-2 inhibitors” (SGLT2i) (A10BK) and “Combinations of oral glucose-lowering drugs” (A10BD).

For the cardiovascular medications, we investigated the following classes: “Platelet aggregation inhibitors” (B01AC), “Cardiac therapy, e.g., cardiac glycosides and anti-arrhythmic” (C01), “Diuretics” (C03), “Beta-blocking agents” (C07), “Calcium channel blockers” (C08), “Agents acting on the renin-angiotensin system” (C09) and “Lipid-modifying agents” (C10).

### Covariates

We used the following categories for HbA_1c_ levels at index: 48–52.9 mmol/mol, 53–57.9 mmol/mol, 58–69.9 mmol/mol and ≥70 mmol/mol. If HbA_1c_ was not measured persons were categorized into a group defined by plasma or serum glucose ≥ 11 mmol/L.

The concentration in blood of total cholesterol, low-density lipoprotein (LDL)-cholesterol, high-density lipoprotein (HDL)-cholesterol, triglycerides, creatinine (used to calculate the estimated glomerular filtration rate (eGFR) [31]), and urine albumin-creatinine ratio was identified from the CopLab database up to 30 days before or 14 days after the index. Only a subset of the study population had test measurements for these parameters. A median of the results was used if a person had more than one measurement. The blood tests were performed as previously described [32]. The urine albumin-creatinine ratio was measured in spot urine with the commercially available assay Advia 1650/Advia2400 (Bayer, Siemens, Healthcare Diagnostics, Tarrytown, NY, USA) according to the instructions of the manufacturers.

Comorbidities included in the analyses were atherosclerotic cardiovascular disease, heart failure and moderate/severe renal disease and have been identified using the Danish National Patient Registry [29]. We defined comorbidity as a hospital admission or visit with any of the diagnoses shown in the S1 Table from the year 1990 until the index date.

We also used Charlson’s Comorbidity Index to describe somatic comorbidity. The index is the sum of 19 chronic conditions weighted by an assessment of their relative severity; it is a reliable predictor of mortality and has been validated in various settings [33]. Data on hospital admissions or visits with the diagnoses included in Charlson’s Comorbidity Index were retrieved from the Danish National Patient Registry [29].

The Population Education Register provided information about the highest retrieved level of education [34] within five years before the index date. The level of education was mapped according to the international standard classification of education ISCED (1997) codes. Up to 10 years of education (ISCED level 0–2), 11–12 years of education (ISCED level 3), and 13 or more years of education (ISCED level 4–6). The group with missing education were treated separately in the analyses.

The population register provided data about the marital status on 1 January the year of the index date.

Substance abuse was defined as a hospital admission or visit with a primary diagnosis of F10-F19 (ICD-10) within five years before the index date in the Danish National Patient Registry [29] or Danish Psychiatric Central Research Register [30].

### Statistical analyses

The analyses assess prescription redemptions for each ATC group during the first ten years after T2D diagnosis in periods of six months for the group with SMI compared to the group without SMI. We chose 6-month periods because if medication treatment is stable, persons typically receive three months’ supply on each prescription [28]. In this way, persons who might not take medications daily were still counted as in treatment. The fraction of persons in treatment with a certain medication in each period of six months after their T2D diagnosis is calculated for the SMI and non-SMI groups as a rate, i.e., the number of persons redeeming at least one prescription in each 6-month period divided by the sum of the patient risk time in that period. The latter was cut short if the person died, migrated out of Denmark, or reached the end of the Danish National Prescription Register (31 December 2018). Furthermore, time at risk of fewer than six months occurred if this period started before the year a medication group was available on the market; most medication groups were available throughout the full study period except “Combinations for oral glucose-lowering medications (2004), DPP-4i and GLP1-RA (2007) and SGLT2i (2013) [35].

The differences in treatment rates between the SMI and the non-SMI group were assessed as rate ratios (RR) from Poisson regression models. These RRs were adjusted for a possible reduced time at risk for the persons in the various 6-month periods by including the logarithm of the time at risk as an offset. To account for the dependence between periods from the same person, we used the method of generalized estimating equations with a robust variance estimator using an independent working correlation. To adjust for confounding we adjusted the analyses for sex, age (quadratic), glycaemic control at the index date, comorbidities preceding the index date (S1 Table), calendar year and level of education. Crude fractions of persons who redeem medications are presented in S1 and S2 Figs. Statistical analyses were done in SAS v9.40. A p-value of 0.05 was considered statistically significant.

### Sensitivity and stratified analyses

Significant determinants for pharmacological cardiovascular treatment include diagnoses like atherosclerotic cardiovascular disease, heart failure, or renal disease. Therefore, we conducted a sensitivity analysis in which the analysis was repeated in the subcohort of the study population who had a history of any of these comorbidities (see S1 Table for ICD-10 codes) in addition to T2D. In supporting information S3 and S4 Figs (crude fractions) and S5 and S6 Figs (adjusted rates), we stratified into SMI-subgroups.

### Ethics

As required by the General Data Protection Regulation, the Records of data processing activities at the University of Copenhagen approved the study (514-0361/19-3000), and data access was allowed by Statistics Denmark and the Danish Health Data Authority (FSEID-00004891/DST-project number 707726). For registry-based studies, approval by the Ethics Committee and written informed consent are not necessarily due to Danish legislation. All results are presented at the aggregate level following guidelines from Statistics Denmark.

## Results

### Baseline characteristics

**Fig 1:**
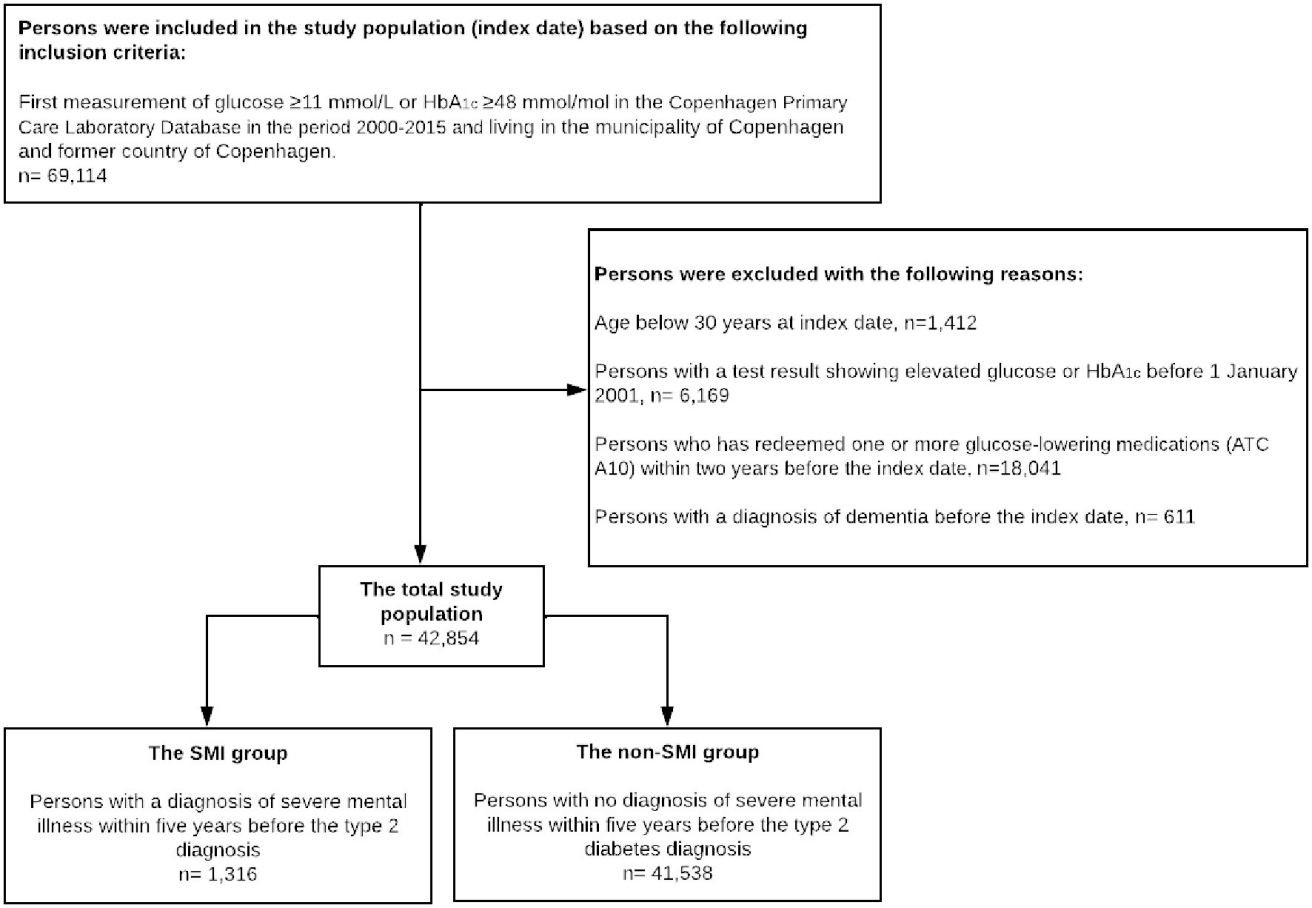
Flowchart of the study population. HbA_1c_ = glycated haemoglobin, ATC = anatomical therapeutic classification, SMI = severe mental illness.

In total, 42,854 persons with T2D were included in the analyses (Fig 1). The SMI group included 1,316 persons (3.1%). Baseline characteristics are presented in Table 1. Most SMI diagnoses were within the schizophrenia spectrum (49%) or unipolar depression (34%). A higher proportion of persons in the SMI group were included in the study population in the period 2011–2015 (40% versus 32% in the non-SMI group). The median follow-up time was 6.5 (IQR 3.9–10.0) years in the SMI group and 8.1 (IQR 4.3–10.0) years in the non-SMI group. Persons in the SMI group were younger, and more were females.

**Table 1:** Baseline characteristics.

	Severe mental illness	No severe mental illness
	(n = 1,316)	(n = 41,538)
Age, years, median (IQR^a^)	55.0 (46.4–65.1)	62.4 (53.2–72.0)
Males, n (%)	608 (46)	23,659 (55)
Year of diabetes diagnosis, n (%)		
2001–2005	374 (28)	15,072 (36)
2006–2010	417 (32)	13,714 (32)
2011–2015	525 (40)	13,694 (32)
Type of severe mental illness, n (%)		
Schizophrenia spectrum (F20-29^b^)	651 (49.5)	-
Bipolar disorders (F30-31^b^)	126 (9.6)	-
Unipolar depression (F32-33^b^)	450 (34.2)	-
Other affective disorders (F34-39^b^)	13 (1.0)	-
Personality disorders (F60-69^b^)	76 (5.8)	-
Substance abuse (F10-19^b^), n (%)	115 (9)	562 (1)
Marital status, n (%)		
Divorced	356 (27)	7474 (18)
Married	347 (26)	22553 (54)
Unmarried	471 (36)	5550 (13)
Widower	142 (11)	5961 (14)
Education, n (%)		
< 10 years of education	563 (43)	14454 (35)
11–12 years of education	418 (32)	16158 (39)
≥13 years of education	233 (18)	6984 (17)
No information	102 (8)	3942 (9)
Charlson Comorbidity Index, n (%)		
0	875 (66)	29574 (71)
1–2	347 (26)	9522 (23)
3–4	73 (6)	1872 (5)
≥5	21 (2)	570 (1)
Comorbidity, n (%)		
Atherosclerotic cardiovascular disease	247 (19)	9114 (22)
Heart failure	77 (6)	2743 (7)
Renal disease	31 (2)	750 (2)
Atherosclerotic cardiovascular disease, heart failure or renal disease	296 (22)	10329 (25)
Glycaemic control at the index		
Glucose >11 mmol/L, n (%)	344 (26)	8971 (22)
HbA_1c_, mmol/mol, median (IQR)	52 (49, 62)	52(49, 61)
Total cholesterol (mmol/L)		
Median (IQR)	5.5 (4.7, 6.4)	5.4 (4.6, 6.2)
Sample size, n	903	29,955
Low-density lipoprotein cholesterol (mmol/L)		
Median (IQR)	3.1 (2.5, 3.8)	3.1 (2.4, 3.8)
Sample size, n	660	23,138
High-density lipoprotein cholesterol (mmol/L)		
Median (IQR)	1.1 (0.9, 1.3)	1.2 (1.0, 1.4)
Sample size, n	809	26,791
Triglycerides (mmol/L)		
Median (IQR)	2.3 (1.6, 3.6)	1.9 (1.4, 2.8)
>2.5 mmol/L, n (%)	364 (46)	8491 (32)
Sample size, n	785	26,192
Estimated glomerular filtration rate (mL/min/1.73 m^2^)		
Median (IQR)	97 (88,105)	93 (84, 101)
Sample size, n	957	29,671
Urine albumin-creatinine ratio (mg/g)		
Median (IQR)	27.0 (15, 73)	29.5 (16, 74)
Sample size, n	102	3,906

^a^ IQR = interquartile range,

^b^ ICD-10-codes,

^c^ ICD-8 and ICD-10 codes are presented in supporting information S1 Table.

Persons with SMI had more comorbidity compared to the non-SMI group (Charlson Comorbidity Index > 0 for 34% and 29%, respectively). About one-fifth of persons in both groups had a diagnosis of atherosclerotic cardiovascular disease before the index (19% in the SMI group and 22% in the non-SMI group). Substance abuse diagnoses were more common in the SMI group than in the non-SMI group (9% versus 1%). Persons in the SMI group were more often either divorced, unmarried, or widowed (74%), compared to the non-SMI group (46%). A higher proportion of persons in the SMI group had less than 10 years of education at the index (43% versus 35%).

Median HbA_1c_, total cholesterol, LDL-cholesterol, eGFR and urine albumin-creatinine ratio were approximately similar for the two groups at baseline. Persons with SMI generally had higher levels of triglycerides. A higher proportion of persons in the SMI group (45%) had low HDL-cholesterol compared to the non-SMI group (35%) when low HDL-cholesterol was defined as < 1 mmol/L for men and < 1.2 mmol/L for women.

### Analyses of glucose-lowering medication classes

**Fig 2:**
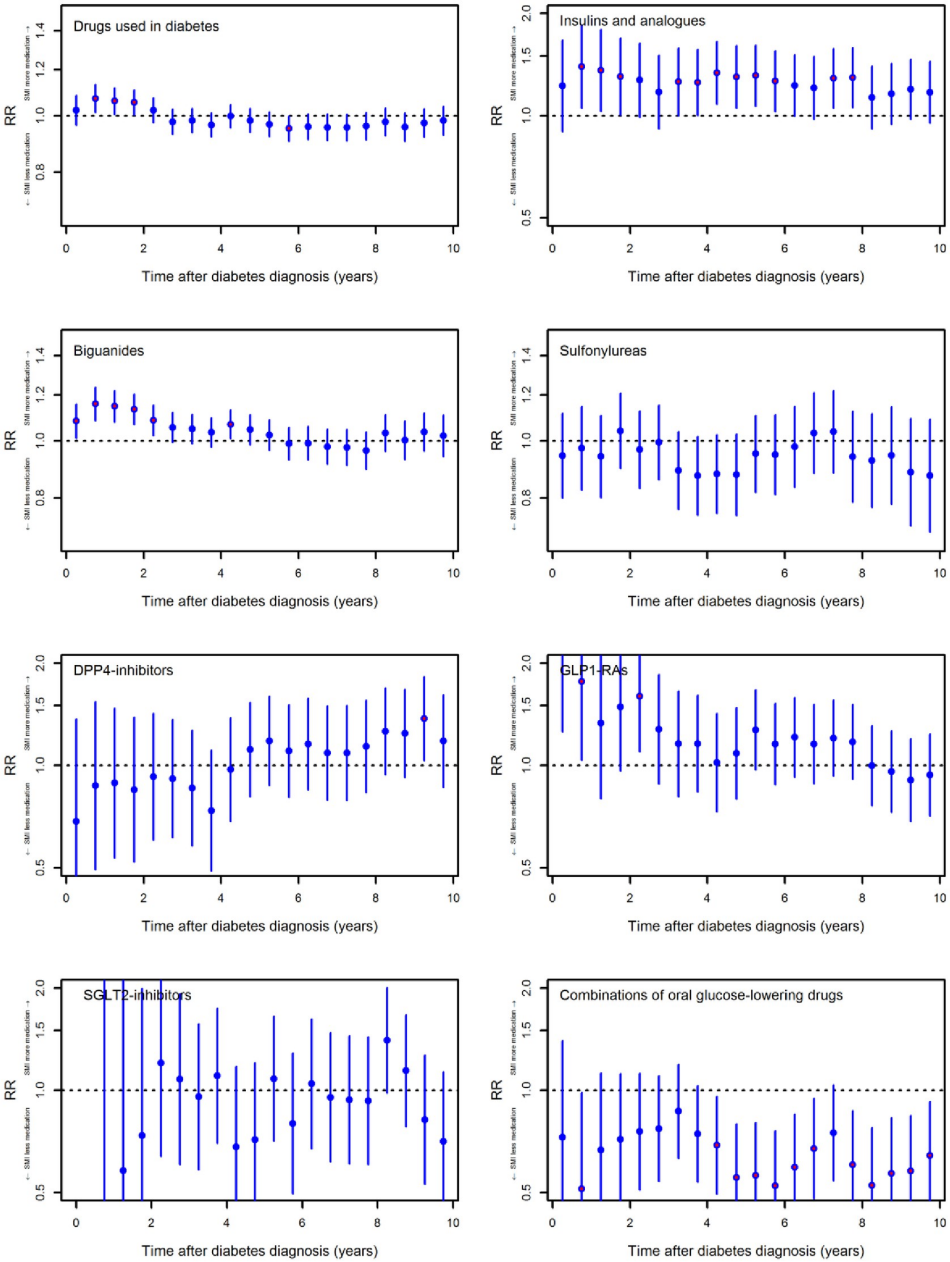
Redemption rates of glucose-lowering medications for persons with T2D and SMI versus those without SMI. Data are presented as rate ratios (RR) adjusted for sex, age (quadratic), glycaemic control at index, comorbidities preceding the index, calendar year and level of education. Each bar represents the estimate and 95% confidence interval for each period á six months. DPP4-inhibitors = dipeptidyl peptidase 4 inhibitors, GLP1-RAs = glucagon-like peptide 1 rector agonists, SGLT2-inhibitors = sodium = glucose cotransporter 2 inhibitors.

“Drugs used in diabetes” were redeemed more frequently in the SMI group approximately two years after the index compared to the group without SMI (Fig 2). The difference in treatment rates was significant for three 6-month periods within the first few years. During the remaining follow-up period, the point estimates showed a marginally lower use in the SMI group, albeit the difference in the fraction of persons redeeming treatment was mostly statistically non-significant.

For the individual medication classes within “Drugs used in diabetes”, we found that during follow-up more persons in the SMI group redeemed insulin and analogues compared to the non-SMI group. Biguanides were used more frequently in the SMI group in the initial 2.5 years, and thereafter we observed no difference between the two groups. For sulfonylureas, the treatment rates were generally lower in the SMI group during the follow-up, although the differences were statistically non-significant. Persons in the SMI group had higher treatment rates for GLP1-RA in the initial years after the index, but the difference equalized during the follow-up period. For DPP-4i and SGLT2i it was not possible to describe a trend from our data due to wide confidence intervals. Persons with SMI more seldom redeemed “Combinations of oral glucose-lowering drugs” compared to persons without SMI. The difference was significant five years after the diagnosis of T2D. Crude and adjusted RRs are presented in the supporting information (S2 and S3 Tables). Stratification of the analyses for individual psychiatric diagnoses showed some heterogeneity between the subgroups, i.e., the increased use of metformin initially was primarily driven by a higher use among people with schizophrenia spectrum disorders and bipolar disorders (S5 Fig).

### Analyses of cardiovascular medication classes

**Fig 3:**
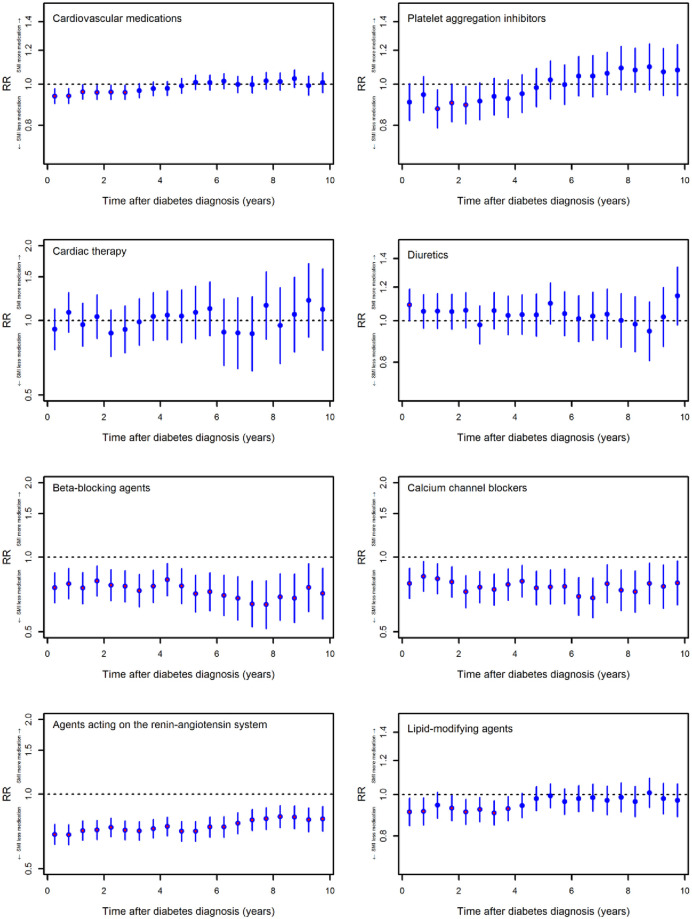
Redemption rates of cardiovascular medications for persons with T2D and SMI versus those without SMI. Data are presented as rate ratios (RR) adjusted for sex, age (quadratic), glycaemic control at index, comorbidities preceding the index, calendar year and level of education. Each bar represents the estimate and 95% confidence interval for each period a six months.

Persons with SMI had lower use of “Cardiovascular medications” compared to the non-SMI group in the first approximately three years after the index (Fig 3). Thereafter, there was no statistically significant difference.

For the individual medication classes, we found that beta-blocking agents, calcium channel blockers, and agents acting on the renin-angiotensin system (ACEi/ARBs) were used more rarely in the SMI group during the complete follow-up. Lipid-modifying agents were redeemed more seldom in the SMI group than in the non-SMI group in the first approximately four years after the index (Fig 3).

The sensitivity analysis does not change the interpretation of the results from the main analysis. When comparing individuals with and without SMI in the subcohort with established cardiovascular diseases, heart failure or renal disease, we found no discernible trend for differences in the overall outcome ("Cardiovascular medications"), which indicates that these comorbidities are an artefact in the overall main analysis for this outcome. When assessing the individual cardiovascular medication classes (lipid-lowering drugs, calcium antagonists, beta-blockers, and ACEi/ARBs) in this subcohort, we found that people with SMI continue to receive these medications less frequently than people without SMI.

The stratified analysis showed that the reduced use of cardiovascular medications in the initial years was due to lower use among people with schizophrenia spectrum disorders, bipolar disorders, and personality disorders but not among people with unipolar depression (S6 Fig). For the individual cardiovascular medication classes, the same tendency was seen, i.e., when reduced use was present, it was mainly associated with schizophrenia spectrum disorders and bipolar disorders, but less so with unipolar depression.

## Discussion

In this observational cohort study, we found that persons with T2D and SMI had higher treatment rates for glucose-lowering medications overall in the initial years after diabetes diagnosis compared to persons without SMI. The opposite was observed for most cardiovascular medications, where persons in the SMI group more rarely redeemed a prescription.

### Differences in the use of glucose-lowering medications

Several studies conclude that persons with SMI are under-treated with glucose-lowering medications [11–17]. Contrastingly, we found that persons with concomitant SMI and T2D are marginally more likely than persons without SMI to redeem glucose-lowering medications in the initial years after T2D diagnosis despite similar glycaemic control at index. Differences in study design might explain this difference. First, most studies do not incorporate diabetes duration but instead provide cross-sectional treatment rates for persons, who have had diabetes for years [11, 13, 14]. Secondly, some studies do not distinguish if a low treatment rate is due to undiagnosed diabetes [13, 14]. Thirdly, some studies that report low treatment rates in persons with SMI compare the actual antidiabetic treatment to guideline recommendations [17] or self-reported diagnoses [15]. Lastly, our results may diverge from previous results due to differences in healthcare systems across countries.

Our results suggest that patients and physicians are often more willing to initiate glucose-lowering medications soon after diagnosis in the SMI group. This is in alignment with another Danish register-based study, where persons with depression were more likely to initiate glucose-lowering medications in the first year after T2D diagnosis compared to persons without depression [36]. Previous studies found that persons with schizophrenia [37] or depression [36] are more adherent to glucose-lowering medications than the general population, which indicates that these medications are accepted among persons with SMI. Our study does not show whether the implementation of lifestyle changes differed between the groups, or whether the SMI group had more T2D complications at the index date which could require earlier intensification in treatment, however, we found similar glycaemic control at the index, and we may speculate whether the initial difference in treatment rates can be due to clinical inertia [38, 39].

The higher use of glucose-lowering medications initially after diagnosis in the SMI group is primarily due to metformin, which has been the first-line medication for persons with T2D and obesity since the late 1990s [40] and since 2005 for all persons with T2D after recommendation by the International Diabetes Foundation [41]. In Denmark, metformin was the first-choice medication for 30% of persons with T2D in the year 2000, while this percentage increased to 87% in 2009 [42]. In this present study, more persons with SMI were included in the study population from the year 2011 through 2015, and the increase in the use of metformin across the study period may partly reflect the higher treatment rates in the SMI group, despite that we have mitigated this effect by adjusting for the calendar year. Rapid initiation of metformin after diagnosis may be a rational choice as many persons in the SMI group may be obese [10], and metformin can attenuate weight gain.

Hypoglycaemia should be avoided for persons with T2D. It can be argued that this is especially true for persons living alone or those who cannot react adequately to symptoms i.e., due to psychiatric symptoms [18]. We found that persons with SMI more often redeemed insulins during the 10 years of follow-up compared to persons without SMI. Insulin is the most efficacious in terms of glucose-lowering potential but can increase weight and is associated with the risk of hypoglycaemia [18]. We found similar and relatively low HbA_1c_ levels at index in both groups, thus poorer glycemic control does not seem to explain the higher use of insulin for persons with SMI. The SMI group may have included more people with late-onset type 1 diabetes, but we believe this is unlikely because T2D is much more common after the age of 30, and the SMI group’s mean age at the index date was 55 years. In addition, for persons with type 1 diabetes, insulin should be started soon after diagnosis, and we found that insulin was used by only a small fraction of persons in the first six months after diagnosis, which also indicates that the number of patients with type 1 diabetes is low in our study population (S1 Fig). Studies from the US and Germany have also found that persons with T2D and SMI more often use insulins [43, 44]. Future studies should include information regarding the type of insulin, as long-lasting insulins have less risk of hypoglycemia.

Since the year 2015, when the present study ended, SGLT2i and GLP1-RA have gained a stronger position in diabetes guidelines as clinical trials have shown renal and cardiovascular advantages for these medications after their marketing. Our study shows that the novel and more expensive medications (GLP1-RA, DPP4-i, SGLT2i) up to 2015, were used by a small proportion of persons in both groups only (S1 Fig). However, when compared to the non-SMI group, people with SMI redeemed GLP1-RAs more frequently soon after being diagnosed with T2D. GLP1-RAs may be a rational choice despite the highest price among the glucose-lowering medications, especially due to the renal and cardiovascular advantages discovered by the end of the study period, as mentioned above, but also because GLP1-RAs can promote weight loss and provide no risk of hypoglycaemia [18]. A recently published Danish registry study found that the risk of nephropathy and cardiovascular complications is higher for persons with SMI at a younger age compared to persons without SMI, and this highlights the need to use preventive medications early [45].

Combination preparations, which contain metformin and either thiazolidinedione, DPP4i, or SGLT2i, or the combination of SGLT2i and DPP4i (32), were prescribed less often in the SMI group compared to the non-SMI group in this study. For some people, it can be more convenient to use combination preparations instead of single tablets, but combination preparations are generally expensive, and medication is not fully reimbursed in Denmark.

### Differences in the use of cardiovascular medications

Dyslipidemia and hypertension are risk factors for cardiovascular disease, which can be diminished by lipid-lowering medications and antihypertensives [46]. We found lower use of lipid-lowering medications in the SMI group in the first four years after the index date, despite the median LDL-cholesterol levels being comparable at the time for T2D diagnosis in the two groups. Our findings do not show how many people were already taking lipid-lowering medications before being diagnosed with T2D. Previous studies concur that dyslipidemia is frequently untreated in patients with SMI [13, 17, 47, 48], who frequently have dyslipidemia [5]. In the European guidelines for dyslipidemia, people with SMI are now considered a risk group, because they are more likely to get atherosclerotic cardiovascular disease, and lifestyle changes and taking statins are therefore emphasized [49]. Many people believe that statins frequently cause muscular symptoms [50] and restricting sources of such body symptoms may contribute to limited statin use, especially in the SMI population.

In terms of hypertension treatment, medicines that act on the renin-angiotensin system (ACEi and ARBs) are currently and have been for most of the study period, regarded as first-line medications for individuals with T2D. Our findings show that people with SMI use ACEi or ARBs less frequently. Another often used antihypertensive is thiazides (included in the "Diuretics”). For diuretics, we found no difference in treatment rates between the two groups. Alternative antihypertensives, such as calcium-channel blockers and beta-blocker medicines, were used more rarely in the SMI group too. Our registries do not contain information about the prevalence of hypertension in the two groups, and therefore, our findings may be due to the underdiagnosis of hypertension in the SMI group. Previous studies found a similar or higher prevalence of hypertension among persons with SMI compared to the general population or persons without SMI [5, 51]; for example, in a Danish cross-sectional study 55% of people on antipsychotic medication had hypertension [51]. This implies that people with SMI in our study may be undertreated for hypertension, although antipsychotics have blood-pressure-lowering side effects, which may explain some of the lower use of cardiovascular medications [52]. Previous research has revealed that despite indications, many people with SMI were not treated for hypertension [11, 13, 14, 17]. This possible undertreatment, which we found for most cardiovascular medications, has previously been seen in Danish registry studies for both primary and secondary prevention with cardiovascular medications among people with SMI [52, 53]. We can only speculate as to why glucose-lowering medicines may be prioritized above cardiovascular therapy, but our results are in line with other studies indicating that hypertension and dyslipidemia are left untreated in the SMI group more frequently than hyperglycemia [13, 15, 17]. In the CATIE trial, for example, 30% of individuals with schizophrenia were untreated for diabetes, compared to 62% and 88%, respectively, for hypertension and dyslipidemia [13]. An argument for not treating the cardiac risk factors could be that the patient and/or physician want to prevent polypharmacy, drug interactions, or adverse effects. The argument for a glucose-lowering impact may appear more logical than treating cardiac risk factors when benefits take time to manifest. In a qualitative study conducted in the United Kingdom, several persons with coexisting SMI and T2D, as well as their primary care physicians, were pessimistic about whether the patient lived long enough to prevent complications [54]. Another possible explanation could be that, in the past, hyperglycemia was the primary focus of treatment for T2D, but there has been a shift toward a strategy that puts more emphasis on treating cardiac risk factors as well. Our results may reflect a somehow slower transition in the SMI group.

### Strengths and limitations

This study is unique as it focuses on the ten years follow-up period after diagnosis for T2D in a representative population of persons with SMI in a real-world setting. All outcomes are based on prescription redemptions. Pharmacies in Denmark are obliged to register all dispensed prescriptions due to partial reimbursement of medication expenses by the government-financed healthcare system. This ensures that the registry is valid and complete, and the risk of detection bias is minimal.

Despite these strengths, the found treatment rates should be interpreted with caution. It was unknown whether plasma or serum glucose was measured during fasting, and therefore persons with glucose ≥ 7.0 mmol/L, but < 11.0 mmol/L were not included. We also did not include oral glucose tolerance tests in the diagnostic criteria, but these are relatively rarely performed in general practice.

The study population is confined to the greater Copenhagen area. There is a geographic variation between regions concerning the choice of diabetes medications [42, 55]. However, to our knowledge, no studies have investigated whether the presence of SMI shows geographical variation in pharmacological treatments too.

Some of the variables that we adjust for can vary by time (i.e., glycaemic control, comorbidities, educational level), however, we have adjusted according to baseline values for simplicity. SMI status, before the index date, is used in the analyses, albeit the persons could have been diagnosed or undiagnosed during the follow-up, which we believe will lead to more conservative estimates of differences between the groups. We observed higher mortality in the SMI group, which will likely lead to a selection of the more "robust" persons in the SMI group later in the follow-up. While mortality is not high and the influence of such selection is limited, the higher mortality in the SMI group would, if anything, probably lead to lower medicine use. Another limitation is the lack of data in the databases regarding lifestyle interventions and blood pressure. As the CopLab database includes data until the year 2015, this study is unable to investigate treatment rates thereafter. However, the historical treatment rates, and the differences between the SMI and non-SMI groups in this regard, are important to improve our understanding of the complexity of the reduced life expectancy for persons with SMI.

From a clinical standpoint, our results indicate that among people with pre-existing SMI, metformin is generally used more in the initial years after the T2D diagnosis. The apparently opposing results regarding CVD prescribing suggest that there might be room for improvements in the treatment of cardiac risk factors (i.e., hypertension and dyslipidemia) among people with SMI and T2D. Further studies are warranted to explore if and why glucose-lowering medications are prioritized higher than CV medications among people with SMI; this can be done with qualitative studies, for example.

## Conclusion

We found that in the initial years after T2D diagnosis persons with SMI more often redeem glucose-lowering medications than persons in the non-SMI group. In contrast, persons with SMI are less likely to redeem prescriptions for other cardiovascular preventive medications, i.e., ACEi/ARBs, beta-blocking agents, calcium channel blockers and lipid-lowering medications than persons in the non-SMI group.

## Supporting information

S1 Table Comorbidities. Categorized based on International Statistical Classification System of Diseases editions 8 and 10. Each of these conditions is seen as a possible confounding factor and included in the statistical analyses.
(DOCX)

S2 Table Crude and adjusted rate ratios for glucose-lowering medications. Bold text indicates where the difference is statistically significant at the 0.05 level. RR = rate ratio. ATC (anatomical therapeutic classification) codes are presented in parentheses. DPP4i = dipeptidyl peptidase 4 inhibitors. GLP1-RAs = glucagon-like peptide 1 receptor agonists, SGLT2i = sodium-glucose cotransporter 2 inhibitors.
(DOCX)

S3 Table Crude and adjusted rate ratios for cardiovascular medications. Bold text indicates where the difference is statistically significant at the 0.05 level. RR = rate ratio. ATC (anatomical therapeutic classification) codes are presented in parentheses.
(DOCX)

S1 Fig Crude fractions of persons who have redeemed one or more prescriptions of a glucose-lowering medication within a period of 6 months. Each figure shows the fraction of persons with severe mental illness (SMI) versus persons without severe mental illness (non-SMI), with a follow-up of 10 years after diabetes diagnosis divided into six-months periods. ATC (anatomical therapeutic classification) codes can be seen in the manuscript. DPP4-inhibitors = dipeptidyl peptidase 4 inhibitors. GLP1-RAs = glucagon-like peptide 1 receptor agonists, SGLT2-inhibitors = sodium-glucose cotransporter 2 inhibitors.
(DOCX)

S2 Fig Crude fractions of persons who have redeemed one or more prescriptions of a cardiovascular medication within a period of 6 months. Each figure shows the fractions of patients with severe mental illness (SMI) versus patients without severe mental illness (non-SMI) with a follow-up of 10 years after diabetes diagnosis divided into six-months periods. ATC (anatomical therapeutic classification) codes are presented in the manuscript.
(DOCX)

S3 Fig Crude fraction of glucose-lowering medication use stratified for SMI-subgroups. (DOCX)

S4 Fig Crude fraction of cardiovascular medication use stratified for SMI-subgroups. (DOCX)

S5 Fig Adjusted rate ratios for glucose-lowering medications stratified for SMI subgroups. (DOCX)

S6 Fig Adjusted rate ratios for cardiovascular medications stratified for SMI subgroups. (DOCX)
