# Supplementary material for: Diabetes treatment for persons with severe mental illness: A registry-based cohort study to explore medication treatment differences for persons with type 2 diabetes with and without severe mental illness
Source: PLoS One. 2023 Jun 13;18(6):e0287017. doi: 10.1371/journal.pone.0287017 (PMC10263345; doi:10.1371/journal.pone.0287017)
Supplement: S1 Table — Categorized based on International Statistical Classification System of Diseases editions 8 and 10. Each of these conditions is seen as a possible confounding factor and included in the statistical analyses. (DOCX) [file pone.0287017.s001.docx]

**S1 Table. Comorbidities.** Categorized based on International Statistical Classification System of Diseases editions 8 and 10. Each of these conditions is seen as a possible confounding factor and included in the statistical analyses.

| Atherosclerotic cardiovascular disease | Myocardial infarction | ICD 8: 410  ICD10: I21 |
| --- | --- | --- |
|  | Coronary revascularization | Procedure codes: KFNG KFNF KFNA KFNB KFNC KFND KFNE KFNH20 |
|  | Stable/unstable angina | ICD8: 411 413  ICD10: I20 I251 I259 |
|  | Peripheral arterial disease | ICD 8: 440 441 442 443 444 445  ICD10: I70 I71 I72 I73 I74 I77 |
|  | Ischaemic stroke | ICD 8: 433 434  ICD 10: I63 I64 |
|  | Transient ischaemic attack | ICD 8: 435  ICD10: G45.9 |
| Heart failure |  | ICD8: 427.09; 427.10; 427.11; 427.19; 428.99; 782.49  ICD 10: I50; I11.0; I13.0; I13.2 |
| Renal disease |  | ICD 8: 403; 404; 580–583; 584; 590.09; 593.19; 753.10– 753.19; 792  ICD 10: I12; I13; N00–N05; N07; N11; N14; N17–N19; Q61 |
