# Supplementary material for: Diabetes treatment for persons with severe mental illness: A registry-based cohort study to explore medication treatment differences for persons with type 2 diabetes with and without severe mental illness
Source: PLoS One. 2023 Jun 13;18(6):e0287017. doi: 10.1371/journal.pone.0287017 (PMC10263345; doi:10.1371/journal.pone.0287017)
Supplement: S2 Table — Bold text indicates where the difference is statistically significant at the 0.05 level. RR = rate ratio. ATC (anatomical therapeutic classification) codes are presented in parentheses. DPP4i = dipeptidyl peptidase 4 inhibitors. GLP1-RAs = glucagon-like peptide 1 receptor agonists, SGLT2i = sodium-glucose cotransporter 2 inhibitors. (DOCX) [file pone.0287017.s002.docx]

**S2 Table: Crude and adjusted rate ratios for glucose-lowering medications.**

Bold text indicates where the difference is statistically significant at the 0.05 level. RR= rate ratio. ATC (anatomical therapeutic classification) codes are presented in parentheses. DPP4i = dipeptidyl peptidase 4 inhibitors. GLP1-RAs = glucagon-like peptide 1 receptor agonists, SGLT2i = sodium-glucose cotransporter 2 inhibitors.

| Months after index | Crude RR | Adjusted^a^ RR | Crude RR | | Adjusted^a^ RR |
| --- | --- | --- | --- | --- | --- |
|  | Drugs used in diabetes (A10) | | Insulins and analogues (A10A) | | |
| 0-6  6-12  12-18  18-24  24-30  30-36  36-42  42-48  48-54  54-60  60-66  66-72  72-78  78-84  84-90  90-96  96-102  102-108  108-114  114-120 | 1.05 (0.99-1.12)  **1.10 (1.04-1.17)**  **1.09 (1.03-1.15)**  **1.08 (1.03-1.14)**  1.05 (1.00-1.10)  1.00 (0.95-1.05)  1.00 (0.96-1.05)  0.98 (0.94-1.03)  1.02 (0.97-1.06)  1.00 (0.95-1.05)  0.98 (0.94-1.03)  0.97 (0.92-1.02)  0.97 (0.92-1.02)  0.97 (0.92-1.02)  0.96 (0.91-1.02)  0.97 (0.92-1.03)  0.99 (0.94-1.04)  0.97 (0.92-1.03)  0.99 (0.94-1.05)  1.00 (0.95-1.06) | 1.02 (0.96-1.08)  **1.07 (1.01-1.13)**  **1.06 (1.01-1.12)**  **1.05 (1.00-1.11)**  1.02 (0.97-1.07)  0.98 (0.93-1.03)  0.98 (0.94-1.03)  0.96 (0.92-1.01)  1.00 (0.95-1.04)  0.98 (0.94-1.03)  0.97 (0.92-1.01)  **0.95 (0.90-1.00)**  0.96 (0.91-1.01)  0.95 (0.91-1.01)  0.95 (0.91-1.01)  0.96 (0.91-1.01)  0.98 (0.92-1.03)  0.96 (0.90-1.01)  0.97 (0.92-1.03)  0.98 (0.93-1.04) | **1.45 (1.06-1.99)**  **1.67 (1.25-2.21)**  **1.62 (1.22-2.14)**  **1.55 (1.19-2.01)**  **1.51 (1.18-1.94)**  **1.40 (1.09-1.79)**  **1.48 (1.18-1.87)**  **1.48 (1.18-1.85)**  **1.57 (1.27-1.94)**  **1.52 (1.23-1.88)**  **1.53 (1.25-1.88)**  **1.49 (1.22-1.83)**  **1.44 (1.17-1.77)**  **1.42 (1.16-1.75)**  **1.54 (1.26-1.87)**  **1.57 (1.29-1.91)**  **1.38 (1.12-1.70)**  **1.43 (1.17-1.76)**  **1.49 (1.22-1.81)**  **1.46 (1.19-1.79)** | | 1.22 (0.90-1.67)  **1.40 (1.05-1.85)**  **1.36 (1.03-1.79)**  1.30 (1.00-1.69)  1.27 (0.99-1.63)  1.17 (0.92-1.51)  1.26 (1.00-1.58)  1.25 (1.00-1.57)  **1.34 (1.08-1.65)**  **1.30 (1.05-1.61)**  **1.31 (1.07-1.61)**  **1.26 (1.03-1.55)**  1.23 (1.00-1.51)  1.21 (0.98-1.49)  **1.29 (1.05-1.58)**  **1.29 (1.06-1.58)**  1.13 (0.92-1.40)  1.16 (0.94-1.43)  1.20 (0.98-1.46)  1.17 (0.95-1.44) |
|  | Biguanides (A10BA) | | Sulfonylureas (A10BB) | | |
| 0-6  6-12  12-18  18-24  24-30  30-36  36-42  42-48  48-54  54-60  60-66  66-72  72-78  78-84  84-90  90-96  96-102  102-108  108-114  114-120 | **1.14 (1.06-1.22)**  **1.22 (1.14-1.31)**  **1.21 (1.14-1.29)**  **1.20 (1.13-1.27)**  **1.14 (1.08-1.22)**  **1.11 (1.05-1.18)**  **1.10 (1.04-1.17)**  **1.09 (1.03-1.15)**  **1.12 (1.06-1.18)**  **1.09 (1.03-1.16)**  **1.07 (1.01-1.14)**  1.03 (0.97-1.10)  1.03 (0.96-1.10)  1.01 (0.94-1.08)  1.00 (0.94-1.08)  0.99 (0.92-1.07)  1.06 (0.98-1.14)  1.03 (0.96-1.11)  1.07 (0.99-1.15)  1.05 (0.97-1.14) | **1.08 (1.01-1.16)**  **1.16 (1.08-1.24)**  **1.15 (1.08-1.22)**  **1.13 (1.07-1.20)**  **1.09 (1.02-1.15)**  1.06 (0.99-1.12)  1.05 (0.99-1.11)  1.03 (0.98-1.10)  **1.07 (1.01-1.13)**  1.05 (0.99-1.11)  1.02 (0.96-1.09)  0.99 (0.93-1.06)  0.99 (0.93-1.06)  0.98 (0.91-1.05)  0.98 (0.91-1.05)  0.96 (0.90-1.04)  1.03 (0.96-1.11)  1.00 (0.93-1.08)  1.04 (0.96-1.12)  1.02 (0.94-1.11) | **0.82 (0.69-0.97)**  **0.84 (0.71-0.99)**  **0.81 (0.68-0.96)**  0.89 (0.77-1.04)  **0.83 (0.71-0.97)**  **0.86 (0.74-1.00)**  **0.76 (0.65-0.89)**  **0.74 (0.63-0.87)**  **0.74 (0.63-0.87)**  **0.74 (0.63-0.87)**  **0.80 (0.69-0.94)**  **0.81 (0.69-0.95)**  0.85 (0.72-1.00)  0.91 (0.77-1.07)  0.93 (0.79-1.11)  0.87 (0.72-1.04)  0.87 (0.72-1.05)  0.90 (0.74-1.09)  0.85 (0.68-1.05)  0.84 (0.67-1.05) | | 0.94 (0.80-1.12)  0.97 (0.83-1.15)  0.94 (0.80-1.11)  1.04 (0.90-1.21)  0.97 (0.83-1.13)  0.99 (0.86-1.15)  0.89 (0.76-1.04)  0.87 (0.75-1.02)  0.88 (0.75-1.03)  0.88 (0.75-1.03)  0.95 (0.82-1.11)  0.95 (0.81-1.11)  0.98 (0.83-1.15)  1.03 (0.88-1.21)  1.04 (0.88-1.22)  0.94 (0.79-1.12)  0.93 (0.77-1.11)  0.95 (0.78-1.14)  0.89 (0.72-1.09)  0.87 (0.70-1.09) |
|  | DPP4 inhibitors (A10BH) | | GLP-1RAs (A10BJ) | | |
| 0-6  6-12  12-18  18-24  24-30  30-36  36-42  42-48  48-54  54-60  60-66  66-72  72-78  78-84  84-90  90-96  96-102  102-108  108-114  114-120 | 0.70 (0.35-1.40)  0.89 (0.50-1.58)  0.92 (0.55-1.53)  0.88 (0.54-1.45)  0.98 (0.63-1.50)  0.97 (0.65-1.45)  0.91 (0.62-1.35)  0.79 (0.52-1.19)  1.04 (0.73-1.48)  1.19 (0.86-1.64)  1.27 (0.94-1.72)  1.19 (0.87-1.63)  1.22 (0.89-1.66)  1.13 (0.82-1.56)  1.12 (0.81-1.54)  1.16 (0.85-1.59)  1.28 (0.95-1.71)  1.26 (0.94-1.70)  **1.41 (1.06-1.87)**  1.21 (0.88-1.65) | 0.68 (0.34-1.37)  0.87 (0.49-1.54)  0.89 (0.54-1.47)  0.85 (0.52-1.39)  0.93 (0.60-1.42)  0.91 (0.61-1.36)  0.86 (0.58-1.27)  0.74 (0.49-1.11)  0.97 (0.68-1.38)  1.11 (0.81-1.53)  1.18 (0.87-1.60)  1.10 (0.81-1.51)  1.16 (0.85-1.58)  1.09 (0.79-1.50)  1.09 (0.79-1.50)  1.14 (0.83-1.55)  1.26 (0.94-1.69)  1.24 (0.92-1.68)  **1.37 (1.03-1.83)**  1.18 (0.86-1.61) | **3.09 (1.66-5.74)**  **2.36 (1.37-4.05)**  **1.79 (1.07-3.00)**  **2.02 (1.31-3.13)**  **2.20 (1.51-3.21)**  **1.77 (1.22-2.57)**  **1.60 (1.12-2.30)**  **1.61 (1.15-2.25)**  **1.42 (1.02-1.99)**  **1.52 (1.11-2.08)**  **1.80 (1.36-2.37)**  **1.63 (1.23-2.16)**  **1.67 (1.26-2.20)**  **1.59 (1.21-2.09)**  **1.65 (1.27-2.14)**  **1.61 (1.24-2.09)**  **1.35 (1.02-1.78)**  1.30 (0.98-1.72)  1.23 (0.92-1.63)  1.27 (0.96-1.68) | | **2.33 (1.26-4.31)**  **1.77 (1.04-3.02)**  1.33 (0.80-2.22)  1.49 (0.96-2.29)  **1.60 (1.10-2.33)**  1.28 (0.88-1.85)  1.16 (0.81-1.66)  1.16 (0.84-1.61)  1.02 (0.73-1.42)  1.09 (0.80-1.48)  1.27 (0.97-1.67)  1.16 (0.88-1.52)  1.21 (0.92-1.59)  1.16 (0.88-1.51)  1.20 (0.93-1.56)  1.17 (0.91-1.51)  1.00 (0.76-1.31)  0.96 (0.73-1.26)  0.91 (0.69-1.20)  0.94 (0.71-1.24) |
|  | SGLT-2 inhibitors (A10BK) | | Combinations of glucose-lowering medications (A10BD) | | |
| 0-6  6-12  12-18  18-24  24-30  30-36  36-42  42-48  48-54  54-60  60-66  66-72  72-78  78-84  84-90  90-96  96-102  102-108  108-114  114-120 | No redemptions  0.60 (0.08-4.38)  0.75 (0.18-3.06)  0.94 (0.35-2.55)  1.56 (0.83-2.93)  1.40 (0.79-2.48)  1.24 (0.76-2.03)  1.46 (0.92-2.30)  0.91 (0.53-1.57)  0.99 (0.58-1.67)  **1.57 (1.03-2.39)**  1.17 (0.72-1.88)  1.43 (0.92-2.22)  1.28 (0.82-1.97)  1.29 (0.83-1.99)  1.33 (0.86-2.06)  **1.95 (1.35-2.80)**  **1.65 (1.12-2.43)**  1.21 (0.78-1.88)  1.04 (0.64-1.69) | No redemptions  0.46 (0.06-3.32)  0.58 (0.14-2.37)  0.73 (0.27-1.99)  1.20 (0.64-2.26)  1.08 (0.61-1.92)  0.96 (0.58-1.57)  1.10 (0.70-1.74)  0.68 (0.39-1.17)  0.71 (0.42-1.21)  1.08 (0.71-1.65)  0.80 (0.50-1.29)  1.04 (0.67-1.62)  0.95 (0.62-1.47)  0.94 (0.61-1.45)  0.93 (0.60-1.44)  1.40 (0.98-2.00)  1.14 (0.78-1.67)  0.82 (0.53-1.27)  0.71 (0.44-1.13) | 0.86 (0.45-1.67)  0.61 (0.32-1.18)  0.80 (0.47-1.36)  0.86 (0.55-1.35)  0.91 (0.61-1.36)  0.93 (0.65-1.34)  1.04 (0.76-1.44)  0.90 (0.65-1.25)  0.83 (0.59-1.16)  **0.67 (0.46-0.96)**  **0.68 (0.47-0.98)**  **0.63 (0.43-0.92)**  **0.70 (0.49-1.00)**  0.78 (0.56-1.10)  0.86 (0.62-1.19)  **0.69 (0.48-0.99)**  **0.59 (0.40-0.88)**  **0.65 (0.44-0.94)**  **0.66 (0.46-0.97)**  0.74 (0.51-1.07) | 0.73 (0.38-1.40)  **0.51 (0.27-0.98)**  0.67 (0.40-1.12)  0.72 (0.46-1.12)  0.76 (0.51-1.12)  0.77 (0.54-1.10)  0.87 (0.63-1.19)  0.74 (0.54-1.03)  **0.69 (0.49-0.96)**  **0.55 (0.38-0.80)**  **0.56 (0.39-0.80)**  **0.52 (0.36-0.76)**  **0.59 (0.41-0.85)**  **0.67 (0.48-0.94)**  0.75 (0.54-1.04)  **0.60 (0.42-0.87)**  **0.53 (0.35-0.78)**  **0.57 (0.39-0.83)**  **0.58 (0.40-0.84)**  **0.64 (0.44-0.93)** | |

^a^ Adjusted for: sex, age (quadratic), glycaemic control at the index date, comorbidities preceding the index date (S1 Table), calendar year, and level of education.
