# Supplementary material for: Diabetes treatment for persons with severe mental illness: A registry-based cohort study to explore medication treatment differences for persons with type 2 diabetes with and without severe mental illness
Source: PLoS One. 2023 Jun 13;18(6):e0287017. doi: 10.1371/journal.pone.0287017 (PMC10263345; doi:10.1371/journal.pone.0287017)
Supplement: S3 Table — Bold text indicates where the difference is statistically significant at the 0.05 level. RR = rate ratio. ATC (anatomical therapeutic classification) codes are presented in parentheses. (DOCX) [file pone.0287017.s003.docx]

**S3 Table. Crude and adjusted rate ratios for cardiovascular medications.** Bold text indicates where the difference is statistically significant at the 0.05 level. RR = rate ratio. ATC (anatomical therapeutic classification) codes are presented in parentheses.

| Months after index | Crude RR | Adjusted^a^ RR | Crude RR | Adjusted^a^ RR | |
| --- | --- | --- | --- | --- | --- |
|  | Cardiovascular medications (B01AC, C01, C03, C07, C08, C09, C10) | | Platelet aggregation inhibitors (B01AC) | | |
| 0-6  6-12  12-18  18-24  24-30  30-36  36-42  42-48  48-54  54-60  60-66  66-72  72-78  78-84  84-90  90-96  96-102  102-108  108-114  114-120 | **0.87 (0.84-0.91)**  **0.87 (0.84-0.91)**  **0.89 (0.85-0.93)**  **0.89 (0.85-0.92)**  **0.88 (0.85-0.92)**  **0.88 (0.85-0.92)**  **0.89 (0.86-0.93)**  **0.90 (0.87-0.94)**  **0.90 (0.87-0.94)**  **0.91 (0.88-0.95)**  **0.93 (0.89-0.97)**  **0.93 (0.89-0.97)**  **0.94 (0.90-0.98)**  **0.92 (0.88-0.96)**  **0.92 (0.87-0.96)**  **0.93 (0.89-0.97)**  **0.93 (0.89-0.97)**  **0.94 (0.90-0.99)**  **0.91 (0.86-0.96)**  **0.92 (0.87-0.97)** | **0.94 (0.90-0.98)**  **0.94 (0.90-0.98)**  **0.96 (0.92-1.00)**  **0.96 (0.92-0.99)**  **0.96 (0.92-0.99)**  **0.96 (0.92-0.99)**  0.97 (0.93-1.00)  0.97 (0.94-1.01)  0.98 (0.94-1.02)  0.99 (0.95-1.03)  1.01 (0.97-1.05)  1.01 (0.97-1.05)  1.02 (0.98-1.06)  1.00 (0.95-1.04)  1.00 (0.95-1.04)  1.02 (0.97-1.06)  1.01 (0.97-1.06)  1.03 (0.98-1.08)  0.99 (0.94-1.04)  1.01 (0.95-1.06) | **0.75 (0.67-0.84)**  **0.77 (0.69-0.86)**  **0.71 (0.63-0.79)**  **0.73 (0.65-0.82)**  **0.72 (0.65-0.81)**  **0.74 (0.66-0.82)**  **0.75 (0.68-0.84)**  **0.74 (0.67-0.83)**  **0.76 (0.68-0.85)**  **0.78 (0.70-0.87)**  **0.82 (0.73-0.91)**  **0.80 (0.72-0.90)**  **0.84 (0.75-0.95)**  **0.84 (0.75-0.95)**  **0.86 (0.77-0.97)**  0.89 (0.79-1.01)  0.89 (0.78-1.00)  0.90 (0.79-1.02)  0.88 (0.77-1.00)  0.89 (0.77-1.02) | | 0.91 (0.82-1.00)  0.94 (0.86-1.04)  **0.88 (0.79-0.97)**  **0.90 (0.82-1.00)**  **0.89 (0.81-0.99)**  0.91 (0.82-1.01)  0.93 (0.85-1.03)  0.92 (0.83-1.02)  0.95 (0.86-1.05)  0.98 (0.88-1.09)  1.02 (0.92-1.13)  1.00 (0.90-1.11)  1.04 (0.94-1.16)  1.04 (0.93-1.17)  1.06 (0.94-1.19)  1.09 (0.97-1.23)  1.08 (0.96-1.22)  1.10 (0.97-1.24)  1.07 (0.94-1.21)  1.08 (0.94-1.24) |
|  | Cardiac therapy (C01) | | Diuretics (C03) | | |
| 0-6  6-12  12-18  18-24  24-30  30-36  36-42  42-48  48-54  54-60  60-66  66-72  72-78  78-84  84-90  90-96  96-102  102-108  108-114  114-120 | **0.71 (0.58-0.87)**  **0.80 (0.65-0.98)**  **0.70 (0.56-0.87)**  **0.75 (0.60-0.93)**  **0.64 (0.50-0.81)**  **0.65 (0.51-0.83)**  **0.71 (0.56-0.90)**  **0.75 (0.59-0.95)**  **0.76 (0.59-0.97)**  **0.73 (0.56-0.95)**  **0.76 (0.58-0.99)**  0.78 (0.59-1.03)  **0.62 (0.44-0.86)**  **0.59 (0.42-0.84)**  **0.60 (0.42-0.87)**  0.78 (0.56-1.08)  **0.66 (0.45-0.95)**  0.72 (0.50-1.04)  0.84 (0.59-1.20)  0.76 (0.51-1.12) | 0.92 (0.76-1.11)  1.08 (0.90-1.29)  0.96 (0.79-1.17)  1.04 (0.85-1.27)  0.89 (0.71-1.10)  0.92 (0.74-1.14)  0.99 (0.79-1.22)  1.04 (0.83-1.29)  1.05 (0.83-1.32)  1.04 (0.81-1.33)  1.08 (0.84-1.37)  1.11 (0.87-1.43)  0.90 (0.66-1.22)  0.89 (0.64-1.23)  0.88 (0.63-1.25)  1.15 (0.84-1.57)  0.95 (0.67-1.36)  1.06 (0.75-1.50)  1.21 (0.86-1.70)  1.11 (0.76-1.62) | 0.92 (0.84-1.00)  **0.88 (0.80-0.96)**  **0.87 (0.79-0.96)**  **0.86 (0.78-0.95)**  **0.86 (0.78-0.95)**  **0.79 (0.71-0.89)**  **0.86 (0.77-0.96)**  **0.84 (0.75-0.94)**  **0.84 (0.75-0.94)**  **0.83 (0.74-0.94)**  **0.89 (0.79-1.00)**  **0.84 (0.74-0.95)**  **0.82 (0.71-0.93)**  **0.82 (0.71-0.94)**  **0.83 (0.72-0.96)**  **0.80 (0.68-0.94)**  **0.79 (0.67-0.93)**  **0.76 (0.64-0.90)**  **0.82 (0.70-0.98)**  **0.92 (0.78-1.08)** | | **1.09 (1.00-1.19)**  1.05 (0.96-1.15)  1.05 (0.96-1.16)  1.05 (0.96-1.16)  1.06 (0.96-1.16)  0.98 (0.88-1.08)  1.06 (0.96-1.17)  1.03 (0.93-1.14)  1.03 (0.93-1.15)  1.03 (0.92-1.16)  1.10 (0.98-1.23)  1.04 (0.92-1.17)  1.01 (0.89-1.15)  1.02 (0.90-1.17)  1.04 (0.91-1.19)  1.00 (0.87-1.16)  0.98 (0.85-1.14)  0.95 (0.81-1.11)  1.02 (0.87-1.20)  1.14 (0.98-1.34) |
|  | Beta blocking agents (C07) | | Calcium channel blockers (C08) | | |
| 0-6  6-12  12-18  18-24  24-30  30-36  36-42  42-48  48-54  54-60  60-66  66-72  72-78  78-84  84-90  90-96  96-102  102-108  108-114  114-120 | **0.64 (0.55-0.74)**  **0.66 (0.57-0.76)**  **0.62 (0.54-0.72)**  **0.66 (0.57-0.77)**  **0.64 (0.55-0.74)**  **0.63 (0.54-0.73)**  **0.60 (0.52-0.70)**  **0.63 (0.54-0.74)**  **0.67 (0.58-0.78)**  **0.63 (0.53-0.74)**  **0.59 (0.49-0.70)**  **0.60 (0.50-0.72)**  **0.58 (0.48-0.70)**  **0.56 (0.45-0.68)**  **0.53 (0.43-0.66)**  **0.52 (0.42-0.65)**  **0.56 (0.45-0.70)**  **0.55 (0.44-0.69)**  **0.61 (0.49-0.77)**  **0.57 (0.45-0.73)** | **0.75 (0.65-0.87)**  **0.78 (0.68-0.90)**  **0.75 (0.65-0.87)**  **0.80 (0.70-0.92)**  **0.77 (0.67-0.89)**  **0.76 (0.66-0.88)**  **0.73 (0.63-0.85)**  **0.76 (0.66-0.89)**  **0.81 (0.70-0.94)**  **0.76 (0.65-0.90)**  **0.71 (0.60-0.85)**  **0.73 (0.61-0.87)**  **0.70 (0.58-0.85)**  **0.68 (0.56-0.84)**  **0.65 (0.52-0.80)**  **0.64 (0.52-0.80)**  **0.69 (0.56-0.86)**  **0.68 (0.54-0.86)**  **0.75 (0.60-0.94)**  **0.71 (0.56-0.90)** | **0.67 (0.58-0.78)**  **0.72 (0.62-0.82)**  **0.70 (0.61-0.80)**  **0.67 (0.58-0.78)**  **0.62 (0.53-0.72)**  **0.64 (0.55-0.74)**  **0.63 (0.54-0.73)**  **0.66 (0.57-0.76)**  **0.67 (0.58-0.78)**  **0.63 (0.54-0.74)**  **0.64 (0.54-0.75)**  **0.64 (0.54-0.75)**  **0.59 (0.49-0.70)**  **0.57 (0.48-0.69)**  **0.65 (0.55-0.78)**  **0.61 (0.50-0.74)**  **0.60 (0.50-0.74)**  **0.65 (0.54-0.79)**  **0.63 (0.52-0.78)**  **0.65 (0.53-0.80)** | **0.78 (0.68-0.90)**  **0.84 (0.73-0.96)**  **0.82 (0.71-0.94)**  **0.79 (0.69-0.92)**  **0.73 (0.63-0.84)**  **0.76 (0.65-0.88)**  **0.74 (0.64-0.86)**  **0.78 (0.67-0.90)**  **0.80 (0.69-0.93)**  **0.75 (0.64-0.88)**  **0.76 (0.65-0.89)**  **0.76 (0.65-0.90)**  **0.69 (0.58-0.83)**  **0.68 (0.57-0.82)**  **0.78 (0.66-0.93)**  **0.74 (0.61-0.89)**  **0.73 (0.60-0.88)**  **0.78 (0.65-0.95)**  **0.76 (0.62-0.93)**  **0.79 (0.64-0.97)** | |
|  | Agents acting on the renin-angiotensin system (C09) | | Lipid-modifying agents (C10) | | |
| 0-6  6-12  12-18  18-24  24-30  30-36  36-42  42-48  48-54  54-60  60-66  66-72  72-78  78-84  84-90  90-96  96-102  102-108  108-114  114-120 | **0.63 (0.57-0.69)**  **0.63 (0.57-0.69)**  **0.65 (0.59-0.71)**  **0.65 (0.59-0.71)**  **0.67 (0.61-0.73)**  **0.65 (0.59-0.71)**  **0.64 (0.59-0.70)**  **0.66 (0.60-0.71)**  **0.67 (0.61-0.73)**  **0.64 (0.58-0.70)**  **0.64 (0.58-0.70)**  **0.66 (0.60-0.73)**  **0.66 (0.60-0.73)**  **0.68 (0.62-0.76)**  **0.70 (0.64-0.78)**  **0.71 (0.64-0.78)**  **0.72 (0.65-0.80)**  **0.72 (0.65-0.80)**  **0.70 (0.63-0.79)**  **0.71 (0.63-0.80)** | **0.69 (0.63-0.75)**  **0.69 (0.63-0.75)**  **0.71 (0.65-0.78)**  **0.72 (0.66-0.78)**  **0.73 (0.67-0.80)**  **0.71 (0.65-0.78)**  **0.71 (0.65-0.77)**  **0.72 (0.66-0.79)**  **0.74 (0.68-0.81)**  **0.71 (0.65-0.78)**  **0.71 (0.65-0.78)**  **0.74 (0.67-0.81)**  **0.74 (0.67-0.81)**  **0.76 (0.69-0.84)**  **0.79 (0.71-0.87)**  **0.80 (0.72-0.88)**  **0.81 (0.73-0.90)**  **0.81 (0.73-0.90)**  **0.79 (0.70-0.88)**  **0.79 (0.71-0.89)** | **0.88 (0.81-0.94)**  **0.88 (0.81-0.94)**  **0.90 (0.84-0.97)**  **0.89 (0.83-0.95)**  **0.87 (0.81-0.93)**  **0.88 (0.82-0.94)**  **0.86 (0.81-0.92)**  **0.88 (0.83-0.94)**  **0.90 (0.84-0.96)**  **0.93 (0.87-0.99)**  0.94 (0.88-1.01)  **0.91 (0.85-0.98)**  **0.93 (0.87-1.00)**  0.93 (0.87-1.00)  **0.92 (0.85-0.99)**  0.93 (0.86-1.00)  **0.90 (0.83-0.98)**  0.94 (0.87-1.02)  **0.92 (0.84-1.00)**  **0.91 (0.83-0.99)** | **0.91 (0.85-0.98)**  **0.91 (0.85-0.98)**  0.94 (0.88-1.01)  **0.93 (0.87-1.00)**  **0.91 (0.85-0.97)**  **0.92 (0.86-0.99)**  **0.91 (0.85-0.97)**  **0.93 (0.87-0.99)**  0.94 (0.88-1.01)  0.98 (0.92-1.04)  0.99 (0.93-1.06)  0.96 (0.90-1.03)  0.98 (0.91-1.05)  0.98 (0.91-1.06)  0.97 (0.90-1.05)  0.99 (0.91-1.07)  0.96 (0.89-1.05)  1.01 (0.93-1.09)  0.98 (0.90-1.06)  0.97 (0.89-1.06) | |

^a^ Adjusted for sex, age (quadratic), glycaemic control at the index date, comorbidities preceding the index date (S1 Table), calendar year, and level of education.
