# Supplementary material for: Diabetes treatment for persons with severe mental illness: A registry-based cohort study to explore medication treatment differences for persons with type 2 diabetes with and without severe mental illness
Source: PLoS One. 2023 Jun 13;18(6):e0287017. doi: 10.1371/journal.pone.0287017 (PMC10263345; doi:10.1371/journal.pone.0287017)

**S1 Fig. Crude fractions of persons who have redeemed one or more prescriptions of a glucose-lowering medication within a period of 6 months.** Each figure shows the fraction of persons with severe mental illness (SMI) and persons without severe mental illness (non-SMI), with a follow-up of 10 years after diabetes diagnosis divided into six-month periods. ATC (anatomical therapeutic classification) codes can be seen in the manuscript. DPP4-inhibitors = dipeptidyl peptidase 4 inhibitors. GLP1-RAs = glucagon-like peptide 1 receptor agonists, SGLT2-inhibitors = sodium-glucose cotransporter 2 inhibitors.


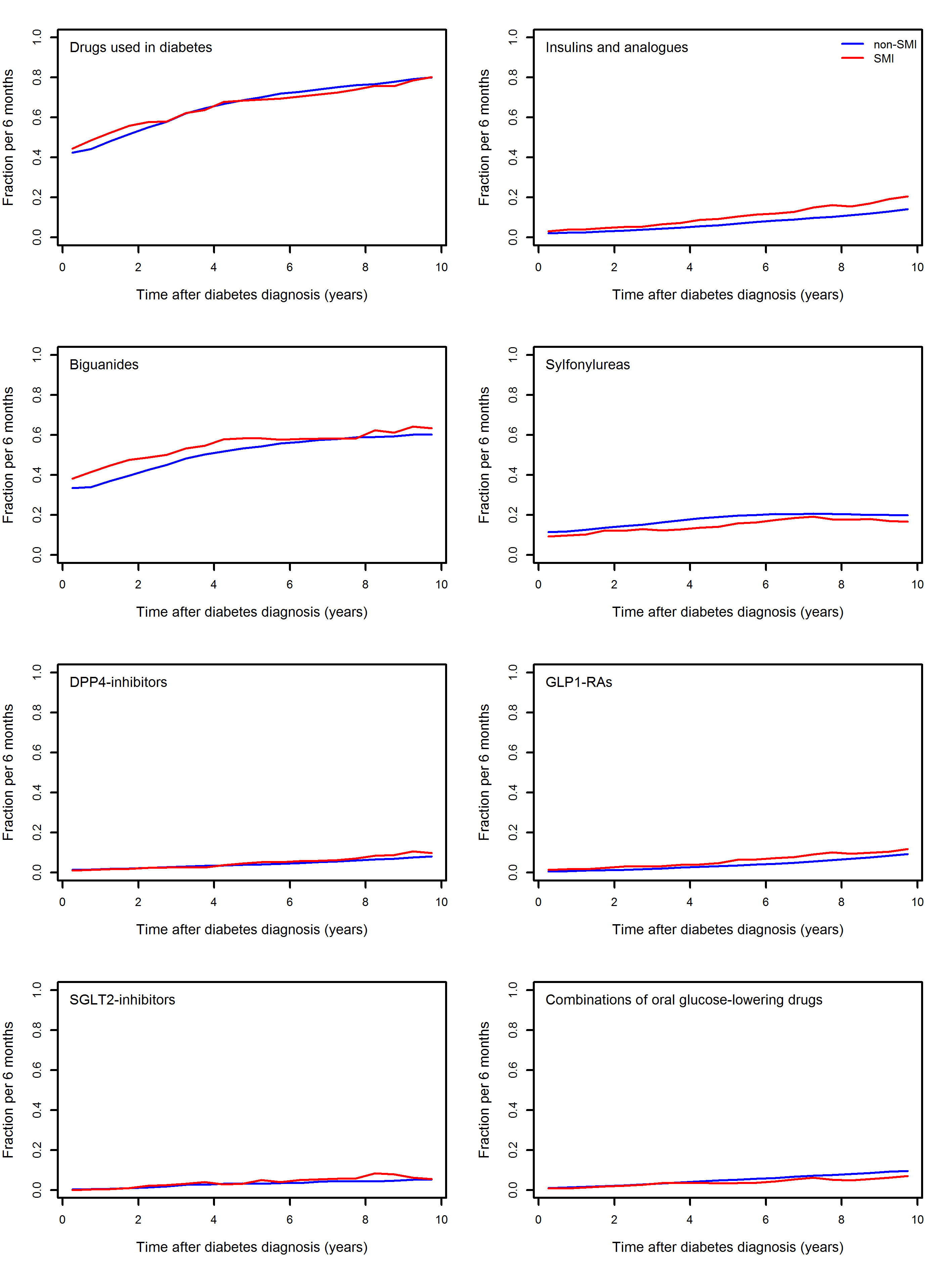

Supplement: S1 Fig — Each figure shows the fraction of persons with severe mental illness (SMI) versus persons without severe mental illness (non-SMI), with a follow-up of 10 years after diabetes diagnosis divided into six-months periods. ATC (anatomical therapeutic classification) codes can be seen in the manuscript. DPP4-inhibitors = dipeptidyl peptidase 4 inhibitors. GLP1-RAs = glucagon-like peptide 1 receptor agonists, SGLT2-inhibitors = sodium-glucose cotransporter 2 inhibitors. (DOCX) [file pone.0287017.s004.docx]
