# Supplementary material for: Diabetes treatment for persons with severe mental illness: A registry-based cohort study to explore medication treatment differences for persons with type 2 diabetes with and without severe mental illness
Source: PLoS One. 2023 Jun 13;18(6):e0287017. doi: 10.1371/journal.pone.0287017 (PMC10263345; doi:10.1371/journal.pone.0287017)

**S2 Fig. Crude fractions of persons who have redeemed one or more prescriptions of a cardiovascular medication within a period of 6 months.** Each figure shows the fractions of patients with severe mental illness (SMI) and patients without severe mental illness (non-SMI) with a follow-up of 10 years after diabetes diagnosis divided into six-month periods. ATC (anatomical therapeutic classification) codes are presented in the manuscript.


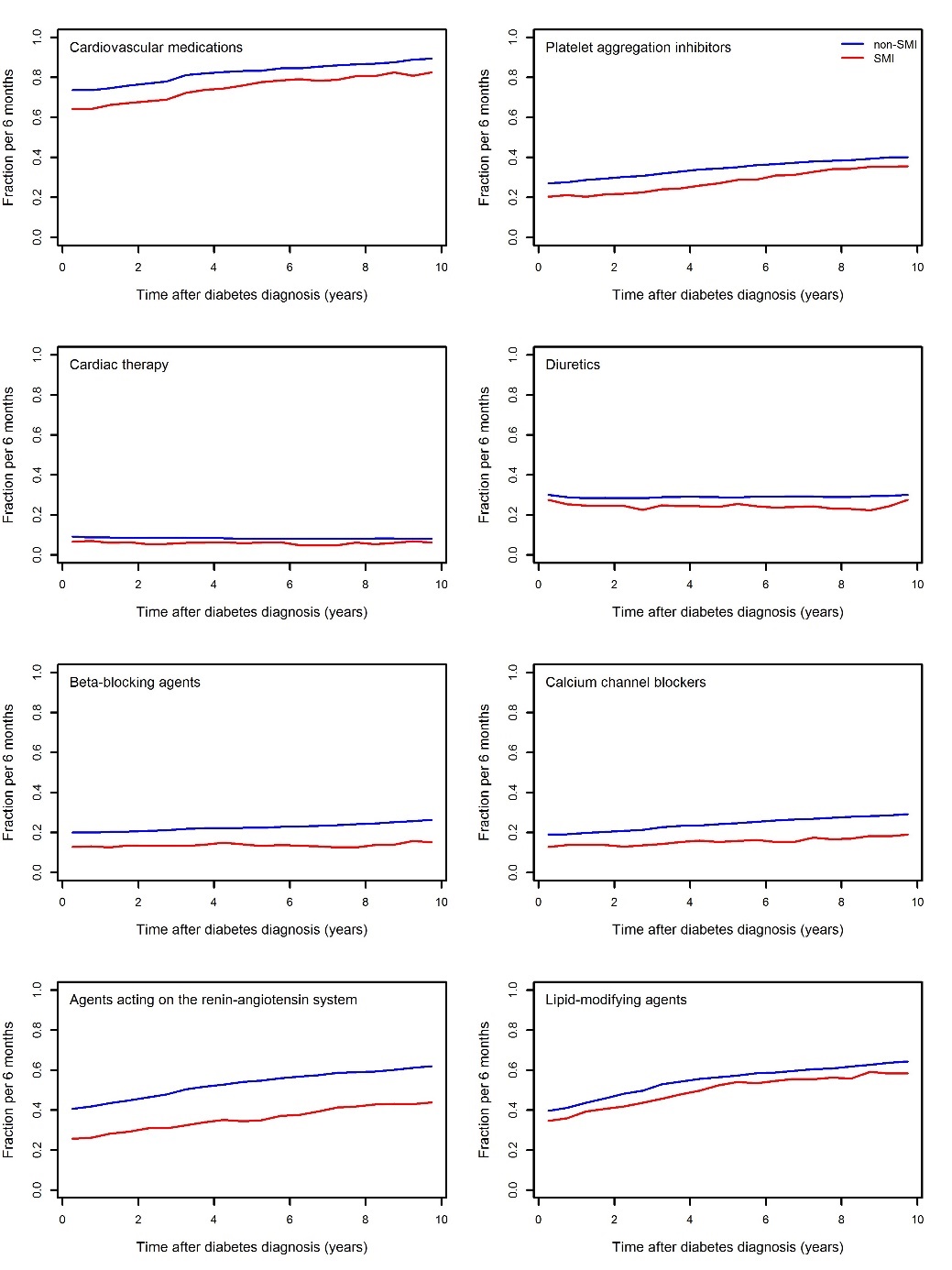

Supplement: S2 Fig — Each figure shows the fractions of patients with severe mental illness (SMI) versus patients without severe mental illness (non-SMI) with a follow-up of 10 years after diabetes diagnosis divided into six-months periods. ATC (anatomical therapeutic classification) codes are presented in the manuscript. (DOCX) [file pone.0287017.s005.docx]
