# Supplementary figures and images for: Diabetes treatment for persons with severe mental illness: A registry-based cohort study to explore medication treatment differences for persons with type 2 diabetes with and without severe mental illness
Source: PLoS One. 2023 Jun 13;18(6):e0287017. doi: 10.1371/journal.pone.0287017 (PMC10263345; doi:10.1371/journal.pone.0287017)

**S3 Fig: Crude fraction of glucose-lowering medication use stratified for SMI-subgroups.**


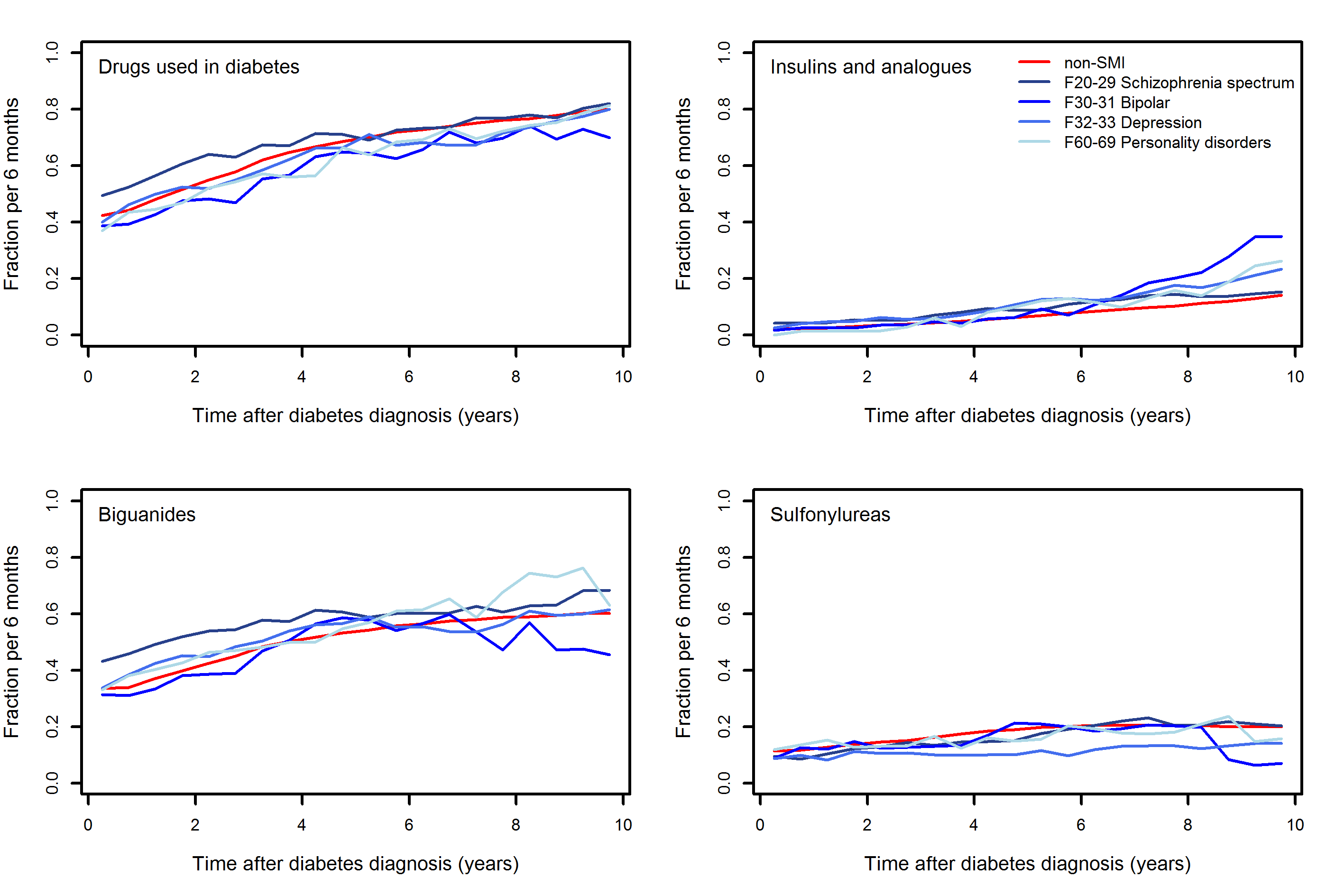

Supplement: S3 Fig — (DOCX) [file pone.0287017.s006.docx]

**S4 Fig: Crude fraction of cardiovascular medication use stratified for SMI-subgroups.**


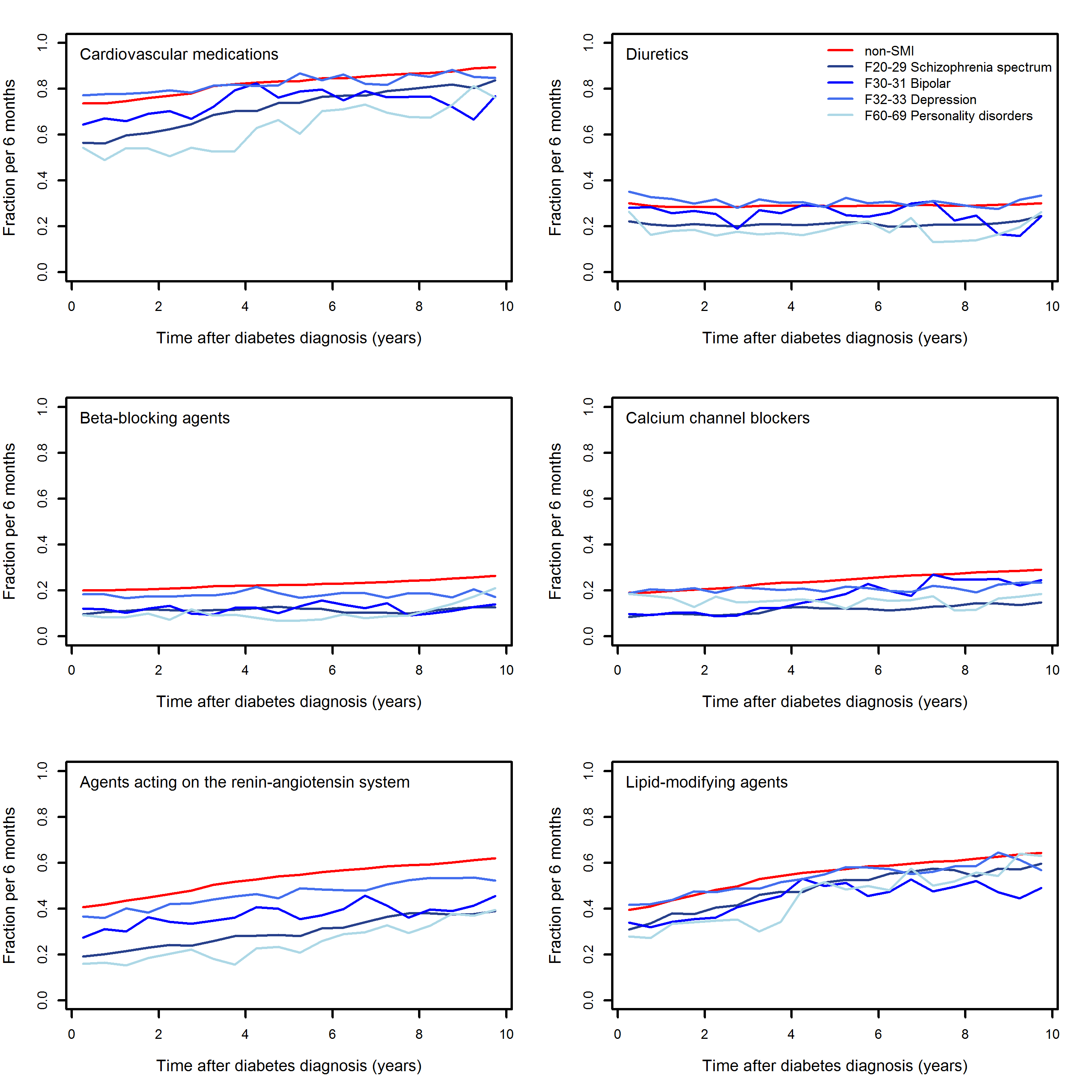

Supplement: S4 Fig — (DOCX) [file pone.0287017.s007.docx]

**S5 Fig. Adjusted rate ratios for glucose-lowering medications stratified for SMI subgroups.**


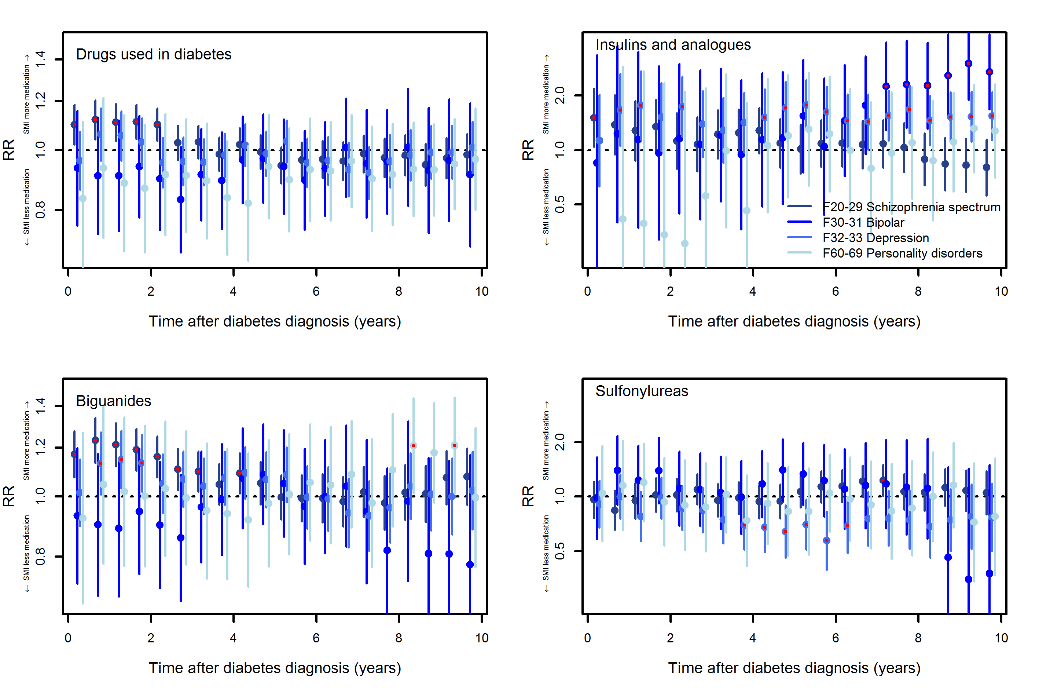

Supplement: S5 Fig — (DOCX) [file pone.0287017.s008.docx]

**Fig S6. Adjusted rate ratios for cardiovascular medications stratified for SMI subgroups.**


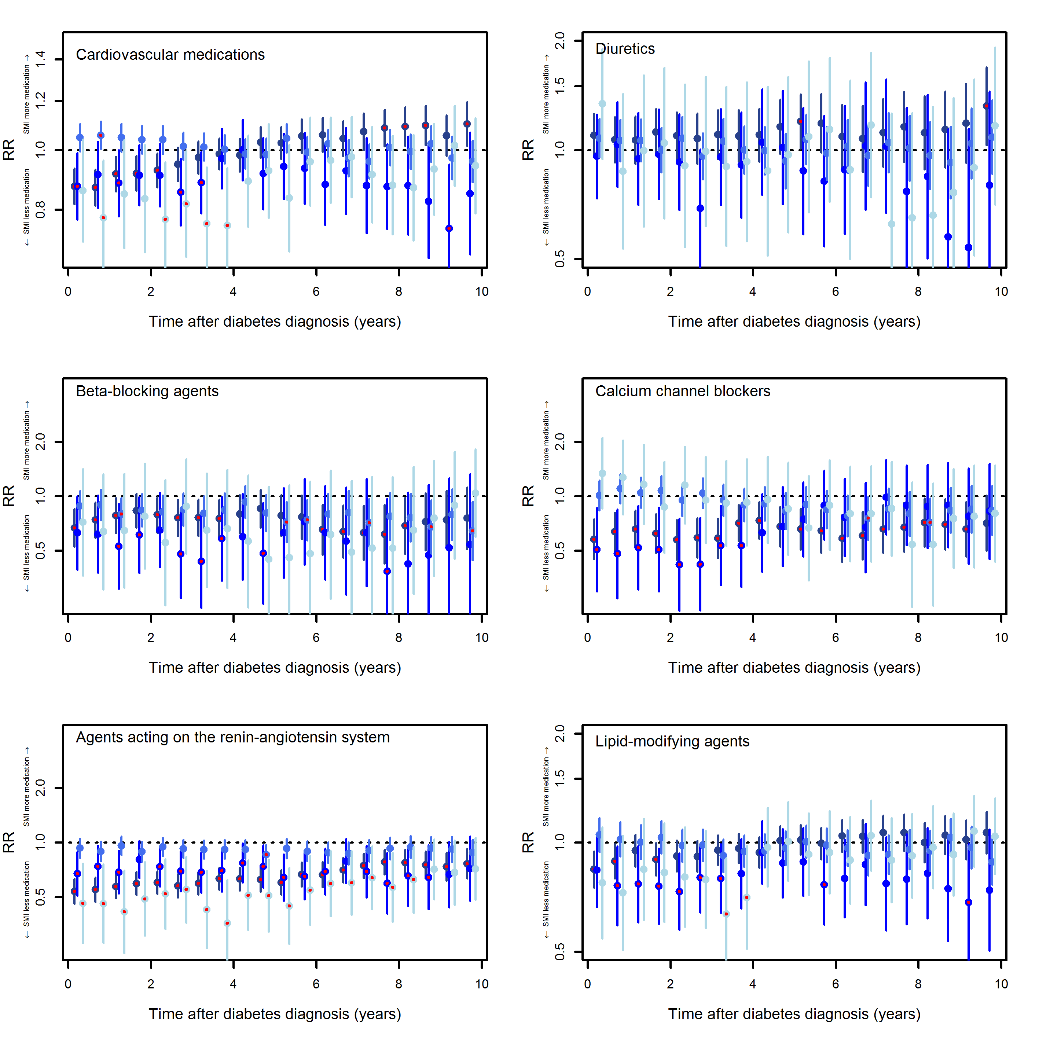

Supplement: S6 Fig — (DOCX) [file pone.0287017.s009.docx]
