# Supplementary material for: Diabetes treatment for persons with severe mental illness: A registry-based cohort study to explore medication treatment differences for persons with type 2 diabetes with and without severe mental illness
Source: PLoS One. 2023 Jun 13;18(6):e0287017. doi: 10.1371/journal.pone.0287017 (PMC10263345; doi:10.1371/journal.pone.0287017)
Supplement: Attachment — Submitted filename: Response to Reviewers.docx [file pone.0287017.s010.docx]

Dear Dr. Bakkedal,

Thank you for submitting your manuscript to PLOS ONE. After careful consideration, we feel that it has merit but does not fully meet PLOS ONE’s publication criteria as it currently stands. Therefore, we invite you to submit a revised version of the manuscript that addresses the points raised during the review process.

==============================

Three reviewers thoroughly read your paper and raised important concerns, particularly in perspective of methodology. As some of the reviewers' comments (e.g., diagnostic criteria and information on prescribed psychotropic medications) do not seem to be resolved by rewriting the paper, please note that this invitation does not guarantee final acceptance of this paper.

==============================

Please submit your revised manuscript by Dec 26 2022 11:59PM. If you will need more time than this to complete your revisions, please reply to this message or contact the journal office at [plosone@plos.org](mailto:plosone@plos.org). When you're ready to submit your revision, log on to [https://www.editorialmanager.com/pone/](https://eur02.safelinks.protection.outlook.com/?url=https%3A%2F%2Fwww.editorialmanager.com%2Fpone%2F&data=05%7C01%7Cbakkedal%40sund.ku.dk%7Cc6f6465557ec4da9f96e08dac41f2bc9%7Ca3927f91cda14696af898c9f1ceffa91%7C0%7C0%7C638037935582375673%7CUnknown%7CTWFpbGZsb3d8eyJWIjoiMC4wLjAwMDAiLCJQIjoiV2luMzIiLCJBTiI6Ik1haWwiLCJXVCI6Mn0%3D%7C3000%7C%7C%7C&sdata=SsG0rRIHnae%2Ffi33HDL1%2F5J0ZlLtZUUmEjcZ0xOiODw%3D&reserved=0) and select the 'Submissions Needing Revision' folder to locate your manuscript file.

Please include the following items when submitting your revised manuscript:

- A rebuttal letter that responds to each point raised by the academic editor and reviewer(s). You should upload this letter as a separate file labeled 'Response to Reviewers'.
- A marked-up copy of your manuscript that highlights changes made to the original version. You should upload this as a separate file labeled 'Revised Manuscript with Track Changes'.
- An unmarked version of your revised paper without tracked changes. You should upload this as a separate file labeled 'Manuscript'.

If you would like to make changes to your financial disclosure, please include your updated statement in your cover letter. Guidelines for resubmitting your figure files are available below the reviewer comments at the end of this letter.

If applicable, we recommend that you deposit your laboratory protocols in [protocols.io](https://eur02.safelinks.protection.outlook.com/?url=http%3A%2F%2Fprotocols.io%2F&data=05%7C01%7Cbakkedal%40sund.ku.dk%7Cc6f6465557ec4da9f96e08dac41f2bc9%7Ca3927f91cda14696af898c9f1ceffa91%7C0%7C0%7C638037935582375673%7CUnknown%7CTWFpbGZsb3d8eyJWIjoiMC4wLjAwMDAiLCJQIjoiV2luMzIiLCJBTiI6Ik1haWwiLCJXVCI6Mn0%3D%7C3000%7C%7C%7C&sdata=cqvtZrFPsBA2ixq1o18Zh69fzO8%2FCNTKRzyPh1bIUX4%3D&reserved=0) to enhance the reproducibility of your results. Protocols.io assigns your protocol its own identifier (DOI) so that it can be cited independently in the future. For instructions see: [https://journals.plos.org/plosone/s/submission-guidelines#loc-laboratory-protocols](https://eur02.safelinks.protection.outlook.com/?url=https%3A%2F%2Fjournals.plos.org%2Fplosone%2Fs%2Fsubmission-guidelines%23loc-laboratory-protocols&data=05%7C01%7Cbakkedal%40sund.ku.dk%7Cc6f6465557ec4da9f96e08dac41f2bc9%7Ca3927f91cda14696af898c9f1ceffa91%7C0%7C0%7C638037935582375673%7CUnknown%7CTWFpbGZsb3d8eyJWIjoiMC4wLjAwMDAiLCJQIjoiV2luMzIiLCJBTiI6Ik1haWwiLCJXVCI6Mn0%3D%7C3000%7C%7C%7C&sdata=Ui9X2bJdmxNurEl%2Fv1SsEuwz856Lv%2FEyn53ZH7KNLS8%3D&reserved=0). Additionally, PLOS ONE offers an option for publishing peer-reviewed Lab Protocol articles, which describe protocols hosted on [protocols.io](https://eur02.safelinks.protection.outlook.com/?url=http%3A%2F%2Fprotocols.io%2F&data=05%7C01%7Cbakkedal%40sund.ku.dk%7Cc6f6465557ec4da9f96e08dac41f2bc9%7Ca3927f91cda14696af898c9f1ceffa91%7C0%7C0%7C638037935582531897%7CUnknown%7CTWFpbGZsb3d8eyJWIjoiMC4wLjAwMDAiLCJQIjoiV2luMzIiLCJBTiI6Ik1haWwiLCJXVCI6Mn0%3D%7C3000%7C%7C%7C&sdata=3l7KLVNB%2FfctlKc1X4EYPKIM9L0mSDgQYvCJytlGSvc%3D&reserved=0). Read more information on sharing protocols at [https://plos.org/protocols?utm_medium=editorial-email&utm_source=authorletters&utm_campaign=protocols](https://eur02.safelinks.protection.outlook.com/?url=https%3A%2F%2Fplos.org%2Fprotocols%3Futm_medium%3Deditorial-email%26utm_source%3Dauthorletters%26utm_campaign%3Dprotocols&data=05%7C01%7Cbakkedal%40sund.ku.dk%7Cc6f6465557ec4da9f96e08dac41f2bc9%7Ca3927f91cda14696af898c9f1ceffa91%7C0%7C0%7C638037935582531897%7CUnknown%7CTWFpbGZsb3d8eyJWIjoiMC4wLjAwMDAiLCJQIjoiV2luMzIiLCJBTiI6Ik1haWwiLCJXVCI6Mn0%3D%7C3000%7C%7C%7C&sdata=Bd9DDwx2YOqcNXGVx%2FqB%2FRwGLScK9bpwjfpYYPIZUvU%3D&reserved=0).

We look forward to receiving your revised manuscript.

Kind regards,

Kyoung-Sae Na, M.D., Ph.D.
Academic Editor
PLOS ONE

Journal Requirements:

When submitting your revision, we need you to address these additional requirements.

1. Please ensure that your manuscript meets PLOS ONE's style requirements, including those for file naming. The PLOS ONE style templates can be found at

[https://journals.plos.org/plosone/s/file?id=wjVg/PLOSOne_formatting_sample_main_body.pdf](https://eur02.safelinks.protection.outlook.com/?url=https%3A%2F%2Fjournals.plos.org%2Fplosone%2Fs%2Ffile%3Fid%3DwjVg%2FPLOSOne_formatting_sample_main_body.pdf&data=05%7C01%7Cbakkedal%40sund.ku.dk%7Cc6f6465557ec4da9f96e08dac41f2bc9%7Ca3927f91cda14696af898c9f1ceffa91%7C0%7C0%7C638037935582531897%7CUnknown%7CTWFpbGZsb3d8eyJWIjoiMC4wLjAwMDAiLCJQIjoiV2luMzIiLCJBTiI6Ik1haWwiLCJXVCI6Mn0%3D%7C3000%7C%7C%7C&sdata=SBSgCrVrM1s%2BsA36mvXAghpa0s3Ks4qNPnYW5nM1ALU%3D&reserved=0) and

[https://journals.plos.org/plosone/s/file?id=ba62/PLOSOne_formatting_sample_title_authors_affiliations.pdf](https://eur02.safelinks.protection.outlook.com/?url=https%3A%2F%2Fjournals.plos.org%2Fplosone%2Fs%2Ffile%3Fid%3Dba62%2FPLOSOne_formatting_sample_title_authors_affiliations.pdf&data=05%7C01%7Cbakkedal%40sund.ku.dk%7Cc6f6465557ec4da9f96e08dac41f2bc9%7Ca3927f91cda14696af898c9f1ceffa91%7C0%7C0%7C638037935582531897%7CUnknown%7CTWFpbGZsb3d8eyJWIjoiMC4wLjAwMDAiLCJQIjoiV2luMzIiLCJBTiI6Ik1haWwiLCJXVCI6Mn0%3D%7C3000%7C%7C%7C&sdata=M34rTSeN3Gg7pC7%2B1h5e%2BCI9VGOWp3bv%2B%2FHM4UOiDYs%3D&reserved=0).

2. You indicated that ethical approval was not necessary for your study. We understand that the framework for ethical oversight requirements for studies of this type may differ depending on the setting and we would appreciate some further clarification regarding your research. Could you please provide further details on why your study is exempt from the need for approval and confirmation from your institutional review board or research ethics committee (e.g., in the form of a letter or email correspondence) that ethics review was not necessary for this study? Please include a copy of the correspondence as an ""Other"" file."

\# The study was approved by the Records of Data Processing activities at University of Copenhagen (514-0361/19-3000), as required by the General Data Protection Regulation, and data access was agreed by Statistics Denmark and the Danish Health Data Authority (FSEID-00004891/DST-project number 707726).

Approval by the Ethics Committee and written informed consent are not required for register-based projects, cf. LBK nr 1338 af 01/09/2020, §10 Bekendtgørelse af lov om videnskabsetisk behandling af sundhedsvidenskabelige forskningsprojekter og sundhedsdatavidenskabelige forskningsprojekter [Act no. 1338 of 1 September 2020, section 10 on research ethics for administration of health scientific research projects and health data scientific research projects]. All results are presented at aggregate level following guidelines from Statistics Denmark.

3.Please provide additional details regarding participant consent. In the ethics statement in the Methods and online submission information, please ensure that you have specified (1) whether consent was informed and (2) what type you obtained (for instance, written or verbal, and if verbal, how it was documented and witnessed). If your study included minors, state whether you obtained consent from parents or guardians. If the need for consent was waived by the ethics committee, please include this information.

If you are reporting a retrospective study of medical records or archived samples, please ensure that you have discussed whether all data were fully anonymized before you accessed them and/or whether the IRB or ethics committee waived the requirement for informed consent. If patients provided informed written consent to have data from their medical records used in research, please include this information.

\# Written informed consent is not required for register-based projects, cf. LBK nr 1338 af 01/09/2020, §10 Bekendtgørelse af lov om videnskabsetisk behandling af sundhedsvidenskabelige forskningsprojekter og sundhedsdatavidenskabelige forskningsprojekter [Act no. 1338 of 1 September 2020, section 10 on research ethics for administration of health scientific research projects and health data scientific research projects].

We changed our phrasings in the manuscript (Ethic section):

*“As required by the General Data Protection Regulation, the Records of Data processing activities at the University of Copenhagen approved the study (514-0361/19-3000), and data access was allowed by Statistics Denmark and the Danish Health Data Authority (FSEID-00004891/DST-project number 707726). For registry-based studies, approval by the Ethics Committee and written informed consent are not necessarily due to Danish legislation. All results are presented at the aggregate level following guidelines from Statistics Denmark”*

4. Thank you for stating in your Funding Statement:

“CB received funding from CACHET (Copenhagen Center for Health Technology, 500110) and the SOFIA project (supported by the Novo Nordisk Foundation, NNF16OC0022038). The funders had no role in study design, data collection and analysis, decision to publish, or preparation of the manuscript. [https://www.cachet.dk/](https://eur02.safelinks.protection.outlook.com/?url=https%3A%2F%2Fwww.cachet.dk%2F&data=05%7C01%7Cbakkedal%40sund.ku.dk%7Cc6f6465557ec4da9f96e08dac41f2bc9%7Ca3927f91cda14696af898c9f1ceffa91%7C0%7C0%7C638037935582531897%7CUnknown%7CTWFpbGZsb3d8eyJWIjoiMC4wLjAwMDAiLCJQIjoiV2luMzIiLCJBTiI6Ik1haWwiLCJXVCI6Mn0%3D%7C3000%7C%7C%7C&sdata=pqx2%2Ffxjo4gRrW3ZAGbssysGYm%2FSWEZhCe9PpsT2aZs%3D&reserved=0)
[https://ifsv.ku.dk/om](https://eur02.safelinks.protection.outlook.com/?url=https%3A%2F%2Fifsv.ku.dk%2Fom&data=05%7C01%7Cbakkedal%40sund.ku.dk%7Cc6f6465557ec4da9f96e08dac41f2bc9%7Ca3927f91cda14696af898c9f1ceffa91%7C0%7C0%7C638037935582531897%7CUnknown%7CTWFpbGZsb3d8eyJWIjoiMC4wLjAwMDAiLCJQIjoiV2luMzIiLCJBTiI6Ik1haWwiLCJXVCI6Mn0%3D%7C3000%7C%7C%7C&sdata=3ofrscUyAHIFt8iL0yG6%2BllFEYckwqr5rETHK4ql7yM%3D&reserved=0) instituttet/almen_medicin/forskning/sofia/”

Please provide an amended statement that declares *all* the funding or sources of support (whether external or internal to your organization) received during this study, as detailed online in our guide for authors at [http://journals.plos.org/plosone/s/submit-now](https://eur02.safelinks.protection.outlook.com/?url=http%3A%2F%2Fjournals.plos.org%2Fplosone%2Fs%2Fsubmit-now&data=05%7C01%7Cbakkedal%40sund.ku.dk%7Cc6f6465557ec4da9f96e08dac41f2bc9%7Ca3927f91cda14696af898c9f1ceffa91%7C0%7C0%7C638037935582531897%7CUnknown%7CTWFpbGZsb3d8eyJWIjoiMC4wLjAwMDAiLCJQIjoiV2luMzIiLCJBTiI6Ik1haWwiLCJXVCI6Mn0%3D%7C3000%7C%7C%7C&sdata=1s7Phlp1sJ7jPrLrk44CTwPEHwckHgxytY8dUOF1QpE%3D&reserved=0).  Please also include the statement “There was no additional external funding received for this study.” in your updated Funding Statement.

Please include your amended Funding Statement within your cover letter. We will change the online submission form on your behalf.

\# Added: *“There was no additional external funding received for this study”*

5. Thank you for stating the following in the Competing Interests section:

‘I have read the journal's policy and the authors of this manuscript have the following competing interests: FP has served as a consultant, on advisory boards or as educator for AstraZeneca, Novo Nordisk, Boehringer Ingelheim, Sanofi, Mundipharma, MSD, Novartis, Amgen and has received research grants to institution from Novo Nordisk, Boehringer Ingelheim, Amgen and AstraZeneca.
CB has been employed as part of her specialization in Clinical Pharmacology in Novo Nordisk in a 1-year position that ended in 2018”

We note that one or more of the authors are employed by a commercial company: AstraZeneca, Novo Nordisk, Boehringer Ingelheim, Sanofi, Mundipharma, MSD, Novartis, Amgen

a.            Please provide an amended Funding Statement declaring this commercial affiliation, as well as a statement regarding the Role of Funders in your study. If the funding organization did not play a role in the study design, data collection and analysis, decision to publish, or preparation of the manuscript and only provided financial support in the form of authors' salaries and/or research materials, please review your statements relating to the author contributions, and ensure you have specifically and accurately indicated the role(s) that these authors had in your study. You can update author roles in the Author Contributions section of the online submission form.

Please also include the following statement within your amended Funding Statement.

“The funder provided support in the form of salaries for authors [insert relevant initials], but did not have any additional role in the study design, data collection and analysis, decision to publish, or preparation of the manuscript. The specific roles of these authors are articulated in the ‘author contributions’ section.”

If your commercial affiliation did play a role in your study, please state and explain this role within your updated Funding Statement.

\# The previous version of the Funding Statement has been updated:

Previous:

CB received funding from CACHET (Copenhagen Center for Health Technology, 500110) and the SOFIA project (supported by the Novo Nordisk Foundation, NNF16OC0022038). The funders had no role in study design, data collection and analysis, decision to publish, or preparation of the manuscript. https://www.cachet.dk/ [https://ifsv.ku.dk/om instituttet/almen_medicin/forskning/sofia/](https://ifsv.ku.dk/om%20instituttet/almen_medicin/forskning/sofia/)

Updated:

CB received funding from CACHET (Copenhagen Center for Health Technology, 500110) and the SOFIA project (supported by the Novo Nordisk Foundation, NNF16OC0022038). The funder provided support in the form of salaries for CB but did not have any additional role in the study design, data collection and analysis, decision to publish, or preparation of the manuscript. The specific roles of these authors are articulated in the ‘author contributions’ section. There was no additional external funding received for this study”

https://www.cachet.dk/ [https://ifsv.ku.dk/om instituttet/almen_medicin/forskning/sofia/](https://ifsv.ku.dk/om%20instituttet/almen_medicin/forskning/sofia/)

b. Please also provide an updated Competing Interests Statement declaring this commercial affiliation along with any other relevant declarations relating to employment, consultancy, patents, products in development, or marketed products, etc.

Within your Competing Interests Statement, please confirm that this commercial affiliation does not alter your adherence to all PLOS ONE policies on sharing data and materials by including the following statement: "This does not alter our adherence to  PLOS ONE policies on sharing data and materials.” (as detailed online in our guide for authors [http://journals.plos.org/plosone/s/competing-interests](https://eur02.safelinks.protection.outlook.com/?url=http%3A%2F%2Fjournals.plos.org%2Fplosone%2Fs%2Fcompeting-interests&data=05%7C01%7Cbakkedal%40sund.ku.dk%7Cc6f6465557ec4da9f96e08dac41f2bc9%7Ca3927f91cda14696af898c9f1ceffa91%7C0%7C0%7C638037935582531897%7CUnknown%7CTWFpbGZsb3d8eyJWIjoiMC4wLjAwMDAiLCJQIjoiV2luMzIiLCJBTiI6Ik1haWwiLCJXVCI6Mn0%3D%7C3000%7C%7C%7C&sdata=UrUC%2Fg6UYaAdVbZMvXeyyI%2BLDXSGatMhTeZ3hJQWIHI%3D&reserved=0)) . If this adherence statement is not accurate and there are restrictions on sharing of data and/or materials, please state these. Please note that we cannot proceed with consideration of your article until this information has been declared.

\# The Competing interest Statement is updated into:

‘I have read the journal's policy and the authors of this manuscript have the following competing interests: CB has been employed as part of her specialization in Clinical Pharmacology in Novo Nordisk in a 1-year position that ended in 2018. FP has served as a consultant, on advisory boards or as educator for AstraZeneca, Novo Nordisk, Boehringer Ingelheim, Sanofi, Mundipharma, MSD, Novartis, Amgen and has received research grants to institution from Novo Nordisk, Boehringer Ingelheim, Amgen and AstraZeneca. This does not alter our adherence to PLOS ONE policies on sharing data and materials”

Please include both an updated Funding Statement and Competing Interests Statement in your cover letter. We will change the online submission form on your behalf.

6. We note that you have included the phrase “data not shown” in your manuscript. Unfortunately, this does not meet our data sharing requirements. PLOS does not permit references to inaccessible data. We require that authors provide all relevant data within the paper, Supporting Information files, or in an acceptable, public repository. Please add a citation to support this phrase or upload the data that corresponds with these findings to a stable repository (such as Figshare or Dryad) and provide and URLs, DOIs, or accession numbers that may be used to access these data. Or, if the data are not a core part of the research being presented in your study, we ask that you remove the phrase that refers to these data.

\# We have removed the phrase: “data not shown” from the manuscript. The meaning of this sentence was solely that the data was not shown in table 1.

[Note: HTML markup is below. Please do not edit.]

Reviewers' comments:

Reviewer's Responses to Questions

**Comments to the Author**

1. Is the manuscript technically sound, and do the data support the conclusions?

The manuscript must describe a technically sound piece of scientific research with data that supports the conclusions. Experiments must have been conducted rigorously, with appropriate controls, replication, and sample sizes. The conclusions must be drawn appropriately based on the data presented.

Reviewer #1: No

Reviewer #2: No

Reviewer #3: Partly

2. Has the statistical analysis been performed appropriately and rigorously?

Reviewer #1: Yes

Reviewer #2: Yes

Reviewer #3: Yes

3. Have the authors made all data underlying the findings in their manuscript fully available?

The [PLOS Data policy](https://eur02.safelinks.protection.outlook.com/?url=http%3A%2F%2Fwww.plosone.org%2Fstatic%2Fpolicies.action%23sharing&data=05%7C01%7Cbakkedal%40sund.ku.dk%7Cc6f6465557ec4da9f96e08dac41f2bc9%7Ca3927f91cda14696af898c9f1ceffa91%7C0%7C0%7C638037935582531897%7CUnknown%7CTWFpbGZsb3d8eyJWIjoiMC4wLjAwMDAiLCJQIjoiV2luMzIiLCJBTiI6Ik1haWwiLCJXVCI6Mn0%3D%7C3000%7C%7C%7C&sdata=GT2dBcbBo6EnoZEe4S8FAOxdbtpru%2FLsA9Y%2FLA3Xn2M%3D&reserved=0) requires authors to make all data underlying the findings described in their manuscript fully available without restriction, with rare exception (please refer to the Data Availability Statement in the manuscript PDF file). The data should be provided as part of the manuscript or its supporting information, or deposited to a public repository. For example, in addition to summary statistics, the data points behind means, medians and variance measures should be available. If there are restrictions on publicly sharing data—e.g. participant privacy or use of data from a third party—those must be specified.

Reviewer #1: No

Reviewer #2: No

Reviewer #3: Yes

4. Is the manuscript presented in an intelligible fashion and written in standard English?

PLOS ONE does not copyedit accepted manuscripts, so the language in submitted articles must be clear, correct, and unambiguous. Any typographical or grammatical errors should be corrected at revision, so please note any specific errors here.

Reviewer #1: Yes

Reviewer #2: Yes

Reviewer #3: Yes

5. Review Comments to the Author

Please use the space provided to explain your answers to the questions above. You may also include additional comments for the author, including concerns about dual publication, research ethics, or publication ethics. (Please upload your review as an attachment if it exceeds 20,000 characters)

Reviewer #1: Thank you for the opportunity to review this interesting article in which the authors report the findings of a cohort study examining diabetes and CVD medication prescribing among people with T2DM. The article is well written and very clear. The strength of this study lies in the analyses of diabetes medication prescribing, with authors looking at prescription redemption over a long period of time while accounting for diabetes duration. The findings on CVD prescribing are interesting, but subject to more limitations, since the authors did not have information on history of hypertension or cholesterol/lipid levels and so did not look at prescribing according to presence/absence of cardiovascular risk factors. Although they did have information on history of established cardiovascular disease (such as atherosclerotic cardiovascular disease), they did not compared CVD prescribing by SMI status in this sub-group. Findings are presented for all SMI combined, and as the authors state, it would be helpful to examine specific subtypes with respect to diabetes and CVD prescribing in future studies. Despite these limitations, this article makes an important contribution to a very important and under-researched area. I have just a few suggestions on how the article may be further improved
Major comments:
1) A key limitation of the findings on CVD prescribing is the lack of information on cardiovascular risk factors, including hypertension and history of lipid abnormalities and the fact that the authors haven’t looked at whether people with CVD risk factors or established disease are being appropriately treated – i.e. they simply looks at redemption of prescriptions. They comment on the rate of prescription redemption with respect to the median values of lipid measurements, but these measures do not seem to have been available on the whole cohort and this should be highlighted on page 10. Also, there are of course limitations in relating aggregate lipid measurements to aggregate rates of prescription post-diabetes diagnosis.

\# Yes, it is correct that not all persons had a lipid measurement at index (= at the time where T2D was diagnosed). The absolute numbers who had a lipid measurement are presented in table 1. We have inserted/highlighted this limitation in the method section (under “covariates”). Due to the retrospective design, we cannot change this, but we also believe that this provides some insight into how often these tests are made at baseline. We inserted the following sentence in the revised manuscript to highlight this limitation: *“Only a subset of the study population had test measurements for these parameters.”*

2) Did the authors consider performing a sensitivity analysis to look at findings among those with established CVD (i.e. history of major cardiovascular disease event), to look at cardio drugs post DM diagnosis. This may be interesting, even in the inevitably smaller sample.

#We have now made a sensitivity analysis. The following text is inserted in the manuscript:

*“Sensitivity analysis*

*Significant determinants for pharmacological cardiovascular treatment include diagnoses like atherosclerotic cardiovascular disease (CVD), heart failure, or renal disease. The analysis was repeated in a subcohort of the study population that included those with a history of any of these comorbidities in addition to T2D (see S1 Table for ICD-10 codes). Notably, disparities in the prevalence of these comorbidities between individuals with and without SMI may be the cause of discrepancies in the redemption rates of cardiovascular medications observed in the main analysis.*

In the result section we inserted this description:

*The sensitivity analysis does not change the interpretation of the results from the main analysis. When comparing individuals with and without SMI in the subcohort with cardiovascular disease, heart failure or renal disease, we found no discernible trend for differences in the overall outcome ("Cardiovascular medications"), indicating that these comorbidities are an artefact in the overall main analysis for this outcome. When assessing the CV medication subclasses (lipid-lowering drugs, calcium antagonists, beta-blockers, and ACEi/ ARBs) in this subcohort, we found that people with SMI continue to receive these medications less frequently than people without SMI.*

The sensitivity analysis made us change the phrasings in the first section of the discussion into:

**Previous version:** *“The opposite was observed for cardiovascular medications, where persons in the SMI group more rarely redeemed a prescription in the first three years after the index”.*

**New version:** *“The opposite was observed for most cardiovascular medications, where persons in the SMI group more rarely redeemed a prescription”.*

This is the result of the sensitivity analysis:

|  | Cardiovascular medications | | | | | | | | | |
| --- | --- | --- | --- | --- | --- | --- | --- | --- | --- | --- |
|  |  | **Unadjusted rate ratios** | | | |  | **Adjusted* rate ratios** | | | |
| year number after T2D diagnosis | time-period after T2D diagnosis | RR | Lover limit of the 95% CI | Upper limit of the 95% CI | p-value |  | RR | Lover limit of the 95% CI | Upper limit of the 95% CI | p-value |
| 1 | 0-6 months | 0.94 | 0.90 | 0.98 | 0.01 |  | 0.96 | 0.92 | 1.00 | 0.05 |
| 1 | 6-12 months | 0.96 | 0.91 | 1.00 | 0.07 |  | 0.97 | 0.93 | 1.02 | 0.29 |
| 2 | 12-18 months | 0.91 | 0.87 | 0.96 | 0.00 |  | 0.93 | 0.89 | 0.98 | 0.01 |
| 2 | 18-24 months | 0.93 | 0.88 | 0.98 | 0.01 |  | 0.95 | 0.90 | 1.00 | 0.06 |
| 3 | 24-30 months | 0.90 | 0.85 | 0.96 | 0.00 |  | 0.92 | 0.87 | 0.98 | 0.01 |
| 3 | 30-36 months | 0.93 | 0.88 | 0.98 | 0.01 |  | 0.95 | 0.90 | 1.00 | 0.07 |
| 4 | 36-42 months | 0.91 | 0.86 | 0.97 | 0.00 |  | 0.94 | 0.89 | 0.99 | 0.02 |
| 4 | 42-48 months | 0.90 | 0.85 | 0.96 | 0.00 |  | 0.92 | 0.87 | 0.98 | 0.01 |
| 5 | 48-54 months | 0.94 | 0.89 | 1.00 | 0.04 |  | 0.96 | 0.91 | 1.02 | 0.19 |
| 5 | 54-60 months | 0.94 | 0.88 | 1.01 | 0.10 |  | 0.96 | 0.90 | 1.03 | 0.31 |
| 6 | 60-66 months | 0.91 | 0.85 | 0.97 | 0.00 |  | 0.93 | 0.87 | 0.99 | 0.03 |
| 6 | 66-72 months | 0.95 | 0.89 | 1.02 | 0.15 |  | 0.97 | 0.90 | 1.04 | 0.38 |
| 7 | 72-78 months | 0.97 | 0.91 | 1.03 | 0.34 |  | 0.99 | 0.93 | 1.06 | 0.80 |
| 7 | 78-84 months | 0.90 | 0.83 | 0.98 | 0.01 |  | 0.93 | 0.86 | 1.01 | 0.07 |
| 8 | 84-90 months | 0.89 | 0.82 | 0.97 | 0.01 |  | 0.92 | 0.85 | 1.00 | 0.05 |
| 8 | 90-96 months | 0.95 | 0.86 | 1.04 | 0.23 |  | 0.97 | 0.89 | 1.07 | 0.57 |
| 9 | 96-102 months | 0.93 | 0.84 | 1.02 | 0.13 |  | 0.95 | 0.87 | 1.05 | 0.35 |
| 9 | 102-108 months | 0.98 | 0.90 | 1.06 | 0.63 |  | 1.01 | 0.93 | 1.09 | 0.81 |
| 10 | 108-114 months | 0.94 | 0.85 | 1.03 | 0.18 |  | 0.96 | 0.87 | 1.06 | 0.45 |
| 10 | 114-120 months | 0.96 | 0.87 | 1.06 | 0.41 |  | 0.99 | 0.89 | 1.09 | 0.81 |
|  |  |  |  |  |  |  |  |  |  |  |
|  | Lipid-lowering agents | | | | | | | | | |
|  |  | **Unadjusted rate ratios** | | | |  | **Adjusted* rate ratios** | | | |
| year number after T2D diagnosis | time-period after T2D diagnosis | RR | Lover limit of the 95% CI | Upper limit of the 95% CI | p-value |  | RR | Lover limit of the 95% CI | Upper limit of the 95% CI | p-value |
| 1 | 0-6 months | 0.78 | 0.69 | 0.89 | 0.00 |  | 0.81 | 0.72 | 0.92 | 0.00 |
| 1 | 6-12 months | 0.86 | 0.76 | 0.97 | 0.01 |  | 0.89 | 0.79 | 1.00 | 0.06 |
| 2 | 12-18 months | 0.80 | 0.70 | 0.91 | 0.00 |  | 0.83 | 0.73 | 0.95 | 0.00 |
| 2 | 18-24 months | 0.79 | 0.70 | 0.90 | 0.00 |  | 0.83 | 0.73 | 0.94 | 0.00 |
| 3 | 24-30 months | 0.79 | 0.70 | 0.90 | 0.00 |  | 0.82 | 0.73 | 0.94 | 0.00 |
| 3 | 30-36 months | 0.86 | 0.76 | 0.97 | 0.01 |  | 0.90 | 0.80 | 1.01 | 0.07 |
| 4 | 36-42 months | 0.82 | 0.72 | 0.93 | 0.00 |  | 0.86 | 0.76 | 0.97 | 0.01 |
| 4 | 42-48 months | 0.86 | 0.76 | 0.97 | 0.01 |  | 0.90 | 0.80 | 1.01 | 0.08 |
| 5 | 48-54 months | 0.89 | 0.79 | 1.01 | 0.06 |  | 0.94 | 0.83 | 1.06 | 0.30 |
| 5 | 54-60 months | 0.89 | 0.78 | 1.01 | 0.07 |  | 0.92 | 0.81 | 1.06 | 0.24 |
| 6 | 60-66 months | 0.90 | 0.79 | 1.02 | 0.11 |  | 0.93 | 0.82 | 1.06 | 0.27 |
| 6 | 66-72 months | 0.89 | 0.77 | 1.02 | 0.09 |  | 0.92 | 0.80 | 1.05 | 0.21 |
| 7 | 72-78 months | 0.88 | 0.76 | 1.02 | 0.09 |  | 0.91 | 0.79 | 1.05 | 0.21 |
| 7 | 78-84 months | 0.88 | 0.76 | 1.03 | 0.11 |  | 0.92 | 0.78 | 1.07 | 0.28 |
| 8 | 84-90 months | 0.85 | 0.72 | 1.00 | 0.05 |  | 0.88 | 0.75 | 1.05 | 0.15 |
| 8 | 90-96 months | 0.90 | 0.76 | 1.06 | 0.19 |  | 0.93 | 0.78 | 1.10 | 0.39 |
| 9 | 96-102 months | 0.89 | 0.75 | 1.05 | 0.17 |  | 0.92 | 0.77 | 1.10 | 0.36 |
| 9 | 102-108 months | 1.01 | 0.87 | 1.18 | 0.86 |  | 1.06 | 0.90 | 1.24 | 0.48 |
| 10 | 108-114 months | 0.96 | 0.81 | 1.14 | 0.64 |  | 1.00 | 0.84 | 1.20 | 0.97 |
| 10 | 114-120 months | 1.02 | 0.87 | 1.20 | 0.77 |  | 1.07 | 0.91 | 1.26 | 0.41 |
|  |  |  |  |  |  |  |  |  |  |  |
|  | Platelet inhibitors | | | | | | | | | |
|  |  | **Unadjusted rate ratios** | | | |  | **Adjusted* rate ratios** | | | |
| year number after T2D diagnosis | time-period after T2D diagnosis | RR | Lover limit of the 95% CI | Upper limit of the 95% CI | p-value |  | RR | Lover limit of the 95% CI | Upper limit of the 95% CI | p-value |
| 1 | 0-6 months | 0.90 | 0.80 | 1.00 | 0.06 |  | 0.97 | 0.87 | 1.08 | 0.55 |
| 1 | 6-12 months | 0.94 | 0.84 | 1.06 | 0.32 |  | 1.03 | 0.92 | 1.14 | 0.64 |
| 2 | 12-18 months | 0.81 | 0.71 | 0.93 | 0.00 |  | 0.88 | 0.78 | 1.00 | 0.06 |
| 2 | 18-24 months | 0.88 | 0.78 | 1.00 | 0.05 |  | 0.96 | 0.85 | 1.08 | 0.47 |
| 3 | 24-30 months | 0.85 | 0.75 | 0.97 | 0.02 |  | 0.92 | 0.81 | 1.04 | 0.19 |
| 3 | 30-36 months | 0.86 | 0.75 | 0.98 | 0.02 |  | 0.93 | 0.82 | 1.06 | 0.26 |
| 4 | 36-42 months | 0.85 | 0.75 | 0.98 | 0.02 |  | 0.92 | 0.81 | 1.05 | 0.24 |
| 4 | 42-48 months | 0.82 | 0.71 | 0.95 | 0.01 |  | 0.89 | 0.77 | 1.02 | 0.10 |
| 5 | 48-54 months | 0.83 | 0.72 | 0.96 | 0.01 |  | 0.90 | 0.78 | 1.04 | 0.17 |
| 5 | 54-60 months | 0.84 | 0.72 | 0.99 | 0.04 |  | 0.91 | 0.78 | 1.07 | 0.25 |
| 6 | 60-66 months | 0.83 | 0.70 | 0.98 | 0.03 |  | 0.90 | 0.76 | 1.05 | 0.18 |
| 6 | 66-72 months | 0.86 | 0.73 | 1.02 | 0.08 |  | 0.93 | 0.80 | 1.09 | 0.39 |
| 7 | 72-78 months | 0.92 | 0.78 | 1.08 | 0.30 |  | 0.98 | 0.84 | 1.15 | 0.84 |
| 7 | 78-84 months | 0.82 | 0.68 | 1.00 | 0.05 |  | 0.89 | 0.74 | 1.07 | 0.21 |
| 8 | 84-90 months | 0.83 | 0.68 | 1.01 | 0.06 |  | 0.90 | 0.75 | 1.09 | 0.30 |
| 8 | 90-96 months | 0.81 | 0.66 | 1.01 | 0.06 |  | 0.87 | 0.71 | 1.08 | 0.20 |
| 9 | 96-102 months | 0.83 | 0.67 | 1.02 | 0.08 |  | 0.88 | 0.71 | 1.09 | 0.23 |
| 9 | 102-108 months | 0.80 | 0.62 | 1.02 | 0.07 |  | 0.85 | 0.66 | 1.08 | 0.18 |
| 10 | 108-114 months | 0.87 | 0.69 | 1.09 | 0.22 |  | 0.92 | 0.73 | 1.16 | 0.50 |
| 10 | 114-120 months | 0.84 | 0.65 | 1.10 | 0.20 |  | 0.90 | 0.69 | 1.16 | 0.42 |
|  |  |  |  |  |  |  |  |  |  |  |
|  | Diuretics | | | | | | | | | |
|  |  | **Unadjusted rate ratios** | | | |  | **Adjusted* rate ratios** | | | |
| year number after T2D diagnosis | time-period after T2D diagnosis | RR | Lover limit of the 95% CI | Upper limit of the 95% CI | p-value |  | RR | Lover limit of the 95% CI | Upper limit of the 95% CI | p-value |
| 1 | 0-6 months | 1.04 | 0.92 | 1.17 | 0.55 |  | 1.08 | 0.96 | 1.20 | 0.21 |
| 1 | 6-12 months | 1.03 | 0.90 | 1.17 | 0.68 |  | 1.07 | 0.95 | 1.21 | 0.26 |
| 2 | 12-18 months | 0.96 | 0.83 | 1.10 | 0.54 |  | 1.01 | 0.88 | 1.16 | 0.87 |
| 2 | 18-24 months | 0.93 | 0.80 | 1.09 | 0.37 |  | 1.00 | 0.87 | 1.15 | 1.00 |
| 3 | 24-30 months | 0.99 | 0.86 | 1.15 | 0.93 |  | 1.07 | 0.93 | 1.23 | 0.34 |
| 3 | 30-36 months | 0.90 | 0.76 | 1.06 | 0.22 |  | 0.97 | 0.83 | 1.14 | 0.73 |
| 4 | 36-42 months | 0.96 | 0.82 | 1.12 | 0.60 |  | 1.03 | 0.89 | 1.19 | 0.74 |
| 4 | 42-48 months | 0.91 | 0.76 | 1.08 | 0.27 |  | 0.97 | 0.82 | 1.14 | 0.68 |
| 5 | 48-54 months | 0.93 | 0.77 | 1.11 | 0.41 |  | 0.99 | 0.84 | 1.17 | 0.90 |
| 5 | 54-60 months | 0.90 | 0.73 | 1.11 | 0.32 |  | 0.97 | 0.80 | 1.18 | 0.74 |
| 6 | 60-66 months | 0.97 | 0.80 | 1.17 | 0.74 |  | 1.03 | 0.87 | 1.23 | 0.72 |
| 6 | 66-72 months | 1.01 | 0.83 | 1.23 | 0.91 |  | 1.09 | 0.91 | 1.31 | 0.35 |
| 7 | 72-78 months | 1.03 | 0.84 | 1.27 | 0.77 |  | 1.14 | 0.94 | 1.37 | 0.19 |
| 7 | 78-84 months | 0.98 | 0.78 | 1.24 | 0.89 |  | 1.11 | 0.90 | 1.38 | 0.33 |
| 8 | 84-90 months | 0.97 | 0.76 | 1.23 | 0.79 |  | 1.10 | 0.87 | 1.38 | 0.44 |
| 8 | 90-96 months | 1.04 | 0.81 | 1.34 | 0.74 |  | 1.19 | 0.95 | 1.50 | 0.14 |
| 9 | 96-102 months | 1.10 | 0.85 | 1.41 | 0.47 |  | 1.25 | 0.99 | 1.57 | 0.06 |
| 9 | 102-108 months | 0.98 | 0.73 | 1.31 | 0.89 |  | 1.10 | 0.85 | 1.44 | 0.46 |
| 10 | 108-114 months | 0.91 | 0.66 | 1.26 | 0.57 |  | 1.02 | 0.76 | 1.37 | 0.91 |
| 10 | 114-120 months | 1.01 | 0.73 | 1.40 | 0.94 |  | 1.14 | 0.85 | 1.54 | 0.39 |
|  |  |  |  |  |  |  |  |  |  |  |
|  | Calcium antagonists | | | | | | | | | |
|  |  | **Unadjusted rate ratios** | | | |  | **Adjusted* rate ratios** | | | |
| year number after T2D diagnosis | time-period after T2D diagnosis | RR | Lover limit of the 95% CI | Upper limit of the 95% CI | p-value |  | RRa | Lover limit of the 95% CI | Upper limit of the 95% CI | p-value |
| 1 | 0-6 months | 0.75 | 0.60 | 0.94 | 0.01 |  | 0.78 | 0.62 | 0.98 | 0.04 |
| 1 | 6-12 months | 0.81 | 0.64 | 1.01 | 0.06 |  | 0.85 | 0.67 | 1.06 | 0.15 |
| 2 | 12-18 months | 0.69 | 0.54 | 0.89 | 0.00 |  | 0.72 | 0.56 | 0.93 | 0.01 |
| 2 | 18-24 months | 0.69 | 0.53 | 0.90 | 0.01 |  | 0.73 | 0.56 | 0.94 | 0.02 |
| 3 | 24-30 months | 0.63 | 0.48 | 0.83 | 0.00 |  | 0.66 | 0.50 | 0.87 | 0.00 |
| 3 | 30-36 months | 0.72 | 0.56 | 0.93 | 0.01 |  | 0.76 | 0.59 | 0.98 | 0.04 |
| 4 | 36-42 months | 0.72 | 0.55 | 0.92 | 0.01 |  | 0.76 | 0.59 | 0.98 | 0.03 |
| 4 | 42-48 months | 0.73 | 0.56 | 0.95 | 0.02 |  | 0.77 | 0.60 | 1.00 | 0.05 |
| 5 | 48-54 months | 0.81 | 0.62 | 1.04 | 0.10 |  | 0.85 | 0.66 | 1.10 | 0.22 |
| 5 | 54-60 months | 0.65 | 0.48 | 0.89 | 0.01 |  | 0.69 | 0.51 | 0.94 | 0.02 |
| 6 | 60-66 months | 0.63 | 0.46 | 0.88 | 0.01 |  | 0.67 | 0.48 | 0.93 | 0.02 |
| 6 | 66-72 months | 0.64 | 0.46 | 0.89 | 0.01 |  | 0.67 | 0.48 | 0.94 | 0.02 |
| 7 | 72-78 months | 0.66 | 0.47 | 0.93 | 0.02 |  | 0.70 | 0.50 | 0.98 | 0.04 |
| 7 | 78-84 months | 0.56 | 0.38 | 0.83 | 0.00 |  | 0.60 | 0.40 | 0.88 | 0.01 |
| 8 | 84-90 months | 0.56 | 0.38 | 0.84 | 0.01 |  | 0.60 | 0.40 | 0.90 | 0.01 |
| 8 | 90-96 months | 0.65 | 0.44 | 0.97 | 0.03 |  | 0.70 | 0.47 | 1.03 | 0.07 |
| 9 | 96-102 months | 0.70 | 0.47 | 1.04 | 0.08 |  | 0.74 | 0.50 | 1.11 | 0.14 |
| 9 | 102-108 months | 0.80 | 0.54 | 1.18 | 0.26 |  | 0.85 | 0.58 | 1.25 | 0.41 |
| 10 | 108-114 months | 0.78 | 0.52 | 1.18 | 0.24 |  | 0.83 | 0.55 | 1.25 | 0.38 |
| 10 | 114-120 months | 0.85 | 0.56 | 1.28 | 0.43 |  | 0.89 | 0.59 | 1.35 | 0.60 |
|  |  |  |  |  |  |  |  |  |  |  |
|  | Agents acting on the renin-angiotensin system | | | | | | | | | |
|  |  | **Unadjusted rate ratios** | | | |  | **Adjusted* rate ratios** | | | |
| year number after T2D diagnosis | time-period after T2D diagnosis | RR | Lover limit of the 95% CI | Upper limit of the 95% CI | p-value |  | RR | Lover limit of the 95% CI | Upper limit of the 95% CI | p-value |
| 1 | 0-6 months | 0.78 | 0.67 | 0.89 | 0.00 |  | 0.80 | 0.70 | 0.92 | 0.00 |
| 1 | 6-12 months | 0.72 | 0.61 | 0.84 | 0.00 |  | 0.74 | 0.64 | 0.86 | 0.00 |
| 2 | 12-18 months | 0.75 | 0.65 | 0.88 | 0.00 |  | 0.78 | 0.68 | 0.91 | 0.00 |
| 2 | 18-24 months | 0.74 | 0.64 | 0.86 | 0.00 |  | 0.77 | 0.66 | 0.90 | 0.00 |
| 3 | 24-30 months | 0.78 | 0.67 | 0.90 | 0.00 |  | 0.81 | 0.70 | 0.94 | 0.00 |
| 3 | 30-36 months | 0.76 | 0.65 | 0.89 | 0.00 |  | 0.79 | 0.68 | 0.92 | 0.00 |
| 4 | 36-42 months | 0.72 | 0.61 | 0.84 | 0.00 |  | 0.75 | 0.64 | 0.88 | 0.00 |
| 4 | 42-48 months | 0.74 | 0.63 | 0.87 | 0.00 |  | 0.78 | 0.66 | 0.91 | 0.00 |
| 5 | 48-54 months | 0.76 | 0.65 | 0.89 | 0.00 |  | 0.80 | 0.68 | 0.94 | 0.01 |
| 5 | 54-60 months | 0.74 | 0.62 | 0.89 | 0.00 |  | 0.77 | 0.65 | 0.92 | 0.00 |
| 6 | 60-66 months | 0.73 | 0.61 | 0.88 | 0.00 |  | 0.75 | 0.63 | 0.90 | 0.00 |
| 6 | 66-72 months | 0.76 | 0.63 | 0.91 | 0.00 |  | 0.78 | 0.65 | 0.93 | 0.01 |
| 7 | 72-78 months | 0.71 | 0.58 | 0.86 | 0.00 |  | 0.73 | 0.60 | 0.89 | 0.00 |
| 7 | 78-84 months | 0.73 | 0.59 | 0.90 | 0.00 |  | 0.76 | 0.62 | 0.94 | 0.01 |
| 8 | 84-90 months | 0.75 | 0.61 | 0.92 | 0.01 |  | 0.78 | 0.63 | 0.96 | 0.02 |
| 8 | 90-96 months | 0.79 | 0.64 | 0.98 | 0.03 |  | 0.82 | 0.67 | 1.02 | 0.07 |
| 9 | 96-102 months | 0.79 | 0.63 | 0.99 | 0.04 |  | 0.83 | 0.66 | 1.03 | 0.09 |
| 9 | 102-108 months | 0.77 | 0.60 | 0.98 | 0.04 |  | 0.81 | 0.64 | 1.03 | 0.08 |
| 10 | 108-114 months | 0.74 | 0.56 | 0.97 | 0.03 |  | 0.77 | 0.59 | 1.01 | 0.05 |
| 10 | 114-120 months | 0.73 | 0.55 | 0.97 | 0.03 |  | 0.77 | 0.58 | 1.01 | 0.06 |
|  |  |  |  |  |  |  |  |  |  |  |
|  | Beta blocking agents | | | | | | | | | |
|  |  | **Unadjusted rate ratios** | | | |  | **Adjusted* rate ratios** | | | |
| year number after T2D diagnosis | time-period after T2D diagnosis | RR | Lover limit of the 95% CI | Upper limit of the 95% CI | p-value |  | RR | Lover limit of the 95% CI | Upper limit of the 95% CI | p-value |
| 1 | 0-6 months | 0.64 | 0.53 | 0.78 | 0.00 |  | 0.68 | 0.56 | 0.83 | 0.00 |
| 1 | 6-12 months | 0.72 | 0.60 | 0.87 | 0.00 |  | 0.77 | 0.64 | 0.93 | 0.01 |
| 2 | 12-18 months | 0.66 | 0.54 | 0.81 | 0.00 |  | 0.71 | 0.58 | 0.87 | 0.00 |
| 2 | 18-24 months | 0.70 | 0.57 | 0.85 | 0.00 |  | 0.75 | 0.62 | 0.91 | 0.00 |
| 3 | 24-30 months | 0.69 | 0.56 | 0.84 | 0.00 |  | 0.74 | 0.61 | 0.91 | 0.00 |
| 3 | 30-36 months | 0.68 | 0.55 | 0.84 | 0.00 |  | 0.74 | 0.61 | 0.91 | 0.00 |
| 4 | 36-42 months | 0.63 | 0.50 | 0.78 | 0.00 |  | 0.68 | 0.55 | 0.84 | 0.00 |
| 4 | 42-48 months | 0.66 | 0.53 | 0.82 | 0.00 |  | 0.71 | 0.57 | 0.88 | 0.00 |
| 5 | 48-54 months | 0.72 | 0.58 | 0.90 | 0.00 |  | 0.78 | 0.63 | 0.97 | 0.03 |
| 5 | 54-60 months | 0.73 | 0.58 | 0.93 | 0.01 |  | 0.79 | 0.62 | 1.00 | 0.05 |
| 6 | 60-66 months | 0.66 | 0.51 | 0.86 | 0.00 |  | 0.70 | 0.54 | 0.91 | 0.01 |
| 6 | 66-72 months | 0.65 | 0.49 | 0.86 | 0.00 |  | 0.68 | 0.52 | 0.90 | 0.01 |
| 7 | 72-78 months | 0.63 | 0.47 | 0.85 | 0.00 |  | 0.67 | 0.50 | 0.90 | 0.01 |
| 7 | 78-84 months | 0.58 | 0.41 | 0.81 | 0.00 |  | 0.63 | 0.45 | 0.88 | 0.01 |
| 8 | 84-90 months | 0.54 | 0.38 | 0.78 | 0.00 |  | 0.59 | 0.41 | 0.84 | 0.00 |
| 8 | 90-96 months | 0.68 | 0.48 | 0.94 | 0.02 |  | 0.74 | 0.53 | 1.03 | 0.07 |
| 9 | 96-102 months | 0.66 | 0.47 | 0.93 | 0.02 |  | 0.71 | 0.51 | 1.01 | 0.05 |
| 9 | 102-108 months | 0.68 | 0.47 | 0.99 | 0.04 |  | 0.74 | 0.51 | 1.07 | 0.11 |
| 10 | 108-114 months | 0.70 | 0.48 | 1.01 | 0.06 |  | 0.75 | 0.52 | 1.09 | 0.13 |
| 10 | 114-120 months | 0.72 | 0.49 | 1.06 | 0.09 |  | 0.79 | 0.54 | 1.16 | 0.23 |
|  |  |  |  |  |  |  |  |  |  |  |
|  | Cardiac therapy | | | | | | | | | |
|  |  | **Unadjusted rate ratios** | | | |  | **Adjusted* rate ratios** | | | |
| year number after T2D diagnosis | time-period after T2D diagnosis | RR | Lover limit of the 95% CI | Upper limit of the 95% CI | p-value |  | RRa | Lover limit of the 95% CI | Upper limit of the 95% CI | p-value |
| 1 | 0-6 months | 0.89 | 0.72 | 1.10 | 0.29 |  | 0.96 | 0.79 | 1.18 | 0.72 |
| 1 | 6-12 months | 1.04 | 0.84 | 1.28 | 0.72 |  | 1.14 | 0.94 | 1.39 | 0.19 |
| 2 | 12-18 months | 0.94 | 0.75 | 1.18 | 0.60 |  | 1.05 | 0.85 | 1.30 | 0.66 |
| 2 | 18-24 months | 0.97 | 0.77 | 1.23 | 0.82 |  | 1.09 | 0.87 | 1.37 | 0.43 |
| 3 | 24-30 months | 0.90 | 0.69 | 1.16 | 0.40 |  | 1.02 | 0.80 | 1.29 | 0.89 |
| 3 | 30-36 months | 0.90 | 0.69 | 1.17 | 0.42 |  | 1.02 | 0.80 | 1.31 | 0.85 |
| 4 | 36-42 months | 0.84 | 0.64 | 1.11 | 0.22 |  | 0.94 | 0.73 | 1.21 | 0.66 |
| 4 | 42-48 months | 0.81 | 0.60 | 1.08 | 0.15 |  | 0.90 | 0.69 | 1.19 | 0.47 |
| 5 | 48-54 months | 0.93 | 0.70 | 1.24 | 0.61 |  | 1.05 | 0.81 | 1.37 | 0.71 |
| 5 | 54-60 months | 0.87 | 0.63 | 1.20 | 0.40 |  | 1.00 | 0.74 | 1.34 | 0.98 |
| 6 | 60-66 months | 0.95 | 0.69 | 1.31 | 0.75 |  | 1.07 | 0.80 | 1.43 | 0.64 |
| 6 | 66-72 months | 1.04 | 0.75 | 1.43 | 0.82 |  | 1.19 | 0.89 | 1.60 | 0.24 |
| 7 | 72-78 months | 0.69 | 0.45 | 1.06 | 0.09 |  | 0.81 | 0.55 | 1.21 | 0.31 |
| 7 | 78-84 months | 0.74 | 0.48 | 1.14 | 0.18 |  | 0.90 | 0.60 | 1.36 | 0.62 |
| 8 | 84-90 months | 0.78 | 0.51 | 1.21 | 0.27 |  | 0.95 | 0.63 | 1.43 | 0.79 |
| 8 | 90-96 months | 0.89 | 0.57 | 1.37 | 0.58 |  | 1.09 | 0.73 | 1.62 | 0.69 |
| 9 | 96-102 months | 0.81 | 0.50 | 1.31 | 0.39 |  | 0.98 | 0.63 | 1.53 | 0.93 |
| 9 | 102-108 months | 0.80 | 0.47 | 1.36 | 0.42 |  | 0.96 | 0.59 | 1.56 | 0.87 |
| 10 | 108-114 months | 0.68 | 0.37 | 1.24 | 0.21 |  | 0.80 | 0.46 | 1.38 | 0.42 |
| 10 | 114-120 months | 0.70 | 0.37 | 1.32 | 0.27 |  | 0.83 | 0.46 | 1.50 | 0.54 |

- * Adjusted for sex, age (quadratic), glycaemic control at index date, comorbidities preceding the index date (S1 Table), calendar year and level of education.

3) On page 20 of discussion, the authors hypothesize that there is a higher mortality in the SMI group, leading to a survival bias with “more robust” people with SMI being included in the later follow-up. The difference in mortality is likely, but again presumably the authors could simply look at this to support this statement with data rather than hypothesis?

\# Based on your comment we made an analysis of the mortality in the SMI group versus the non-SMI group. From this, it became apparent that mortality is higher in the SMI group after adjusting for several variables, as we also hypothesized (please see table below).

Therefore, we have changed our phrasing in the discussion to the following:

*“We observed higher mortality in the SMI group, which will likely lead to a selection of the more "robust" persons in the SMI group later in the follow-up. While mortality is not high and the influence of such selection is limited, the higher mortality in the SMI group would if anything, probably lead to lower medicine use”*

|  |  | **Unadjusted rate ratios** | | | |  | **Adjusted* rate ratios** | | | |
| --- | --- | --- | --- | --- | --- | --- | --- | --- | --- | --- |
| Years after T2D diagnosis | Specific time-period after T2D diagnosis | RR | Lower limit of the 95% CI | Upper limit of the 95% CI | p-value |  | RR | Lower limit of the 95% CI | Upper limit of the 95% CI | p-value |
| 1 | 0-6 months | 1.71 | 1.29 | 2.27 | 0.00 |  | 2.33 | 1.72 | 3.15 | 4.14E-08 |
| 1 | 6-12 months | 1.51 | 1.08 | 2.11 | 0.02 |  | 2.08 | 1.47 | 2.93 | 3.12E-05 |
| 2 | 12-18 months | 1.30 | 0.88 | 1.93 | 0.19 |  | 1.81 | 1.21 | 2.71 | 0.00 |
| 2 | 18-24 months | 1.26 | 0.84 | 1.89 | 0.27 |  | 1.80 | 1.19 | 2.72 | 0.01 |
| 3 | 24-30 months | 1.36 | 0.91 | 2.03 | 0.13 |  | 1.97 | 1.31 | 2.97 | 0.00 |
| 3 | 30-36 months | 1.19 | 0.79 | 1.80 | 0.41 |  | 1.72 | 1.13 | 2.64 | 0.01 |
| 4 | 36-42 months | 1.19 | 0.77 | 1.83 | 0.44 |  | 1.68 | 1.08 | 2.61 | 0.02 |
| 4 | 42-48 months | 1.00 | 0.62 | 1.62 | 1.00 |  | 1.40 | 0.85 | 2.29 | 0.18 |
| 5 | 48-54 months | 1.40 | 0.92 | 2.12 | 0.11 |  | 1.91 | 1.26 | 2.90 | 0.00 |
| 5 | 54-60 months | 1.30 | 0.83 | 2.03 | 0.25 |  | 1.85 | 1.17 | 2.93 | 0.01 |
| 6 | 60-66 months | 1.20 | 0.73 | 1.98 | 0.47 |  | 1.72 | 1.04 | 2.83 | 0.03 |
| 6 | 66-72 months | 1.91 | 1.29 | 2.83 | 0.00 |  | 2.79 | 1.88 | 4.16 | 4.28E-07 |
| 7 | 72-78 months | 1.69 | 1.09 | 2.62 | 0.02 |  | 2.52 | 1.63 | 3.90 | 3.43E-05 |
| 7 | 78-84 months | 1.19 | 0.70 | 2.02 | 0.53 |  | 1.83 | 1.07 | 3.11 | 0.03 |
| 8 | 84-90 months | 1.19 | 0.68 | 2.06 | 0.54 |  | 1.81 | 1.05 | 3.13 | 0.03 |
| 8 | 90-96 months | 0.77 | 0.36 | 1.62 | 0.49 |  | 1.19 | 0.56 | 2.54 | 0.65 |
| 9 | 96-102 months | 0.98 | 0.50 | 1.89 | 0.94 |  | 1.47 | 0.75 | 2.87 | 0.26 |
| 9 | 102-108 months | 1.49 | 0.86 | 2.59 | 0.16 |  | 2.24 | 1.28 | 3.92 | 0.00 |
| 10 | 108-114 months | 1.49 | 0.84 | 2.65 | 0.17 |  | 2.23 | 1.25 | 3.98 | 0.01 |
| 10 | 114-120 months | 1.46 | 0.80 | 2.67 | 0.22 |  | 2.22 | 1.20 | 4.11 | 0.01 |

- * Adjusted for sex, age (quadratic), glycaemic control at index date, comorbidities preceding the index date (S1 Table), calendar year and level of education.

4) The contrasting findings on receipt of diabetes medication prescriptions versus CVD medication prescriptions is very interesting and to some readers this contrast may be surprising. The authors very nicely discuss the findings with respect to other relevant literature. However, although they propose some explanations for the diabetes medication findings, they don’t really provide possible suggestions for the observed CVD medication prescribing. Can the authors expand on this a little?

#Thank you. We find this contrast interesting too. Our data do not provide an explanation for this. Some other Danish studies (please refer to references below) indicate that people with SMI are often not sufficiently treated with cardiovascular medication (primary and secondary prevention) and this might contribute to the results that we also found.

Based on this comment we have rewritten the section “Differences in the use of cardiovascular medications” to reflect more on the differences in the discussion.

To investigate why some types of medications are prioritized higher than others further studies are needed. We added this consideration to the Discussion section, please also see our answer to your comment no. 5.

[Kugathasan P, Horsdal HT, Aagaard J, Jensen SE, Laursen TM, Nielsen RE. Association of Secondary Preventive Cardiovascular Treatment After Myocardial Infarction With Mortality Among Patients With Schizophrenia. JAMA Psychiatry. 2018 Dec 1;75(12):1234-1240. doi: 10.1001/jamapsychiatry.2018.2742. Erratum in: JAMA Psychiatry. 2019 May 1;: PMID: 30422158; PMCID: PMC6583028.]

[Laursen TM, Mortensen PB, MacCabe JH, Cohen D, Gasse C. Cardiovascular drug use and mortality in patients with schizophrenia or bipolar disorder: a Danish population-based study. Psychol Med. 2014 Jun;44(8):1625-37. doi: 10.1017/S003329171300216X. Epub 2013 Nov 18. PMID: 24246137.]

In addition, it seems that there may be marginally less attention to dyslipidemia in the SMI group if we refer to the number of tests made at baseline (-14 days – 1 months after). In table 1, we presented the absolute numbers in each group, who has a measurement made for LDL-C, total cholesterol, triglycerides, and HDL. In the table below, we have calculated the proportions who had these tests made. Generally, a lower proportion of people with SMI get these lipids measured at baseline, which also suggests that less attention is made to these risk factors among people with SMI.

|  | SMI | Non-SMI |
| --- | --- | --- |
| LDL | 660/1316 =50% | 23138/41538 =56% |
| Total cholesterol | 903/1316 = 69% | 29955/41538= 72% |
| Triglycerides | 785/1316 =59% | 26192/41538 = 63% |
| HDL | 809/1316 = 61% | 26791/41538 = 65% |

It is not possible to calculate how many people had blood pressure measurements as this information is not available in the registers.

5) What do the authors consider to be the implications of the research for health policy/practice and research, particularly with a view to understanding the possible opposing results on diabetes and CVD prescribing?

\# We have added this paragraph in the discussion:

“*From a clinical standpoint, our results indicate that among people with pre-existing SMI, metformin is generally used more in the initial years after the T2D diagnosis. The apparently opposing results regarding CVD prescribing suggest that there might be room for improvements in the treatment of cardiac risk factors (i.e., hypertension and dyslipidemia) among people with SMI and T2D. Further studies are warranted to explore if and why glucose-lowering medications are prioritized higher than CVD medications among people with SMI; this can be done with qualitative studies, for example”.*

6) The authors do not state in the methods whether they adhered to suitable reporting guidelines (i.e. RECORD).

\# We omitted to mention this in the article but followed the STROBE statements. This information has been added to the manuscript now. The current version of the article does not follow RECORD completely but if you wish, we can make a few corrections in the article to make the article fulfill the RECORD criteria too.

Added this sentence in the method section: *“*This article is written in accordance with the "Strengthening the Reporting of Observational Studies in Epidemiology (STROBE)" statements. *“.*

[von Elm E, Altman DG, Egger M, Pocock SJ, Gøtzsche PC, Vandenbroucke JP; STROBE Initiative. The Strengthening the Reporting of Observational Studies in Epidemiology (STROBE) Statement: guidelines for reporting observational studies. Int J Surg. 2014 Dec;12(12):1495-9. doi: 10.1016/j.ijsu.2014.07.013. Epub 2014 Jul 18. PMID: 25046131.]

Minor comments:
7) Supplementary tables – could add footnote to indicate what variables RRs are adjusted for.

\# Added the red text in the legend of the S1 table: “**S1 Table: Comorbidities.** Categorized based on International Statistical Classification System of Diseases editions 8 and 10. *Each of these conditions is seen as a possible confounding factor and included in the statistical analyses.”*

In the S2 and S3 Tables we have added the following description as a footnote:

*“*Adjusted for sex, age (quadratic), glycaemic control at the index date, comorbidities preceding the index date (S1 Table), calendar year, and level of education”*

Reviewer #2: This is a study of pharmacoutilization in diabetic patients with or without severe mental illness. The area of research is interesting and potentially relevant for public health since the need to understand the causes for higher cardiovascular morbidity and mortality in people with SMI. The methods for defining cases and controls is sound, the sample representative and the duration of follow-up adequate. The authors find that diabetic patients with SMI are prescribed more antidiabetic medications and less cardiovascular medications in the initial years after the diagnosis of diabetes.
1) However, there is a huge methodological issue: diabetes is diagnosed out of a single assessment of elevated HbA1c or glycemia levels.
International criteria for diagnosing diabetes require either two independent assessment of glycemia ≥ 7 mmol/l or HbA1c ≥ 6.5, or one assessment coupled with the presence of symptoms of diabetes, such as polyuria, polydipsia, and glucosuria. A random plasma glucose ≥ 11 mmol/l could diagnose diabetes only in patients with symptoms of hyperglycemia. Recently, a study highlighted that high levels of both glycemia and HbA1c in the same sample also allows diagnosing diabetes with high accuracy (Selvin et al., 2018). However, I am afraid that diagnosing diabetes out of a single blood test may lead to false positives.

\# We agree that we might have some false positives for persons with T2D included in this study. However, we believe that this bias is equal in both groups. The variability in the Danish HbA1c tests is low, and therefore repetition of tests is mainly due to the risk of exchanged tests (DSAM reference page 9, please see below, unfortunately only in Danish). Another argument for the repetition of tests is that diseases like anaemia and chronic kidney disease may impact HbA1c. Another study (also based on data from the CopLab database) has found that mild to moderate anaemia or mild to moderate chronic kidney disease do not have a significant impact on the relationship between glycemia and HbA1c (please see reference by Borg et al below)

At baseline, 74% of patients in the SMI groups have had an HbA1c test made at the time of diagnosis versus 78% in the non-SMI group; therefore, a large proportion of patients are included based on elevated HbA1c (Table 1 in the manuscript). Knowing that the consequences for classifying a person incorrectly as having T2D can be large for the individual we acknowledge that a diagnostic test should be repeated. But in this register study setting, we believe that the false positives are distributed evenly across the two groups.

We considered including two elevated measurements as the diagnostic criteria. The argument that we did not do so in our final version is that we believe some patients will have a measurement made at the hospital regimen, and then later confirmed at the GPs by use of the CopLab database. A method requiring two measurements in the CopLab would exclude some persons who did have T2D (false negative).

[reference DSAM 2012 <https://vejledninger.dsam.dk/media/files/20/type-2-diabetes-2012-med-links.pdf>) ]

[Borg R, Persson F, Siersma V, Lind B, de Fine Olivarius N, Andersen CL. Interpretation of HbA_1c_ in primary care and potential influence of anaemia and chronic kidney disease: an analysis from the Copenhagen Primary Care Laboratory (CopLab) Database. Diabet Med. 2018 Dec;35(12):1700-1706. doi: 10.1111/dme.13776. Epub 2018 Aug 20. PMID: 29985535.]

2) Although the authors, not being able to tease out the blood tests performed in a fasting condition, correctly considered only remarkably high glycemic values to reduce type I error, still a single measurement of one biomarker only is insufficient for a diagnosis of diabetes.
As a matter of fact, a putative explanation of finding more prescriptions of antidiabetic medications in the SMI group might be that these patients have been more frequently diagnosed with diabetes over time than the non-SMI counterpart. The authors have access to ICD diagnoses in this cohort. Although it’s true that employing a diagnosis based on biomarker levels rules out undiagnosed diabetes, I guess that they should include patients based on relevant ICD codes instead of using an incorrect definition based on a single biomarker observation.

\# We choose not to include ICD-10 codes as these are only used in the hospital sector of the Danish Healthcare system, and we aimed to better understand the diabetes treatment among people who were diagnosed in general practice. The method using only one test measurement to define T2D has been used in other Danish register studies too:

[Heinrich NS, Kriegbaum M, Grand MK, Lind BS, Andersen CL, Persson F. Biochemical profiling, pharmacological management and clinical outcomes in type 2 diabetes in Danish primary care from 2001 to 2015. Prim Care Diabetes. 2022 Dec;16(6):818-823. doi: 10.1016/j.pcd.2022.10.006. Epub 2022 Oct 19. PMID: 36272916.]

[Kristensen FP, Rohde C, Østergaard SD, Thomsen RW. Four-year HbA1c and LDL-cholesterol trajectories among individuals with mental disorders and newly developed type 2 diabetes. Brain Behav. 2021 Nov;11(11):e2372. doi: 10.1002/brb3.2372. Epub 2021 Sep 20. PMID: 34543525; PMCID: PMC8613402.]

[Rohde C, Knudsen JS, Schmitz N, Østergaard SD, Thomsen RW. The impact of hospital-diagnosed depression or use of antidepressants on treatment initiation, adherence and HbA1c/LDL target achievement in newly diagnosed type 2 diabetes. Diabetologia. 2021 Feb;64(2):361-374. doi: 10.1007/s00125-020-05303-4. Epub 2020 Oct 19. PMID: 33073329.]

Reviewer #3: Comments on paper PONE-D-22-16756

This is an interesting paper which uses high quality data from Danish registries to assess prescribing in severe mental illnesses.

1) My main concern with the analysis is that the authors did not seem to have any information on prescribed psychotropic medications- this would include atypical antipsychotic medications, some of which are known to be associated with a high risk of developing T2DM and metabolic syndrome (eg. in particular Olanzapine, Clozapine)/ be associated with CVD risk. In some patients where clinicians are concerned about side effects drugs with lower propensity for CVD risk may be suggested for switching/ initiation- in fact this is recommended in WHO guidelines for the management of physical health in SMI- see this link [https://www.who.int/publications/i/item/978-92-4-155038-3](https://eur02.safelinks.protection.outlook.com/?url=https%3A%2F%2Fwww.who.int%2Fpublications%2Fi%2Fitem%2F978-92-4-155038-3&data=05%7C01%7Cbakkedal%40sund.ku.dk%7Cc6f6465557ec4da9f96e08dac41f2bc9%7Ca3927f91cda14696af898c9f1ceffa91%7C0%7C0%7C638037935582531897%7CUnknown%7CTWFpbGZsb3d8eyJWIjoiMC4wLjAwMDAiLCJQIjoiV2luMzIiLCJBTiI6Ik1haWwiLCJXVCI6Mn0%3D%7C3000%7C%7C%7C&sdata=WL6d34YGMKBbAsN8gyzPWFZHUV4VZzAaW%2BSps2rq4OY%3D&reserved=0) Also using adjunct Metformin is recommended for people who are overweight or obese (see the WHO link). This therefore means that it is impossible to know if the patterns they observed in their dataset simply reflects clinicians following guidelines and/ or are confounded by psychotropic medication prescribing.

\# We hope that we understand your concern correctly. We understand that people in treatment with antipsychotics might receive preventive medications with metformin and that metformin might be used in non-diabetics who are obese, i.e., that metformin can be used for weight loss in this group. In our study design, we excluded people who redeemed a prescription with any type of glucose-lowering medications (including metformin) two years before the index date (= time of T2D diagnosis). If these patients had been included, it would have given a false impression that these patients had initiated metformin immediately.

Our aim was not to show how often guidelines were followed, as guidelines emphasize an individual approach and our registries do not include information to make this individual-based judgement fully, i.e., preferences, weight (obesity), medication adverse effects, family dispositions for CVD, smoking are not included in the registries. We aimed to describe the patterns of how often people with SMI (versus no SMI) used glucose-lowering and CVD medications.

We do have access to psychotropic medications but choose not to include this in the analyses. If we had included psychotropic medications prescriptions there would be some limitations: For people with schizophrenia in Denmark, free antipsychotic medication is available for approximately the first two years after schizophrenia diagnosis (In Denmark this offer is included in an offer called “OPUS”), and since this medication is administrated by personnel at psychiatric departments, this information is not included in the national prescription register. In addition, for people who have been judged to receive treatment for a mental illness, medication will be prescribed locally and not provided by pharmacies; therefore, no information is available in the national prescription register for this either.

I have some further comments below which the authors may wish to consider and it may strengthen the paper:

2) Abstract - this ends rather abruptly. I suggest the authors add a sentence at the end summarising the main conclusions.

\# We agree with this observation but are limited due to word count. Based on your comment we have removed some words from the abstract, which allowed space for the following sentence at the end: *“For people with SMI in addition to T2D, metformin is more likely to be used in the initial years after T2D diagnosis, while our results suggest potential room for improvement regarding the use of cardiovascular medications.”*

3) To understand the findings it would be helpful to have more detail on care provided in Denmark . Are more complex patients with T2DM referred on to secondary care? Do mental health care providers also look after physical health in SMI patients. What are the specific care pathways and is there evidence in inequalities in these impacting people with SMI?

\# Thanks for this comment. We have added the following descriptions:

To the Introduction section:

*In Denmark, people with SMI receive treatment for their mental illness in hospital psychiatry, while their somatic chronic care is primarily treated in general practice*. *More than 80% of people with T2D are taken care of in general practice [25, 26] while only those with severe dysregulation or complications are referred to diabetes specialists, which makes it relevant to explore the treatment in the primary care setting.*

(This change made the introduction rather long in our opinion, and therefore we made some additional changes in the introduction: improved use of verbal style, added en-dash in time periods, rephrased and deleted a few sentences)

To the Methods section:

*“All citizens in Denmark have free and direct access to general practitioners, who can refer patients to biochemical testing without individual payment. In Denmark, general practitioners make up 20% of the physician workforce [29], and have a central role in the Danish Health care system, including acting as gatekeepers to more specialised patient care in addition to handling many somatic chronic disorders, including T2D”.*

4) Figures are a little unclear - please provide a label for the x axis and a footnote for acronyms in the figure. For example in figure 2 what is A10BD?

\# The label for the x-axis is mentioned below the eight figures. We choose not to add a label on the x- axis in each of the small figures as we found this unnecessary repetition. But if you prefer this we can do so, or enlarge the label at the bottom?

To describe what A10BD is the following text is added to the description of Fig 2:

*“**Combinations of oral glucose-lowering medications (A10BD) include metformin + thiazolidinediones, metformin + DPP4i, metformin + SGLT2i, sulfonylureas + thiazolidinediones, DPP4i + thiazolidinediones and SGLT2i + DPP4i”.*

5) In general, the acronyms in the text make it less readable- please consider spelling out where possible.

\# We agree with the reviewer. Therefore, we have made the following adaptions:

NPR, DNPR, DPCR, CGPL, UACR, CCI: These acronyms are removed and spelled out instead.

The paragraph in the method sections stating (F20-29 >F30-31 >F32-33 >F34-39 >F60-69) is changed into: (*schizophrenia spectrum disorders>bipolar disorders > unipolar depression >other affective disorders > personality disorders*). To increase the readability, we have also rewritten the section about the definition of severe mental illness, so that the ICD-10 codes come in parentheses instead.

We are aware that the word “ATC” is used often in the text, and therefore a few paraphrasing are made i.e. the paragraph “For the cardiovascular medications, we investigated each ATC subgroup” is changed to “*For the cardiovascular medications we investigated the following classes:….*”.

To make the text more readable we have also removed some citation marks from the Result section.

6. PLOS authors have the option to publish the peer review history of their article ([what does this mean?](https://eur02.safelinks.protection.outlook.com/?url=https%3A%2F%2Fjournals.plos.org%2Fplosone%2Fs%2Feditorial-and-peer-review-process%23loc-peer-review-history&data=05%7C01%7Cbakkedal%40sund.ku.dk%7Cc6f6465557ec4da9f96e08dac41f2bc9%7Ca3927f91cda14696af898c9f1ceffa91%7C0%7C0%7C638037935582531897%7CUnknown%7CTWFpbGZsb3d8eyJWIjoiMC4wLjAwMDAiLCJQIjoiV2luMzIiLCJBTiI6Ik1haWwiLCJXVCI6Mn0%3D%7C3000%7C%7C%7C&sdata=vtCds025n%2FI%2BTg6trRiktLS%2FH3NxsFmWdP8yyE7wBs8%3D&reserved=0)). If published, this will include your full peer review and any attached files.


If you choose “no”, your identity will remain anonymous but your review may still be made public.


**Do you want your identity to be public for this peer review?** For information about this choice, including consent withdrawal, please see our [Privacy Policy](https://eur02.safelinks.protection.outlook.com/?url=https%3A%2F%2Fwww.plos.org%2Fprivacy-policy&data=05%7C01%7Cbakkedal%40sund.ku.dk%7Cc6f6465557ec4da9f96e08dac41f2bc9%7Ca3927f91cda14696af898c9f1ceffa91%7C0%7C0%7C638037935582531897%7CUnknown%7CTWFpbGZsb3d8eyJWIjoiMC4wLjAwMDAiLCJQIjoiV2luMzIiLCJBTiI6Ik1haWwiLCJXVCI6Mn0%3D%7C3000%7C%7C%7C&sdata=QOG2XA1TNWHldfRozoQsG%2FCCHr2MbOY5DGkNngGRLtA%3D&reserved=0).

Reviewer #1: **Yes:** Caroline Jackson

Reviewer #2: No

Reviewer #3: No

[NOTE: If reviewer comments were submitted as an attachment file, they will be attached to this email and accessible via the submission site. Please log into your account, locate the manuscript record, and check for the action link "View Attachments". If this link does not appear, there are no attachment files.]

While revising your submission, please upload your figure files to the Preflight Analysis and Conversion Engine (PACE) digital diagnostic tool, [https://pacev2.apexcovantage.com/](https://eur02.safelinks.protection.outlook.com/?url=https%3A%2F%2Fpacev2.apexcovantage.com%2F&data=05%7C01%7Cbakkedal%40sund.ku.dk%7Cc6f6465557ec4da9f96e08dac41f2bc9%7Ca3927f91cda14696af898c9f1ceffa91%7C0%7C0%7C638037935582531897%7CUnknown%7CTWFpbGZsb3d8eyJWIjoiMC4wLjAwMDAiLCJQIjoiV2luMzIiLCJBTiI6Ik1haWwiLCJXVCI6Mn0%3D%7C3000%7C%7C%7C&sdata=eiObVvYrPHT6qnKuzaG5OYRp0imWdKMBqhplgwVAzaM%3D&reserved=0). PACE helps ensure that figures meet PLOS requirements. To use PACE, you must first register as a user. Registration is free. Then, login and navigate to the UPLOAD tab, where you will find detailed instructions on how to use the tool. If you encounter any issues or have any questions when using PACE, please email PLOS at [figures@plos.org](mailto:figures@plos.org). Please note that Supporting Information files do not need this step.
