# Supplementary material for: Diabetes treatment for persons with severe mental illness: A registry-based cohort study to explore medication treatment differences for persons with type 2 diabetes with and without severe mental illness
Source: PLoS One. 2023 Jun 13;18(6):e0287017. doi: 10.1371/journal.pone.0287017 (PMC10263345; doi:10.1371/journal.pone.0287017)
Supplement: Attachment — Submitted filename: Response to reviewers.docx [file pone.0287017.s011.docx]

6. Review Comments to the Author

Please use the space provided to explain your answers to the questions above. You may also include additional comments for the author, including concerns about dual publication, research ethics, or publication ethics. (Please upload your review as an attachment if it exceeds 20,000 characters)

Reviewer #1: (No Response)

Reviewer #3: The manuscript is much improved. However my concerns around your general approach, leading to the conclusions, remain.

The main issue is that your definition of 'SMI' is very broad- usually this would just encompass people with schizophrenia-spectrum and bipolar disorders (who are more likely prescribed antipsychotics- including those associated with a higher risk of metabolic syndrome- which as I suggested in my previous review- may be driving the observed patterns). However you have included people with personality disorders and depression in your 'SMI' definition.

Prescribing and risks for CVD/ diabetes may be quite different for these other diagnostic groups. Personality disorders in particular are axis 2 diagnoses and this group may not receive any psychotropic medications. If you are unable to look at types of psychotropic medications prescribed then at the very least I would suggest stratifying or undertaking analyses by psychiatric diagnosis- I would suggest keeping schizophrenia and bipolar disorders separate to depression, and separate to personality disorders. My preference would be to see both stratified analyses by psychiatric diagnoses and a consideration of psychotropic medication prescribing (in particular antipsychotic medications).

Thank you for your thorough review and ideas for improvements.

Based on your comment, we have made a **stratified analysis** of psychiatric diagnoses for some selected central outcomes. We have inserted this additional analysis as supporting information in figures S3, S4, S5, and S6. The figures can be seen below. A few comments on this stratified analysis:

- Due to small numbers in the “Other affective group” (n=13) we did not include this group in the stratified analyses.
- We decided not to merge bipolar disorders and schizophrenia spectrum disorders, even though people in both groups might use antipsychotic drugs. We wanted to examine the difference between these two SMI subgroups now that we made the extra analysis.

Based on the stratified analysis we added the following text to the manuscript:

Page 14:

*“Stratification of the analyses for individual psychiatric diagnoses showed some heterogeneity between the subgroups, i.e., the increased use of metformin initially was primarily driven by a higher use among people with schizophrenia spectrum disorders and bipolar disorders (Fig S3 and S5)”*

Page 15:

*“The stratified analysis showed that the reduced use of cardiovascular medications in the initial years was due to lower use among people with schizophrenia spectrum disorders, bipolar disorders, and personality disorders but not among people with unipolar depression (Fig S4 and S6). For the individual cardiovascular medication classes, the same tendency was seen, i.e., when reduced use was present, it was mainly associated with schizophrenia spectrum disorders and bipolar disorders, but less so with unipolar depression”.*

And deleted this paragraph from page 22:

*We used a broad definition of SMI including several diagnoses, as we aimed to describe the diabetes treatment for this overall vulnerable group. Future studies could try to differentiate the treatment rates between the diagnosis groups to explore if the specific SMI diagnosis influences our results.*

**Fig S3: Crude fraction of glucose-lowering medication use stratified for SMI-subgroups.**

**Fig S4: Crude fraction of cardiovascular medication use stratified for SMI-subgroups.**

**Fig S5. Adjusted rate ratios for glucose-lowering medications stratified for SMI subgroups.**

**Fig S6. Adjusted rate ratios for cardiovascular medications stratified for SMI subgroups.**

We have extensively discussed your suggestion of a stratified psychotropic medication analysis but chose not to do this extra analysis. Allow us to explain our decision. Firstly, for the other possible confounders in this study, we have used the status prior to or at the time of T2D diagnosis (i.e., glycemic control at index, comorbidities prior to index, level of education at index). To align with this strategy, we considered exploring psychotropic medications in the 180 days before T2D diagnosis. However, in our opinion, this type of stratified analysis would be misleading as the use and types of psychotropic medications might have changed often for each individual during follow-up. Secondly, psychotropic medications are not always included in the national prescription registers; for example, (i) not for people within the first approximately two years after a schizophrenia diagnosis (OPUS as mentioned in our last review round), (ii) when medication is given during hospitals admissions or (iii) if people are ordered by the court to undergo mental health treatment, in which case the medications are provided for free and are not included in the national prescription registry. The following report describes when medication is provided for free for people with mental disorders in the Danish health care system (unfortunately in Danish only).

<https://psykiatrienisyddanmark.dk/fagpersoner/radgivning-om-psykofarmaka/artikler-om-psykofarmaka-og-kliniske-retningslinjer/nationale-og-regionale-retningslinjer-og-regler/vederlagsfri-medicin-retningslinje-2021>

We agree that people using antipsychotics, especially clozapine and olanzapine, are at increased risk for metabolic syndrome. However, due to the use of registries, we are unable to evaluate the occurrence of all components in the metabolic syndrome. If we consider the individual components (visceral obesity, dyslipidaemia, hyperglycaemia, and hypertension), we believe that a higher prevalence of these components for people with SMI would have reinforced our argument that dyslipidemia and hypertension may be “undertreated” in this group. In addition, not only do psychotropic medications increase the risk of metabolic syndrome for these patients, but also lifestyle factors, social deprivation, and a genetic predisposition (1).

**Broad definition of SMI**

Please allow us to explain why we chose a broad SMI definition. As you are undoubtedly familiar with, there are not any consensus criteria for SMI (2). Generally, SMI could be defined by diagnosis, disability and duration (2). In the existing literature when SMI is defined, the predominant diagnoses are psychotic disorders, bipolar disorders, and major depression with psychotic features (2). However, sometimes other diagnoses such as personality disorders and severe concurrent diagnoses (psychiatric diagnosis with substance abuse) are included within the category of severe mental illness too (2). Here are a few examples of other registry-based studies that have also used a broad SMI definition: Scheuer et al defined SMI as schizophrenia, bipolar disorder, or depression (3) and Jakobsen et al. defined SMI as a bipolar disorder, schizophrenia, schizoaffective disorder, or users of antipsychotic medications (4). In this study, we chose a broad SMI definition as increased mortality is also observed among people with personality disorders and major depression (5), and we hypothesized that the symptoms of these disorders could have influenced the treatment too.

Our intention was not to include patients with metabolic syndrome only. We agree that people with personality disorders seldom receive psychotropic medications; at least not in high dosages (unless they have other psychiatric comorbidities). However, we contended that the symptoms of personality disorders can influence how these people interact with health professionals, and possibly, therefore, affect their treatment (6). In addition, personality disorders are associated with the development of other psychiatric disorders (6), and when including this group in our SMI definition, we will likely have included some patients who developed other SMI diagnoses during the follow-up. Within each psychiatric diagnosis group, there is marked heterogeneity, but in general people with the most severe disorders are treated in the secondary healthcare sector (mild mental disorders are treated in general practice), which supported our hypothesis that we captured the most severely ill persons in this study.

You have helpfully added statements around the Danish context but I was wanting to know if any physician behaviours are incentivised-which could be driving the patterns you report. In the UK- primary care physicians are incentivised to undertake annual health checks in people with schizophrenia and bipolar disorders (but not for depression or personality disorders)- which drives some of the observed associations for T2DM which we observe here- are there similar initiatives in Denmark which may account for the patterns you observed?

In the study period, there were no special economic incentives for Danish GPs to undertake health checks on people with psychiatric disorders.

**Visualization of the figures:**

In the first review round, it was suggested that our main figures could be improved for clarity. Based on this we have made a few adjustments to the figures (i.e., changing the x-axis to years instead of months). We hope that you agree that this makes the figures clearer.

1.	Nielsen RE, Banner J, Jensen SE. Cardiovascular disease in patients with severe mental illness. Nature Reviews Cardiology. 2021;18(2):136-45.

2.	Suijkerbuijk YB, Schaafsma FG, van Mechelen JC, Ojajärvi A, Corbière M, Anema JR. Interventions for obtaining and maintaining employment in adults with severe mental illness, a network meta‐analysis. Cochrane Database Syst Rev. 2017(9).

3.	Scheuer SH, Kosjerina V, Lindekilde N, Pouwer F, Carstensen B, Jørgensen ME, et al. Severe mental illness and the risk of diabetes complications. A nationwide register-based cohort study. The Journal of Clinical Endocrinology & Metabolism. 2022.

4.	Jakobsen L, Terkelsen CJ, Christiansen EH, Maeng M, Jensen LO, Veien K, et al. Severe Mental Illness and Clinical Outcome After Primary Percutaneous Coronary Intervention. The American journal of cardiology. 2017;120(4):550-5.

5.	Nordentoft M, Wahlbeck K, Hällgren J, Westman J, Ösby U, Alinaghizadeh H, et al. Excess Mortality, Causes of Death and Life Expectancy in 270,770 Patients with Recent Onset of Mental Disorders in Denmark, Finland and Sweden. PLoS One. 2013;8(1):e55176.

6.	Tyrer P, Reed GM, Crawford MJ. Classification, assessment, prevalence, and effect of personality disorder. Lancet (London, England). 2015;385(9969):717-26.
