# Supplementary material for: Diabetes treatment for persons with severe mental illness: A registry-based cohort study to explore medication treatment differences for persons with type 2 diabetes with and without severe mental illness
Source: PLoS One. 2023 Jun 13;18(6):e0287017. doi: 10.1371/journal.pone.0287017 (PMC10263345; doi:10.1371/journal.pone.0287017)
Supplement: Peer review history

# Peer review: Decision Letter 0

**DOI:** 10.1371/journal.pone.0287017.r001

**Authors:** Kyoung-Sae Na

11 Nov 2022

PONE-D-22-16756Diabetes treatment for persons with severe mental illness: A registry-based cohort study to explore medication treatment differences for persons with type 2 diabetes with and without severe mental illnessPLOS ONE

Dear Dr. Bakkedal,

Thank you for submitting your manuscript to PLOS ONE. After careful consideration, we feel that it has merit but does not fully meet PLOS ONE’s publication criteria as it currently stands. Therefore, we invite you to submit a revised version of the manuscript that addresses the points raised during the review process.

==============================Three reviewers thoroughly read your paper and raised important concerns, particularly in perspective of methodology. As some of the reviewers' comments (e.g., diagnostic criteria and information on prescribed psychotropic medications) do not seem to be resolved by rewriting the paper, please note that this invitation does not  guarantee final acceptance of this paper.==============================

Please submit your revised manuscript by Dec 26 2022 11:59PM. If you will need more time than this to complete your revisions, please reply to this message or contact the journal office at plosone@plos.org. When you're ready to submit your revision, log on to https://www.editorialmanager.com/pone/ and select the 'Submissions Needing Revision' folder to locate your manuscript file.

Please include the following items when submitting your revised manuscript:

- A rebuttal letter that responds to each point raised by the academic editor and reviewer(s). You should upload this letter as a separate file labeled 'Response to Reviewers'.
- A marked-up copy of your manuscript that highlights changes made to the original version. You should upload this as a separate file labeled 'Revised Manuscript with Track Changes'.
- An unmarked version of your revised paper without tracked changes. You should upload this as a separate file labeled 'Manuscript'.

If you would like to make changes to your financial disclosure, please include your updated statement in your cover letter. Guidelines for resubmitting your figure files are available below the reviewer comments at the end of this letter.

If applicable, we recommend that you deposit your laboratory protocols in protocols.io to enhance the reproducibility of your results. Protocols.io assigns your protocol its own identifier (DOI) so that it can be cited independently in the future. For instructions see: https://journals.plos.org/plosone/s/submission-guidelines#loc-laboratory-protocols. Additionally, PLOS ONE offers an option for publishing peer-reviewed Lab Protocol articles, which describe protocols hosted on protocols.io. Read more information on sharing protocols at https://plos.org/protocols?utm_medium=editorial-email&utm_source=authorletters&utm_campaign=protocols.

We look forward to receiving your revised manuscript.

Kind regards,

Kyoung-Sae Na, M.D., Ph.D.

Academic Editor

PLOS ONE

Journal Requirements:

When submitting your revision, we need you to address these additional requirements.

1. Please ensure that your manuscript meets PLOS ONE's style requirements, including those for file naming. The PLOS ONE style templates can be found at

https://journals.plos.org/plosone/s/file?id=wjVg/PLOSOne_formatting_sample_main_body.pdf and

https://journals.plos.org/plosone/s/file?id=ba62/PLOSOne_formatting_sample_title_authors_affiliations.pdf.

2. You indicated that ethical approval was not necessary for your study. We understand that the framework for ethical oversight requirements for studies of this type may differ depending on the setting and we would appreciate some further clarification regarding your research. Could you please provide further details on why your study is exempt from the need for approval and confirmation from your institutional review board or research ethics committee (e.g., in the form of a letter or email correspondence) that ethics review was not necessary for this study? Please include a copy of the correspondence as an ""Other"" file."

3.Please provide additional details regarding participant consent. In the ethics statement in the Methods and online submission information, please ensure that you have specified (1) whether consent was informed and (2) what type you obtained (for instance, written or verbal, and if verbal, how it was documented and witnessed). If your study included minors, state whether you obtained consent from parents or guardians. If the need for consent was waived by the ethics committee, please include this information.

If you are reporting a retrospective study of medical records or archived samples, please ensure that you have discussed whether all data were fully anonymized before you accessed them and/or whether the IRB or ethics committee waived the requirement for informed consent. If patients provided informed written consent to have data from their medical records used in research, please include this information.

4. Thank you for stating in your Funding Statement:

“CB received funding from CACHET (Copenhagen Center for Health Technology, 500110) and the SOFIA project (supported by the Novo Nordisk Foundation, NNF16OC0022038). The funders had no role in study design, data collection and analysis, decision to publish, or preparation of the manuscript.

https://www.cachet.dk/

https://ifsv.ku.dk/om instituttet/almen_medicin/forskning/sofia/”

Please provide an amended statement that declares *all* the funding or sources of support (whether external or internal to your organization) received during this study, as detailed online in our guide for authors at http://journals.plos.org/plosone/s/submit-now.  Please also include the statement “There was no additional external funding received for this study.” in your updated Funding Statement.

Please include your amended Funding Statement within your cover letter. We will change the online submission form on your behalf.

5. Thank you for stating the following in the Competing Interests section:

‘I have read the journal's policy and the authors of this manuscript have the following competing interests: FP has served as a consultant, on advisory boards or as educator for AstraZeneca, Novo Nordisk, Boehringer Ingelheim, Sanofi, Mundipharma, MSD, Novartis, Amgen and has received research grants to institution from Novo Nordisk, Boehringer Ingelheim, Amgen and AstraZeneca.

CB has been employed as part of her specialization in Clinical Pharmacology in Novo Nordisk in a 1-year position that ended in 2018”

We note that one or more of the authors are employed by a commercial company: AstraZeneca, Novo Nordisk, Boehringer Ingelheim, Sanofi, Mundipharma, MSD, Novartis, Amgen

a.            Please provide an amended Funding Statement declaring this commercial affiliation, as well as a statement regarding the Role of Funders in your study. If the funding organization did not play a role in the study design, data collection and analysis, decision to publish, or preparation of the manuscript and only provided financial support in the form of authors' salaries and/or research materials, please review your statements relating to the author contributions, and ensure you have specifically and accurately indicated the role(s) that these authors had in your study. You can update author roles in the Author Contributions section of the online submission form.

Please also include the following statement within your amended Funding Statement.

“The funder provided support in the form of salaries for authors [insert relevant initials], but did not have any additional role in the study design, data collection and analysis, decision to publish, or preparation of the manuscript. The specific roles of these authors are articulated in the ‘author contributions’ section.”

If your commercial affiliation did play a role in your study, please state and explain this role within your updated Funding Statement.

b. Please also provide an updated Competing Interests Statement declaring this commercial affiliation along with any other relevant declarations relating to employment, consultancy, patents, products in development, or marketed products, etc. 

Within your Competing Interests Statement, please confirm that this commercial affiliation does not alter your adherence to all PLOS ONE policies on sharing data and materials by including the following statement: "This does not alter our adherence to  PLOS ONE policies on sharing data and materials.” (as detailed online in our guide for authors http://journals.plos.org/plosone/s/competing-interests) . If this adherence statement is not accurate and  there are restrictions on sharing of data and/or materials, please state these. Please note that we cannot proceed with consideration of your article until this information has been declared.

Please include both an updated Funding Statement and Competing Interests Statement in your cover letter. We will change the online submission form on your behalf.

6. We note that you have included the phrase “data not shown” in your manuscript. Unfortunately, this does not meet our data sharing requirements. PLOS does not permit references to inaccessible data. We require that authors provide all relevant data within the paper, Supporting Information files, or in an acceptable, public repository. Please add a citation to support this phrase or upload the data that corresponds with these findings to a stable repository (such as Figshare or Dryad) and provide and URLs, DOIs, or accession numbers that may be used to access these data. Or, if the data are not a core part of the research being presented in your study, we ask that you remove the phrase that refers to these data.

[Note: HTML markup is below. Please do not edit.]

Reviewers' comments:

Reviewer's Responses to Questions

**Comments to the Author**

1. Is the manuscript technically sound, and do the data support the conclusions?

The manuscript must describe a technically sound piece of scientific research with data that supports the conclusions. Experiments must have been conducted rigorously, with appropriate controls, replication, and sample sizes. The conclusions must be drawn appropriately based on the data presented. 

Reviewer #1: No

Reviewer #2: No

Reviewer #3: Partly

**********

2. Has the statistical analysis been performed appropriately and rigorously? 

Reviewer #1: Yes

Reviewer #2: Yes

Reviewer #3: Yes

**********

3. Have the authors made all data underlying the findings in their manuscript fully available?

The PLOS Data policy requires authors to make all data underlying the findings described in their manuscript fully available without restriction, with rare exception (please refer to the Data Availability Statement in the manuscript PDF file). The data should be provided as part of the manuscript or its supporting information, or deposited to a public repository. For example, in addition to summary statistics, the data points behind means, medians and variance measures should be available. If there are restrictions on publicly sharing data—e.g. participant privacy or use of data from a third party—those must be specified.

Reviewer #1: No

Reviewer #2: No

Reviewer #3: Yes

**********

4. Is the manuscript presented in an intelligible fashion and written in standard English?

PLOS ONE does not copyedit accepted manuscripts, so the language in submitted articles must be clear, correct, and unambiguous. Any typographical or grammatical errors should be corrected at revision, so please note any specific errors here.

Reviewer #1: Yes

Reviewer #2: Yes

Reviewer #3: Yes

**********

5. Review Comments to the Author

Please use the space provided to explain your answers to the questions above. You may also include additional comments for the author, including concerns about dual publication, research ethics, or publication ethics. (Please upload your review as an attachment if it exceeds 20,000 characters)

Reviewer #1: Thank you for the opportunity to review this interesting article in which the authors report the findings of a cohort study examining diabetes and CVD medication prescribing among people with T2DM. The article is well written and very clear. The strength of this study lies in the analyses of diabetes medication prescribing, with authors looking at prescription redemption over a long period of time while accounting for diabetes duration. The findings on CVD prescribing are interesting, but subject to more limitations, since the authors did not have information on history of hypertension or cholesterol/lipid levels and so did not look at prescribing according to presence/absence of cardiovascular risk factors. Although they did have information on history of established cardiovascular disease (such as atherosclerotic cardiovascular disease), they did not compared CVD prescribing by SMI status in this sub-group. Findings are presented for all SMI combined, and as the authors state, it would be helpful to examine specific subtypes with respect to diabetes and CVD prescribing in future studies. Despite these limitations, this article makes an important contribution to a very important and under-researched area. I have just a few suggestions on how the article may be further improved

Major comments:

A key limitation of the findings on CVD prescribing is the lack of information on cardiovascular risk factors, including hypertension and history of lipid abnormalities and the fact that the authors haven’t looked at whether people with CVD risk factors or established disease are being appropriately treated – i.e. they simply looks at redemption of prescriptions. They comment on the rate of prescription redemption with respect to the median values of lipid measurements, but these measures do not seem to have been available on the whole cohort and this should be highlighted on page 10. Also, there are of course limitations in relating aggregate lipid measurements to aggregate rates of prescription post-diabetes diagnosis.

Did the authors consider performing a sensitivity analysis to look at findings among those with established CVD (i.e. history of major cardiovascular disease event), to look at cardio drugs post DM diagnosis. This may be interesting, even in the inevitably smaller sample.

On page 20 of discussion, the authors hypothesise that there is a higher mortality in the SMI group, leading to a survival bias with “more robust” people with SMI being included in the later follow-up. The difference in mortality is likely, but again presumably the authors could simply look at this to support this statement with data rather than hypothesis?

The contrasting findings on receipt of diabetes medication prescriptions versus CVD medication prescriptions is very interesting and to some readers this contrast may be surprising. The authors very nicely discuss the findings with respect to other relevant literature. However, although they propose some explanations for the diabetes medication findings, they don’t really provide possible suggestions for the observed CVD medication prescribing. Can the authors expand on this a little?

What do the authors consider to be the implications of the research for health policy/practice and research, particularly with a view to understanding the possible opposing results on diabetes and CVD prescribing?

The authors do not state in the methods whether they adhered to suitable reporting guidelines (i.e. RECORD).

Minor comments:

Supplementary tables – could add footnote to indicate what variables RRs are adjusted for.

Reviewer #2: This is a study of pharmacoutilization in diabetic patients with or without severe mental illness. The area of research is interesting and potentially relevant for public health since the need to understand the causes for higher cardiovascular morbidity and mortality in people with SMI. The methods for defining cases and controls is sound, the sample representative and the duration of follow-up adequate. The authors find that diabetic patients with SMI are prescribed more antidiabetic medications and less cardiovascular medications in the initial years after the diagnosis of diabetes.

However, there is a huge methodological issue: diabetes is diagnosed out of a single assessment of elevated HbA1c or glycemia levels.

International criteria for diagnosing diabetes require either two independent assessment of glycemia ≥ 7 mmol/l or HbA1c ≥ 6.5, or one assessment coupled with the presence of symptoms of diabetes, such as polyuria, polydipsia, and glucosuria. A random plasma glucose ≥ 11 mmol/l could diagnose diabetes only in patients with symptoms of hyperglycemia. Recently, a study highlighted that high levels of both glycemia and HbA1c in the same sample also allows diagnosing diabetes with high accuracy (Selvin et al., 2018). However, I am afraid that diagnosing diabetes out of a single blood test may lead to false positives.

Although the authors, not being able to tease out the blood tests performed in a fasting condition, correctly considered only remarkably high glycemic values to reduce type I error, still a single measurement of one biomarker only is insufficient for a diagnosis of diabetes.

As a matter of fact, a putative explanation of finding more prescriptions of antidiabetic medications in the SMI group might be that these patients have been more frequently diagnosed with diabetes over time than the non-SMI counterpart.

The authors have access to ICD diagnoses in this cohort. Although it’s true that employing a diagnosis based on biomarker levels rules out undiagnosed diabetes, I guess that they should include patients based on relevant ICD codes instead of using an incorrect definition based on a single biomarker observation.

Reviewer #3: Comments on paper PONE-D-22-16756

This is an interesting paper which uses high quality data from Danish registries to assess prescribing in severe mental illnesses.

My main concern with the analysis is that the authors did not seem to have any information on prescribed psychotropic medications- this would include atypical antipsychotic medications, some of which are known to be associated with a high risk of developing T2DM and metabolic syndrome (eg. in particular Olanzapine, Clozapine)/ be associated with CVD risk. In some patients where clinicians are concerned about side effects drugs with lower propensity for CVD risk may be suggested for switching/ initiation- in fact this is recommended in WHO guidelines for the management of physical health in SMI- see this link https://www.who.int/publications/i/item/978-92-4-155038-3 Also using adjunct Metformin is recommended for people who are overweight or obese (see the WHO link).

This therefore means that it is impossible to know if the patterns they observed in their dataset simply reflects clinicians following guidelines and/ or are confounded by psychotropic medication prescribing.

I have some further comments below which the authors may wish to consider and it may strengthen the paper:

Abstract - this ends rather abruptly . I suggest the authors add a sentence at the end summarising the main conclusions

To understand the findings it would be helpful to have more detail on care provided in Denmark . Are more complex patients with T2DM referred on to secondary care ? Do mental health care providers also look after physical health in SMI patients . What are the specific care pathways and is there evidence in inequalities in these impacting people with SMI ?

Figures are a little unclear - please provide a label for the x axis and a footnote for acronyms in the figure . For example in figure 2 what is A10BD?

In general the acronyms in the text make it less readable- please consider spelling out where possible

**********

6. PLOS authors have the option to publish the peer review history of their article (what does this mean?). If published, this will include your full peer review and any attached files.

If you choose “no”, your identity will remain anonymous but your review may still be made public.

**Do you want your identity to be public for this peer review?** For information about this choice, including consent withdrawal, please see our Privacy Policy.

Reviewer #1: **Yes: **Caroline Jackson

Reviewer #2: No

Reviewer #3: No

**********

[NOTE: If reviewer comments were submitted as an attachment file, they will be attached to this email and accessible via the submission site. Please log into your account, locate the manuscript record, and check for the action link "View Attachments". If this link does not appear, there are no attachment files.]

While revising your submission, please upload your figure files to the Preflight Analysis and Conversion Engine (PACE) digital diagnostic tool, https://pacev2.apexcovantage.com/. PACE helps ensure that figures meet PLOS requirements. To use PACE, you must first register as a user. Registration is free. Then, login and navigate to the UPLOAD tab, where you will find detailed instructions on how to use the tool. If you encounter any issues or have any questions when using PACE, please email PLOS at figures@plos.org. Please note that Supporting Information files do not need this step.

# Author comment: Author response to Decision Letter 0

**DOI:** 10.1371/journal.pone.0287017.r002

4 Jan 2023

Thanks for your comments and for letting me revising the manuscript. 

Please refer to my rebuttal letter, that I have attached to this revision.

Attachment Submitted filename: Response to Reviewers.docx Click here for additional data file.

# Peer review: Decision Letter 1

**DOI:** 10.1371/journal.pone.0287017.r003

**Authors:** Kyoung-Sae Na

24 Feb 2023

PONE-D-22-16756R1Diabetes treatment for persons with severe mental illness: A registry-based cohort study to explore medication treatment differences for persons with type 2 diabetes with and without severe mental illnessPLOS ONE

Dear Dr. Bakkedal,

Thank you for submitting your manuscript to PLOS ONE. After careful consideration, we feel that it has merit but does not fully meet PLOS ONE’s publication criteria as it currently stands. Therefore, we invite you to submit a revised version of the manuscript that addresses the points raised during the review process.

==============================

The authors subtantially improved their manuscript. However, the reviewer still raises an important issue about the definition of SMI. Please consider it carefully and revise the paper appropriately.

==============================

Please submit your revised manuscript by Apr 10 2023 11:59PM. If you will need more time than this to complete your revisions, please reply to this message or contact the journal office at plosone@plos.org. When you're ready to submit your revision, log on to https://www.editorialmanager.com/pone/ and select the 'Submissions Needing Revision' folder to locate your manuscript file.

Please include the following items when submitting your revised manuscript:

- A rebuttal letter that responds to each point raised by the academic editor and reviewer(s). You should upload this letter as a separate file labeled 'Response to Reviewers'.
- A marked-up copy of your manuscript that highlights changes made to the original version. You should upload this as a separate file labeled 'Revised Manuscript with Track Changes'.
- An unmarked version of your revised paper without tracked changes. You should upload this as a separate file labeled 'Manuscript'.

If you would like to make changes to your financial disclosure, please include your updated statement in your cover letter. Guidelines for resubmitting your figure files are available below the reviewer comments at the end of this letter.

If applicable, we recommend that you deposit your laboratory protocols in protocols.io to enhance the reproducibility of your results. Protocols.io assigns your protocol its own identifier (DOI) so that it can be cited independently in the future. For instructions see: https://journals.plos.org/plosone/s/submission-guidelines#loc-laboratory-protocols. Additionally, PLOS ONE offers an option for publishing peer-reviewed Lab Protocol articles, which describe protocols hosted on protocols.io. Read more information on sharing protocols at https://plos.org/protocols?utm_medium=editorial-email&utm_source=authorletters&utm_campaign=protocols.

We look forward to receiving your revised manuscript.

Kind regards,

Kyoung-Sae Na, M.D., Ph.D.

Academic Editor

PLOS ONE

[Note: HTML markup is below. Please do not edit.]

Reviewers' comments:

Reviewer's Responses to Questions

**Comments to the Author**

1. If the authors have adequately addressed your comments raised in a previous round of review and you feel that this manuscript is now acceptable for publication, you may indicate that here to bypass the “Comments to the Author” section, enter your conflict of interest statement in the “Confidential to Editor” section, and submit your "Accept" recommendation.

Reviewer #1: All comments have been addressed

Reviewer #3: (No Response)

**********

2. Is the manuscript technically sound, and do the data support the conclusions?

The manuscript must describe a technically sound piece of scientific research with data that supports the conclusions. Experiments must have been conducted rigorously, with appropriate controls, replication, and sample sizes. The conclusions must be drawn appropriately based on the data presented. 

Reviewer #1: Yes

Reviewer #3: Partly

**********

3. Has the statistical analysis been performed appropriately and rigorously? 

Reviewer #1: Yes

Reviewer #3: Yes

**********

4. Have the authors made all data underlying the findings in their manuscript fully available?

The PLOS Data policy requires authors to make all data underlying the findings described in their manuscript fully available without restriction, with rare exception (please refer to the Data Availability Statement in the manuscript PDF file). The data should be provided as part of the manuscript or its supporting information, or deposited to a public repository. For example, in addition to summary statistics, the data points behind means, medians and variance measures should be available. If there are restrictions on publicly sharing data—e.g. participant privacy or use of data from a third party—those must be specified.

Reviewer #1: Yes

Reviewer #3: Yes

**********

5. Is the manuscript presented in an intelligible fashion and written in standard English?

PLOS ONE does not copyedit accepted manuscripts, so the language in submitted articles must be clear, correct, and unambiguous. Any typographical or grammatical errors should be corrected at revision, so please note any specific errors here.

Reviewer #1: Yes

Reviewer #3: Yes

**********

6. Review Comments to the Author

Please use the space provided to explain your answers to the questions above. You may also include additional comments for the author, including concerns about dual publication, research ethics, or publication ethics. (Please upload your review as an attachment if it exceeds 20,000 characters)

Reviewer #1: (No Response)

Reviewer #3: The manuscript is much improved. However my concerns around your general approach, leading to the conclusions, remain. The main issue is that your definition of 'SMI' is very broad- usually this would just encompass people with schizophrenia-spectrum and bipolar disorders (who are more likely prescribed antipsychotics- including those associated with a higher risk of metabolic syndrome- which as I suggested in my previous review- may be driving the observed patterns). However you have included people with personality disorders and depression in your 'SMI' definition. Prescribing and risks for CVD/ diabetes may be quite different for these other diagnostic groups. Personality disorders in particular are axis 2 diagnoses and this group may not receive any psychotropic medications. If you are unable to look at types of psychotropic medications prescribed then at the very least I would suggest stratifying or undertaking analyses by psychiatric diagnosis- I would suggest keeping schizophrenia and bipolar disorders separate to depression, and separate to personality disorders. My preference would be to see both stratified analyses by psychiatric diagnoses and a consideration of psychotropic medication prescribing (in particular antipsychotic medications).

You have helpfully added statements around the Danish context but I was wanting to know if any physician behaviours are incentivised-which could be driving the patterns you report. In the UK- primary care physicians are incentivised to undertake annual health checks in people with schizophrenia and bipolar disorders (but not for depression or personality disorders)- which drives some of the observed associations for T2DM which we observe here- are there similar initiatives in Denmark which may account for the patterns you observed?

**********

7. PLOS authors have the option to publish the peer review history of their article (what does this mean?). If published, this will include your full peer review and any attached files.

If you choose “no”, your identity will remain anonymous but your review may still be made public.

​

**Do you want your identity to be public for this peer review?** For information about this choice, including consent withdrawal, please see our Privacy Policy.

Reviewer #1: **Yes: **Caroline Jackson

Reviewer #3: No

**********

[NOTE: If reviewer comments were submitted as an attachment file, they will be attached to this email and accessible via the submission site. Please log into your account, locate the manuscript record, and check for the action link "View Attachments". If this link does not appear, there are no attachment files.]

While revising your submission, please upload your figure files to the Preflight Analysis and Conversion Engine (PACE) digital diagnostic tool, https://pacev2.apexcovantage.com/. PACE helps ensure that figures meet PLOS requirements. To use PACE, you must first register as a user. Registration is free. Then, login and navigate to the UPLOAD tab, where you will find detailed instructions on how to use the tool. If you encounter any issues or have any questions when using PACE, please email PLOS at figures@plos.org. Please note that Supporting Information files do not need this step.

# Author comment: Author response to Decision Letter 1

**DOI:** 10.1371/journal.pone.0287017.r004

7 Apr 2023

Please refer to the attached file named "Response to reviewers". Thank you very much for your work on this article.

Attachment Submitted filename: Response to reviewers.docx Click here for additional data file.

# Editor report: Decision Letter 2

**DOI:** 10.1371/journal.pone.0287017.r005

**Authors:** Kyoung-Sae Na

30 May 2023

Diabetes treatment for persons with severe mental illness: A registry-based cohort study to explore medication treatment differences for persons with type 2 diabetes with and without severe mental illness

PONE-D-22-16756R2

Dear Dr. Bakkedal,

We’re pleased to inform you that your manuscript has been judged scientifically suitable for publication and will be formally accepted for publication once it meets all outstanding technical requirements.

Within one week, you’ll receive an e-mail detailing the required amendments. When these have been addressed, you’ll receive a formal acceptance letter and your manuscript will be scheduled for publication.

An invoice for payment will follow shortly after the formal acceptance. To ensure an efficient process, please log into Editorial Manager at http://www.editorialmanager.com/pone/, click the 'Update My Information' link at the top of the page, and double check that your user information is up-to-date. If you have any billing related questions, please contact our Author Billing department directly at authorbilling@plos.org.

If your institution or institutions have a press office, please notify them about your upcoming paper to help maximize its impact. If they’ll be preparing press materials, please inform our press team as soon as possible -- no later than 48 hours after receiving the formal acceptance. Your manuscript will remain under strict press embargo until 2 pm Eastern Time on the date of publication. For more information, please contact onepress@plos.org.

Kind regards,

Kyoung-Sae Na, M.D., Ph.D.

Academic Editor

PLOS ONE

Additional Editor Comments (optional):

Reviewers' comments:

# Editor report: Acceptance letter

**DOI:** 10.1371/journal.pone.0287017.r006

**Authors:** Kyoung-Sae Na

4 Jun 2023

PONE-D-22-16756R2 

Diabetes treatment for persons with severe mental illness: A registry-based cohort study to explore medication treatment differences for persons with type 2 diabetes with and without severe mental illness 

Dear Dr. Bakkedal:

I'm pleased to inform you that your manuscript has been deemed suitable for publication in PLOS ONE. Congratulations! Your manuscript is now with our production department. 

If your institution or institutions have a press office, please let them know about your upcoming paper now to help maximize its impact. If they'll be preparing press materials, please inform our press team within the next 48 hours. Your manuscript will remain under strict press embargo until 2 pm Eastern Time on the date of publication. For more information please contact onepress@plos.org.

If we can help with anything else, please email us at plosone@plos.org. 

Thank you for submitting your work to PLOS ONE and supporting open access. 

Kind regards, 

PLOS ONE Editorial Office Staff

on behalf of

Dr. Kyoung-Sae Na 

Academic Editor

PLOS ONE
